# People are curious about immoral and morally ambiguous others

**DOI:** 10.1038/s41598-023-30312-9

**Published:** 2023-05-05

**Authors:** Jordan Wylie, Ana Gantman

**Affiliations:** 1 Department of Psychology and Neuroscience, Boston College, Chestnut Hill, MA 02467 USA; 2 The City University of New York Graduate Center, New York, USA; 3 Brooklyn College, New York, USA

**Keywords:** Human behaviour, Psychology

## Abstract

Looking to the popularity of superheroes, true crime stories, and anti-heroic characters like Tony Soprano, we investigated whether moral extremity, especially moral badness, piques curiosity. Across five experiments (*N* = 2429), we examine moral curiosity, testing under what conditions the moral minds of others spark explanation-seeking behavior. In Experiment 1, we find that among the most widely watched Netflix shows in the US over a five-month period, the more immoral the protagonist, the more hours people spent watching. In Experiments 2a and 2b, we find that when given a choice to learn more about morally good, bad, ambiguous, or average others, people preferred to learn more about morally extreme people, both good and bad. Experiment 3 reveals that people are more curious for explanations about (vs. descriptions of) morally bad and ambiguous people compared to morally good ones. Finally, Experiment 4 tests the uniqueness of curiosity for moral ambiguity. We find that people are more drawn to moral rather than aesthetic ambiguity, suggesting that ambiguity, which is cognitively taxing and sometimes avoided, preferentially engenders information seeking in the moral domain. These findings suggest deviations from moral normativity, especially badness, spur curiosity. People are curious about immorality and agents who differ from the norm.

## Introduction

The pursuit of moral goodness is a central tenet of humanity—the rules that proscribe it emerge across time, cultures, and religions^1^. Yet, there is something about being relentlessly morally good that is plainly boring. This fact has been famously pointed out by philosopher Susan Wolf. Wolf argues that people have an aversion to faultlessly morally good others, or “moral saints”^2^. This perspective is in some ways provocative—moral goodness is foundational to individuals’ identity and character, and our moral selves are our truest selves^3^. But on the other hand, it is unsurprising. People are often drawn to ambiguous or even immoral themes and content. This interest is reflected in the popularity of TV characters like Dexter Morgan (serial killer) and Tony Soprano (mobster), movies like Batman (vigilante), or in the rise in popularity of true crime^4^. That is, by and large, people frequently spend time thinking about these morally bad and ambiguous characters. Here, we take this phenomenon seriously, and we investigate what features of moral badness and ambiguity trigger curiosity above and beyond moral goodness.

### Moral curiosity

From *Shakespeare* to *The Sopranos*, the documented and sustained interest in morally ambiguous or bad themes and characters (see^1,5^ for discussion) suggests that they pique our curiosity. And curiosity is fundamental to cognition (see^6^ for review). It is associated with learning and memory^7,8^, exploration behaviors^9,10^, creativity^11^, and more. Curiosity is one of a suite of epistemic emotions—emotions for which the catalyst is the individual’s own cognitive state. These emotions help orient us toward effective ways of understanding and exploring our environments. For example, emotions like surprise, confusion, and curiosity are each considered epistemic emotions because they are triggered by cognitions related to beliefs about knowledge^12^. Such emotions are critical to problem solving, knowledge acquisition, exploration, and other largely epistemic functions^13,14^. They support belief-acquisition processes by making salient the times when contradictions and inconsistencies are relevant to a given task. We argue that moral ambiguity and moral deviance can trigger these epistemic emotions. Specifically, curiosity aimed at moral content, or moral curiosity, motivates knowledge and approach related behaviors that allow people to learn about critical social information—the minds and outcomes of those who break the rules. Critically, in the case of moral curiosity, epistemic emotions may drive people to approach morally bad content we might otherwise expect them to avoid (e.g.,^15,16^).

A combination of research and epistemological philosophy have outlined the characteristic features of an epistemic emotion (e.g.,^17^). Here, we aim to use this framework and extend it to help understand what appears to be a puzzle. We expect that people seek to maximize pleasure and minimize pain^18^, yet people are drawn to moral badness and ambiguity. Moral ambiguity and badness trigger curiosity to approach, acquire, appraise, and understanding that information––ultimately supporting the maintenance and construction of a coherent moral worldview (see also^19,20^). This is because immoral and morally ambiguous minds provide valuable information about how the world works. It is also possible that moral ambiguity is a unique trigger of moral curiosity because it is uncategorized and un-resolved (e.g.,^21^), and in the moral domain, people often prefer clarity and reject nuance (e.g.,^22^), and so seek to resolve it. As a result of encountering these more volatile states (see^23^), people may be particularly motivated to search for explanations that afford categorization and sense-making, and thereby resolve the ambiguity, particularly in the moral domain.

### Explanations in morality

Explanations are fundamental to cognition. And it’s no wonder—asking “why” facilitates cognitive development and helps humans make sense of their worlds^24^. Unlike similar epistemic behaviors like information-seeking (e.g., see^25,26^), explanation-seeking takes the drive for knowledge one-step further to identify the deeper reasons *behind* the target of curiosity. Explanation-seeking demonstrates that often the fact alone is not enough, humans are hungry to know “why”. Indeed, even young children often display this need for “why”^24^. When seeking explanations, children do not find mere attention sufficient—they really do want to know why^27^, will ask follow-up questions when an unsatisfactory answer is given^28^, and show preferences towards information sources that provide the concrete sorts of reasons they are after^29^.

Like the other “why” questions that populate Reddit’s Too Afraid to Ask (www.reddit.com/r/tooafraidtoask) or Explain like I’m Five (ELI5; www.reddit.com/r/explainlikeimfive; see also^30^), a quick search reveals that people are eager to understand the reasons that underlie or explain everyday moral phenomena and the moral decisions of others. This includes themes surrounding real world moral villains (e.g., many questions like: “What [were] Hitler's motives?”; “Just why, why was Stalin himself seemingly unimaginably evil?”). While much of the general structure of day-to-day life of an adult is known, the minds of others are consistently opaque to us. And yet, we are driven to understand and engage with the minds of others. We have dedicated neural machinery that helps to support thinking about the intentions and thoughts of others, which are critical aspects of moral reasoning^31,32^. Mind information is especially important for morality (see^33^), and it likely elicits curiosity because understanding others is critical to adaptive social cognition and survival (e.g.,^34,35^). Questions about the immoral acts and intentions of other people pique curiosity because they offer information about how other people see the world, and information about how good and evil operate. That is, explanations—insight into the minds of others—may be the most persistent epistemic motives that adults have because it is always relevant information to their understanding of how the world works (not to mention who we decide to trust;^36,37^).

### The present research

The present research integrates multiple theoretical perspectives and multiple methods to explore moral curiosity. In Experiment 1, we investigate whether there is a relationship between the hours people spent watching and the immorality of a program’s protagonist among the most widely watched Netflix shows in the US over a five-month period. In Experiments 2a and 2b, we investigate who people choose to learn more about: morally good, bad, ambiguous, or average others. Experiment 3 tests whether people prefer explanations (vs. descriptions) when they have the opportunity to learn about bad people. Finally, Experiment 4 tests the uniqueness of curiosity for moral ambiguity compared to aesthetic ambiguity (another domain with no criteria for accuracy but potential consensus).

To our knowledge, this is the first set of experiments to systematically investigate the moral curiosity drive in adults. We use real world publicly available data (Study 1) combined with behavioral and survey methods (Experiments 2–4) to test whether moral ambiguity and moral badness are unique triggers of moral curiosity and explore some of its potential functions. All pre-registrations, materials, data, and analysis code are available on the Open Science Framework. Sample size was always preregistered and determined in advance. We report all preregistered measures and any preregistered analyses that are not in the main text in the Supplemental Materials; analyses that were not preregistered are described as exploratory. All analyses were conducted using R statistical analysis software^38^. Experiments 2–4 use the ‘lme4’ package^39^ and the ‘lmerTest’ package^40^ to compute model *p*-values and use Satterthwaite approximation to calculate degrees of freedom. Data collection procedures for all studies were approved by the Queens College IRB, and all research was performed in accordance with relevant guidelines and regulations. Each survey was distributed via Qualtrics survey software.

### Study 1

People often engage with moral content in fictional worlds and may feel freer to act on this curiosity in fiction than in real life (see^41^). To better understand what kind of moral content is engaging for people, we collated data from Netflix’s publicly available, most viewed TV shows and movies and had an independent sample of participants rate the morality of the main characters. We preregistered two primary analyses: We aimed to measure the proportion of these most popular shows/movies categorized that have an immoral compared to moral protagonist, and to test the correlation between hours watched and the morality of the protagonist. We predicted that a larger proportion of the shows/movies in the top ten on Netflix would feature an immoral, rather than morally good, protagonist (excluding children’s programming). We also preregistered testing for relationships among: Moral character judgments of the protagonist, perceptions of learning from the program, and hours watched for each of the shows/movies. Further, previous research suggests that engaging with other’s motives and feelings is effortful^42^, and that people tend to enjoy engaging with media that features characters similar to themselves (e.g.,^43^; see also^44^). As such, we also measured and explored relationships between effort required to watch a show/movie, similarity to the protagonist, and hours watched. This allowed us to explore the kinds of content that draw people in—with a real-life measure of engagement, hours spent watching.

## Method

### Design

Beginning in November 2021, when Netflix began releasing the data, we recorded the top ten movie and TV shows (by hours viewed) from the Netflix catalog in the United States (Netflix, https://top10.netflix.com/) up until March 2022. This period of five months (determined a priori and preregistered) provided a total of 380 TV shows and movies of which 133 were unique (when excluding sequels). We then used these 133 TV shows and movies as stimuli for a survey and had participants rate a random subset of 25 of these 133 shows and movies on the morality of the protagonist, their perceived similarity, the effort required to watch the show or movie, and how much they perceived learning from the show or movie. Participants only provided ratings for shows and movies that they indicated they were familiar with.

### Participants

We preregistered a recruitment goal of 10 ratings per show, a minimum of 5 ratings per show, and inclusion criteria of United States residence, 99% approval rating on Prolific, and at least 5 previous submissions. We recruited 200 adult (18 and older) participants initially to try to meet the preregistered 5 ratings per show. However, a subset of shows were not widely recognized by participants. As such we recruited two other waves of 600 participants without viewing dependent variables, for a total sample size of 815 before pre-registered attention check exclusions and 808 after removing those who failed the attention check question. With the exception of *N* = 32 shows, all shows had a minimum of 5 independent raters. Participants provided informed consent and were paid $0.53 on average for their participation.

## Materials

### TV shows and movies

We created a list of 133 unique shows from the Netflix ratings of most watched shows. The full list of shows is available on the project’s OSF page. Participants saw the name of the show only. Given that a small subset of shows (*n* = 32) did not have five unique ratings by participants, we had an independent research assistant review the average moral character judgment rating by participants binarized into either an immoral protagonist or a morally good protagonist. The research assistant used the binary category to see whether it matched the description of the protagonist available on the International Movie Database (IMDB; imdb.com) and so the shows/movies with few ratings did not misrepresent them. We included all shows with ratings with the exception of one show which had no ratings at all (“Brazen”), for a total of 132 shows and movies.

### Familiarity

We first asked participants “Are you familiar with this show/movie?” with two options “yes” and “no”.

### Moral character judgment

If participants were familiar with the TV show or movie, participants were asked “What best describes the morality of the main character of this show/movie?” rated on a scale from 1 = *Extremely morally bad* to 4 = *Morally ambiguous* to 7 = *Extremely morally good*.

### Similarity

Participants also indicated their perceived similarity to the main character. We asked, “How similar to you do you find the main character?” rated on a scale from 1 = *Not at all* to 7 = *Extremely similar to me*.

### Perceived learning

We also asked participants “How much do feel like you learn something from watching this show/movie?” rated on a scale from 1 = *None at all* to 7 = *A lot*.

### Effort

Participants indicated the effort they think it requires to watch the TV show or movie. We asked participants “How much effort does it take to watch or pay attention to this show/movie?” rated on a scale from 1 = *Not at all* to 7 = *A lot of effort*.

### Hours watched

We collected the hours watched for each of the shows across the five-month period directly from Netflix. We collapsed shows that had multiple seasons hit the top ten within the timeframe. We then summed the total hours watched over the five-month period for shows that appeared in multiple weeks. We unintentionally omitted specifying how we would calculate this variable in our preregistration; we did not attempt multiple calculations.

### Procedure

All participants provided informed consent and saw a random selection of 25 TV shows and movies from the list of 132. Participants were presented with the name of the show and the familiarity question. If participants selected “no”, they did not see any other judgments, but skipped to the next show or movie. If participants selected “yes”, they answered the full series of questions listed above (except for hours watched, which came directly from Netflix). Participants were then debriefed and compensated for their time.

## Results

We preregistered two primary analyses: to test the proportion of shows and movies with immoral protagonists, and to test the correlation between protagonist immorality and hours viewed. To investigate whether a larger proportion of the top shows/movies in the United States on Netflix were categorized as having an immoral rather than morally good main character, we binarized the continuous judgment of moral character for the main character in the show (made by Prolific participants). We found that 15% of the popular shows were aimed at children (e.g., Cocomelon). As pre-registered, we considered only programming for adults. We found that 59% of the most popular shows and movies over a five-month period (starting from when Netflix began to release the data) were rated as having an immoral main character, compared to 41% having a morally good main character, which were statistically different from one another (χ^2^ (1, *N* = 2493) = 79.43, *p* < 0.001).

We also preregistered testing for associations between hours viewed, perceived learning (i.e., extent to which people think they learn something from the show/movie), and immorality of the protagonist for familiar shows. We standardized all measured variables and then examined relationships among hours viewed and the preregistered predictors. Critically, there was a significant negative correlation between moral character (a bipolar scale from immoral to morally good) and total hours viewed, *r*(3408) = −0.13, *95% CI* [−0.16, −0.10], *p* < 0.001. There was also a small but significant relationship between effort and morality such that more effort was associated with more immoral characters, *r*(3408) = –0.07, *95% CI* [−0.10, −0.04], *p* < 0.001. Finally, learning was positively related to moral character rating, *r*(3408) = 0.19, *95% CI* [0.16, 0.22], *p* = 0.013. Perceived learning was higher when the protagonist was morally good.

We also conducted exploratory follow-up analyses that included the other predictors (e.g., similarity). We found statistically significant correlations among all variables with the exception of perceived effort of viewing and similarity with the main character, *r*(3421) = 0.01, *p* = 0.511 (see Table 1). We then ran exploratory regression analyses. We specified a linear mixed-effects regression with by-participant random slopes and included each of the predictors simultaneously with hours viewed as the outcome. This model did not converge even after adjusting the optimizer. To address this, we analyzed these relationships using two different methods. First, we used linear regression to predict hours watched and found that the effect of protagonist morality was robust when including all other predictors: When all predictors were entered simultaneously, the more immoral the protagonist of the show, the more hours viewed, *b* =  − 0.18, *SE* = 0.03, *t(*3405) =  − 7.04, *p* < 0.001, *r* =  0.1. Perceived learning (*b* = 0.09, *SE* = 0.02, *p* < 0.001) and effort required to watch (*b* = 0.10, *SE* = 0.02, *p* < 0.001) both positively predicted hours viewed while similarity was no longer a significant predictor. Second, we used Bayesian linear mixed-effects models to test the robustness of these relationships while also including random intercepts for participants. This analysis yielded nearly identical estimates as the linear regression analysis and is reported in full in the Supplemental Material.

**Table 1:** Correlation matrix for the participant ratings and total hours viewed.

Measure	1	2	3	4	5
1. Similarity	–				
2. Effort	− 0.01	–			
3. Perceived Learning	0.39***	0.11***	–		
4. Morality	0.50***	− 0.07***	0.19***	–	
5. Hours viewed	− 0.04*	0.09***	0.05*	− 0.13***	–

*p < 0.05, **p < 0.01, ***p < 0.001. Holm method for *p*-value adjustment. *N* = 3410–3424.

Finally, we also conducted exploratory analyses to rule out confounds related to genre and runtime. For genre, it is possible that it is really about the type of show (drama vs. comedy) rather than the morality of the protagonist. Interestingly, one of the most popular shows during the time of data collection was *You*, which features an often cheeky murderer/stalker, suggesting the relationship between genre and protagonist can be complex. Nevertheless, we created a new variable for genre, using the tags in Netflix and IMBD to create 11 total categories/genre of shows and movies: Crime/mystery, drama, sci-fi/fantasy, comedy, action/adventure, documentary, children, reality, animation, horror, and other variety. We specified genre and protagonist morality as simultaneous predictors of effort in a linear regression. When we account for genre the relationship between morality of the protagonist and effort is no longer present, *b* =  − 0.02, *SE* = 0.02, *t*(3398) =  − 1.09, *p* = 0.278, *r* =  − 0.02, 95% CI [−0.06, 0.02]. However, when we control for genre, the key relationship between morality of the protagonist and hours viewed remains robust, *b* =  − 0.17, *SE* = 0.02, *t*(3398) =  − 7.22, *p* < 0.001,* r* =  − 0.12, *95% CI* [−0.21, −0.12].

Similarly, it is also possible that darker shows are just longer. To rule out this confound, we created a variable for the average time of an episode/movie. When we include our new variable, along with genre, effort, similarity, and perceived learning, the relationship between morality of the protagonist and hours watched remains consistent, *b* =  − 0.15, *SE* = 0.03,* t*(3394) =  − 5.92,* p* < 0.001, *r* =  − 0.10, *95% CI* [−0.20, −0.10].

## Discussion

We found evidence for our pre-registered predictions that people tend to watch television featuring immoral characters. Specifically, a larger proportion of the top 10 most-watched Netflix TV shows and movies made for adults over a five-month period from November 2021 to March 2022 contained an immoral main character. And the more immoral the main character of the show, the more total hours were viewed. This relationship was robust to the inclusion of genre, average runtime, and similarity to the protagonist. Effort and moral character were also negatively correlated unless genre was included in the model, suggesting that it may be some other feature of the kinds of shows that have immoral protagonists that requires more effort to watch rather than the morality of the protagonist themselves. Notably, people reported that they learned more from shows with morally good protagonists. It was surprising to us that effort and perceived learning did not vary together as previous research suggests that effort and learning are linked^45^. It is possible that there is a social desirability constraint around learning from the “bad guy” or that in this domain the idea of learning is tied only to learning a moral lesson, an important feature of learning from fictional worlds^46^. This study provides preliminary evidence that there is a link between immorality and engagement—one that may emerge in spite of the effort it requires to watch shows with immoral characters and may not be apparent to viewers. However, it should be noted that this sample represents a restricted range. We only pooled shows that were among the most watched for a given week during the sampling period (the number of least watched shows on Netflix is unavailable and likely massive) and cannot necessarily infer that this relationship holds across all of Netflix’s programming. We observed that people opt to spend more time with immoral minds on their television screens.

### Experiments 2a and 2b

In Experiments 2a and 2b, we experimentally tested what kinds of moral minds pique people’s curiosity. To do this, we generated novel stimuli—a controlled manipulation of agents’ moral character—to see who people wanted to learn more about. We pit four moral agents against each other in an information-seeking decision task. We chose four moral character types who each provide us with information about the moral world, those who are morally average, who we are most likely to encounter; those who we are morally good, who we may emulate; those who are morally bad, who we must avoid or out-maneuver; and those who are morally ambiguous, who might fool us or be inconsistent. We investigated who people wanted to learn more about most often.

Research suggests that curiosity is especially tuned to expected reward and learning^30,]^^47^. When people feel there is high likelihood of learning, they should also feel curious to know more. We found in Study 1, that ratings of perceived learning were associated with morally good protagonists, yet hours watched, a presumed indicator of interest and investment, was associated with immoral ones. Given this increased engagement with morally bad characters in popular TV and movies, we expected people to opt to learn (rather than self-report) more about morally bad and ambiguous people than good or average ones.

Experiments 2a and 2b used nearly identical designs to examine whether people chose to learn about immoral agents more than moral ones, and to explore the links between curiosity and learning in the moral domain. People select the kind of person they want to learn more about from the four options (morally good, bad, ambiguous, or average). In Experiment 2a, they always chose among all four possible types; in Experiment 2b, they chose between two options at a time. Each experiment breaks the information-seeking behavior into multiple steps (adapted from^48^): First, people chose whose mind to learn more about (explanation-seeking decision), then they rated how curious they were (people tend to seek information when they are curious), how confident they were that they would learn something new (people tend to seek information when they are not confident about the answer;^7^), how interested they were, and how much they expected to learn something about human nature (expectation judgments). We also measured similarity with the novel moral agents during this phase of the trial as previous research suggests that feelings of similarity with other agents predicts engagement with them^44^. Further, we found that similarity was positively correlated with hours watched in Study 1 (see Table 1). As such, we preregistered including this measure as a covariate in all subsequent analyses. We also report models without similarity as a covariate in the supplemental materials.

We also included measures to assess how access to explanations about the motives and minds of different moral agents may lead to different feelings of satisfaction and perceptions of learning. Previous work has found that feelings of satisfaction about an explanation are linked to learning^49^ and those feelings may underpin the drive for information and explanations^50^. In the moral domain, one reason people may want explanations about different agents is because learning about another mind is simply satisfying. Another reason may be that people find this kind of insight useful for their own understanding of the world (though these are not mutually exclusive). Thus, after participants learned about the motives of a selected agent, we asked them to report how satisfied they were with the revealed information and their perceived learning (revealed information judgments). We also tested whether curiosity was associated with actual learning. While previous research has found that curiosity is a better indicator of perceived rather than actual learning^30^, moral information is particularly critical to social cognition^51,52^ and there is evidence that moral status is rapidly learned^53^. As such, we use a memory task at the end of each experiment to assess whether moral curiosity predicted both perceived and actual learning.

Lastly, we examined individual differences that may shed light on the processes that underlie curiosity in the moral domain. Specifically, we included measures of imaginative resistance, perspective-taking ability, morbid curiosity, and need for cognition in an exploratory fashion. When we look to the kinds of stories that make up popular fictional worlds, they tend to have moral rules that closely mirror our own (e.g., loyalty is morally good, betrayal, bad). In contrast, physical laws (e.g., flying on a broomstick) are violated frequently in fictional worlds. This is because imagining worlds where the moral rules are vastly different can engender “imaginative resistance” (i.e., people do not want to imagine a fictional world where it is okay to hurt innocent people or to discriminate against others;^54,55^; see also^56^). We reasoned that people high (vs. low) in imaginative resistance would be less interested in morally bad and ambiguous others. We also reasoned that, in contrast to imaginative resistance, curiosity for both morally bad and ambiguous individuals would be higher for people high in morbid curiosity (the desire to seek out dark or macabre content;^57^). We also included need for cognition to better understand the role that cognitive effort played, given that engaging with ambiguous and even immoral agents might be more effortful. We included perspective-taking ability to test whether ability to think about others’ minds in general played a role in who people chose to learn more about. Finally, we also included a measure of belief in a just world, but unlike we predicted, no significant patterns emerged. We report that measure only in the supplemental material. We only preregistered analyses and not specific hypotheses for the various individual difference measures.

We present Experiments 2a and 2b here together because they are extremely similar. Across both experiments, we preregistered hypotheses and analyses prior to data collection. We note any deviations from the preregistered plan and exploratory analyses as necessary. For brevity, when Experiment 2a results are also representative of 2b, we report on 2a here and 2b in the Supplement and make a note of it in text. We predicted people would show a preference to gain more information about morally bad and morally ambiguous people, which would also be reflected in their self-reported curiosity and satisfaction at the revealed information. We also predicted that expected and perceived learning would be greatest for immoral agents (morally ambiguous and morally bad agents) compared to morally good and average agents.

There are two key differences between the two experiments. In Experiment 2a, participants always choose from all four moral agent types (good, bad, ambiguous, average) while in Experiment 2b, participants choose from only two of the four at a time. This allowed us to investigate whether preferences for moral agents differed when the architecture of the choice set differed. Specifically, previous research suggests that choice sets have an influence on decision-making. People tend to focus on the first thing that they see (primacy effect;^58^), and other times, people select the very last option^59^. In Experiment 2a, participants were presented with four options in fixed positions. To investigate whether our results were an artifact of this set-up and the four-option choice set, Experiment 2b gave people only two-option sets. Participants again saw the same four types of moral agents, but they saw them in two-option sets at a time with agents presented in random order. We also used a different assessment of actual learning in Experiment 2b. We asked participants whether the agent they saw in Phase 3 of each trial was morally good, bad, average, or ambiguous.

Despite the findings from Experiment 2a, we again predicted that moral valence, and specifically immorality, would drive curiosity when the choice set varied. That is, we suspected that when participants were not anchoring on the full range of possible agents, they would be more inclined to select the immoral agent (more closely reflecting the patterns we saw in Study 1). We expected a preference for both morally bad and ambiguous agents to emerge.

## Method

### Design

For Experiment 2a, we used fully within-subjects design, adapted from previous research^48^ to examine the effect of agents’ moral status (morally ambiguous, morally good, morally bad, or morally average) on curiosity. For Experiment 2b, the design was identical to Experiment 2a, but participants only saw two moral agent options on each trial. That is, two options were randomly presented on each trial from the four possible choices (morally ambiguous, morally good, morally bad, or morally average). Participants again completed three phases for each of the 10 trials.

### Participants

#### Experiment 2a

We preregistered recruiting a total of 310 adult (18 and older) participants from Prolific with the goal of preserving a sample size of about 270. We conducted a power analysis using G*Power^60^ to detect an effect of *ω* = 0.2. After excluding people who failed attention and instructions comprehension checks (*N* = 5; pre-registered), we conducted analyses on a final sample of 305 participants (*M*_age_ = 39.92, *SD*_age_ = 13.70, Male = 141, Female = 151, Other = 9). The inclusion criterion for Prolific data collection was current United State residence, no participation in pilot studies related to this experiment, 99% approval rating on Prolific, and a minimum of 5 submissions of Prolific. All participants provided informed consent and were paid $3.04 on average for their participation.

### Experiment 2b

We preregistered doubling the sample size of Experiment 2a. We recruited 665 adult (18 and older) participants from Prolific We used the same preregistered inclusion and exclusion criteria as in Experiment 2a. After excluding people who failed attention and instructions comprehension checks (*N* = 59), we conducted analyses on a final sample of 606 participants (*M*_age_ = 39.71, *SD*_age_ = 13.28, Male = 294, Female = 289, Other = 1). Prolific participants provided informed consent and were paid about $3.00 on average for their participation.

## Materials

### Moral information seeking task

**Figure 1:**
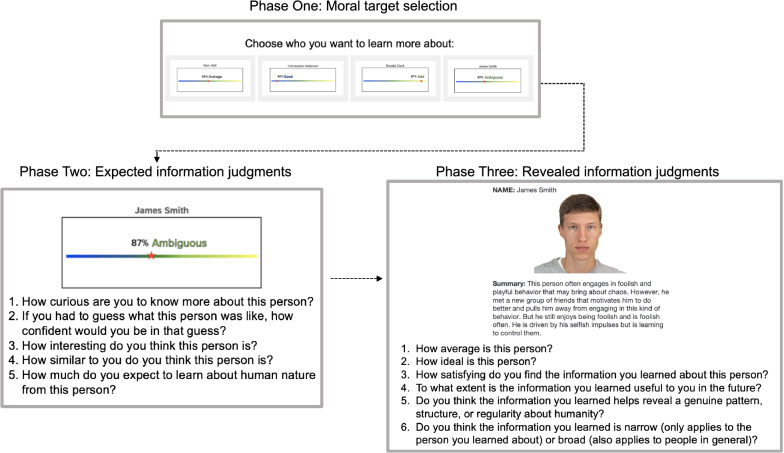
Three phases of Experiments 2a and items rated at each stage. This example depicts the sequence for the selection of an ambiguous agent. Table S2 in the Supplemental Materials lists an example of the revealed information vignettes for each moral agent type. The face pictured is reproduced with permission from the Chicago Face Database.

We used a novel information seeking task adapted from previous work^43^. Participants selected which of the four moral agents (morally good, bad, ambiguous, or average) they would like to learn more about. There were three phases to the task. In Phase 1, participants selected which agent they wanted to learn more about based on their morality (good, bad, average, ambiguous). In Phase 2, participants made judgments (e.g., curiosity, confidence, expected learning) about expected information to provide insight into why people chose a given target. In Phase 3, participants read about the morality and motives of the person they selected, and then they made judgments (e.g., satisfaction, perceived learning) about the revealed information. Each of these phases is described in detail below. See Fig. 1 for a visual depiction of the three phases of a single trial. In Experiment 2b, selected between two moral agents, on each trial, followed by the same Phase 2 and Phase 3 judgments.

### Phase 1: moral agent selection

#### Moral agent stimuli

We selected first and last names from a list of common United States names (e.g., John Williams) to use for our moral agents, which were yoked to a moral character slider manipulation. The sliders and an explicit label indicated the moral status of the agent. Participants were told that a previous sample of participants saw information about these agents and made ratings about their moral status. Specifically, they were told:

In this task, we will ask you to select among four “types” of people. To make your decision, we will show you their name, and a “morality score”. The score is from a previous study that we did. In that study, we had about 70 participants read about some people (with their real names changed to pseudonyms) and rate them in terms of how morally good, bad, ambiguous, and average they are. The morality score you will see for each person is an average calculated from the responses of all the participants in that previous study. Your task is to choose who you would like to learn more about based on their morality score. […] Overall, this task will take the same amount of time regardless of which type of person you choose.

The slider visually depicted those ratings with an accompanying percentage value to indicate agreement for each of the four categories presented on each trial: morally good, morally bad, morally average, and morally ambiguous. Those percentages were matched across trials in their mean and their standard deviation across moral agent types (and did not systemically favor any one moral agent type) and were included to make the task more believable for participants. An example of the slider stimuli is presented in Fig. 1 and the full description of the instructions is available in the Supplemental Materials.

### Phase 2: expected information judgments

#### Curiosity

During Phase 2, participants indicated their curiosity for more information about the selected target. We asked participants, “How curious are you to know more about this person?” rated on a scale from 1 = *Not at all curious* to 7 = *Extremely curious*.

#### Confidence

Participants also indicated their confidence in their prediction about what the selected person will be like. We asked participants “If you had to guess what this person was like, how confident would you be in that guess?” rated on a scale from 1 = *Not at all confident* to 7 = *Extremely confident*.

#### Similarity

Participants answered questions about their similarity (i.e., self-identification with the agent) at the trial level. Participants were asked, “How similar to you do you think this person is?” rated on a scale from 1 = *Not at all like me* to 7 = *Very much like me* to measure similarity.

#### Expected learning about human nature

Participants also indicated whether they believed they would gain knowledge about human nature from learning about the selected target. They were asked, “How much do you expect to learn about human nature from this person?” rated on a scale from 1 = *Nothing* to 7 = *A great deal*.

### Phase 3: Revealed information judgments

#### Revealed moral information

In the final phase of the trial, participants learned about the morality and motives of the person they chose and saw an image of their face. These images were selected from the Chicago Face Database^61^, and matched on attractiveness, age, and dominance^62^. The information revealed was either only extremely good in nature (morally good), extremely bad in nature (morally bad), both good and bad (morally ambiguous), or simplistically moral in nature (morally average). An example of each type of moral agent is reported in the Supplemental Materials (Table S2). We used Linguistic Inquiry and Word Count (LIWC;^63^) to match the revealed information on length and in particular, the Moral Foundation Dictionary in LIWC to examine the descriptive differences on the care dimension for the different categories. We found that bad character vignettes were highest in care vice (most harm language), and the good character vignettes were rated highest in care virtue (most care language). The average and ambiguous explanations fell in the middle. Details are reported in the Supplemental Materials.

#### Normality

Participants rated how “normal” the agents were using average and ideal judgments^64^. We asked participants how average and how ideal they found each of the three Moral Agent Types on a participant-level, rated on a scale from 1 = *Not at all [average/ideal]* to 9 = *Extremely [average/ideal]*.

#### Satisfaction

We also measured whether participants felt a sense of satisfaction after seeing the information about the target. Participants were asked “How satisfying do you find the information you learned about this person?” rated on a scale from 1 = *Not at all* to 7 = *Extremely*.

#### Perceived learning

Finally, we asked participants about how much they explicitly thought they had learned after they read about the person they chose. Specifically, we asked participants about the perceived utility, about the patterns revealed, and whether learning applies narrowly or broadly (adapted from^30^). Each item was measured on a scale from 1 = *Not at all [judgment]* to 7 = *Extremely [judgment]*. Wording for the individual learning items is listed in Fig. 1.

### Individual difference measures

#### Imaginative resistance

We included three representative items from the Imaginative Resistance Scale^65^. A representative item is: “I would be uncomfortable reading a book in which the protagonist thought it was okay to kill people.”, measured on a scale from 1 = *Strongly disagree* to 5 = *Strongly agree*. This exploratory measure was collapsed into a single index (Ex 2a: α = 0.83; Ex 2b: α = 0.83).

#### Morbid curiosity

Six items were selected from the Morbid Curiosity Scale (MSC;^57^). This shortened scale was used to measure the extent to which individuals are attracted to dangerous or unpleasant things. A representative item is: “I am curious about the minds of violent people,” rated from 1 = *Strongly disagree* to 6 = *Strongly agree*. Based on the high internal reliability, a single MSC variable was created by collapsing across the six items (Ex 2a: α = 0.92; Ex 2b: α = 0.94).

#### Need for cognition

Participants completed a shortened form of the Need for Cognition scale (NFC-short;^66^). Items were rated on a scale of 1 = *Extremely uncharacteristic of me* to 5 = *Extremely characteristic of me*. This measure was collapsed into a single index (Ex 2a: α = 0.93; Ex 2b: α = 0.92). Previous research also has found a strong positive correlation between general measures of curiosity and NFC^67^.

#### Perspective taking empathy

We included three representative items from the perspective-taking subscale of the Interpersonal Reactivity Index (IRI;^68^). A representative item is: “I sometimes try to understand my friends better by imagining how things look from their perspective”, which was rated on a scale from 1 = *Does not describe me well to* 5 = *Describes me very well*. This exploratory measure was collapsed into a single index (Ex 2a: α = 0.83; Ex 2b: α = 0.84).

### Exploratory items

In Experiment 2a only, we also asked participants two exploratory questions: “Which kind of person was most fascinating to learn about?” and “Which kind of person was easiest (took least mental or emotional effort) to learn about?” and given the four moral agent types to select one from.

### Actual learning

In Experiment 2a, we assessed actual learning with a memory task for the incidental faces. We showed participants a series of 20 faces that were comprised of faces seen in the 10 trials (regardless of selected Moral Agent Type) and 10 new faces (also from the CFD^61^; the two groups (old vs. new) were matched on attractiveness, age, and dominance). We also tried to select new faces which were visually similar in hair to the target old faces. Participants saw the same face on a trial of the task regardless of agent selection. To examine learning, we asked participants to indicate whether the 20 faces were new or old, meaning that they were faces previously seen in the 10 experimental trials. Given that moral value helps us to amplify what we are visually experiencing^69^, we expected people to remember faces of people they found most interesting and engaging.

In Experiment 2b, we used the same within trial faces, but we changed the measure of what participants learned. Specifically, we showed participant the 10 trial faces they saw, and asked them to recall whether the person was rated as morally good, morally bad, morally average, or morally ambiguous (i.e., 4 AFC), which gave all participants a 25% chance of selecting the correct response. For example, if a participant opted to learn more about a morally good agent in Phase 1, they would later see the same face and be asked to identify their moral status.

### Procedure

After providing informed consent and passing comprehension checks for the instructions, participants answered a series of 10 trials each with three phases (adapted from^48^). In Phase 1 of each trial, participants made a single decision of which of the four Moral Agent Types (average, good, bad, or ambiguous) they were interested in learning more about (all 4 options for Experiment 2a and subset of 2 options for Experiment 2b). After the selection, participants began Phase 2 of the trial which consisted of a series of questions (presented in randomized order) about expected information, and then in Phase 3, they answered questions about the revealed information, including the trial face, normality^64^ of each of the Moral Agent Types, and satisfaction and perceived learning (judgments presented in random order). Following 10 trials, we tested actual learning via the new or old 2-alternative forced-choice (2 AFC; Experiment 2a) or the 4 AFC recall task (Experiment 2b). Participants then completed the individual difference measures (in random order) and demographics questions, and were debriefed and compensated.

## Results

For each experiment, we analyzed the three different phases of the experiment separately. We used a chi-square test to examine information-seeking behavior during the initial moral agent decision. Additionally, we conducted an exploratory time course analysis to examine how agent selection changed over time. Next, we used linear mixed-effects models with participants entered as random effects to compare the effects of Moral Agent Type (good, bad, average, ambiguous) on the Phase 2 expectation judgments (curiosity, expected learning about human nature). We then used linear mixed-effects models with participants entered as a random effect to test whether differences in normality, learning, and satisfaction emerge based on which moral agents that participants selected. As preregistered, we include similarity as a covariate in Phase 2 and Phase 3 analyses.

### Phase 1: moral agent selection

#### Moral information seeking task

**Figure 2:**
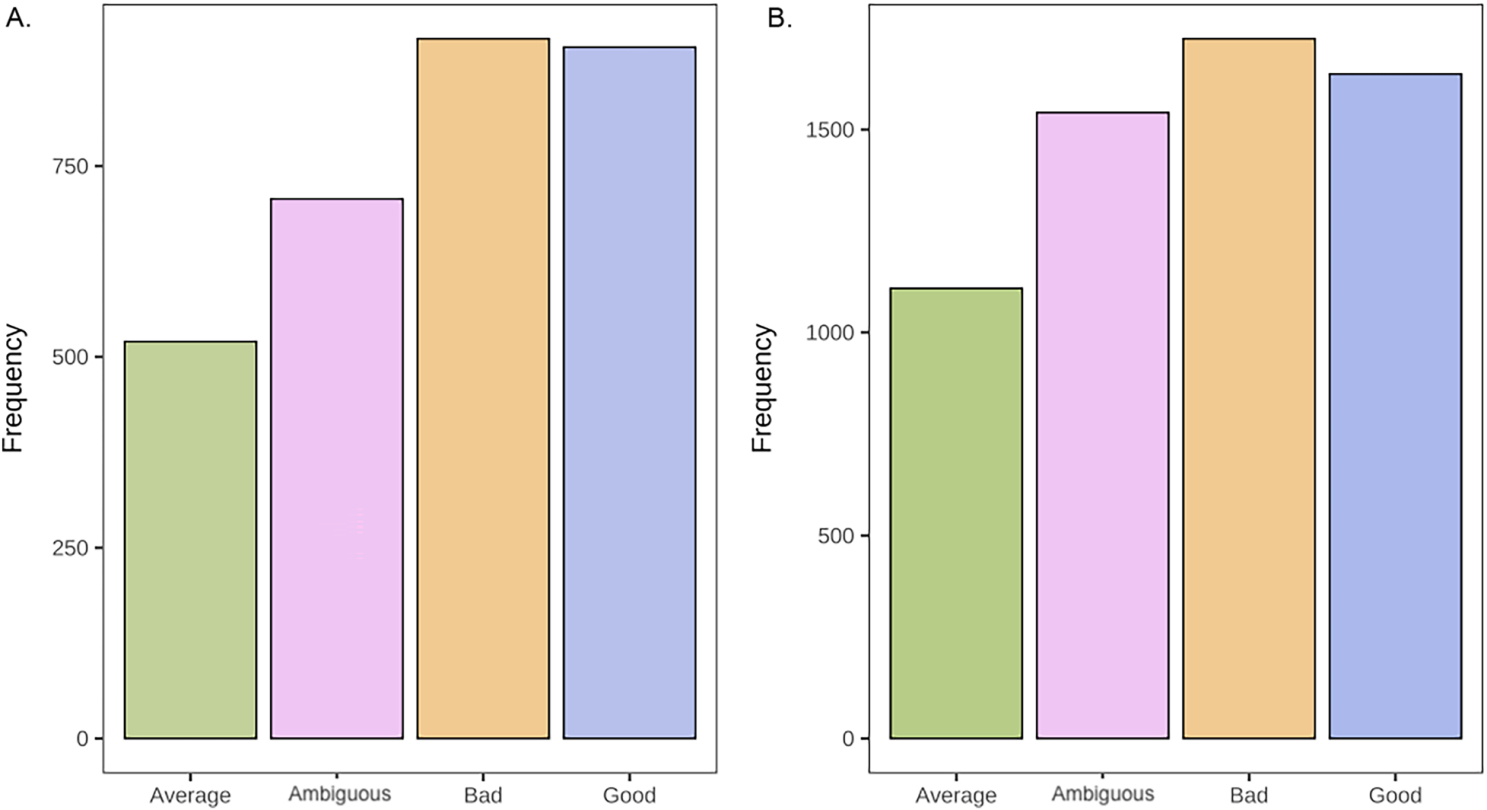
Panel A: Total frequency (counts) for the number of selected Moral Agent Types across participants in Experiments 2a. Panel B: Total frequency for the number of selected Moral Agent Types across participants in Experiment 2b.

To investigate whether participants selected one moral agent to learn about more frequently than the others, we first conducted a chi-square test for given probabilities. In both experiments, the omnibus test suggested that there were statistically significant differences among the choice types, Ex 2a: χ^2^ (3, *N* = 3050) = 139.47, *p* < 0.001, and Ex 2b: χ^2^ (3, *N* = 6012) = 148.74, *p* < 0.001 (see Fig. 2). To investigate pairwise differences between the moral status agents, we used the ‘RVAideMemoire’ package in R^70^. Contrary to predictions, morally good and morally bad agents were selected most frequently. In both experiments, all pairwise comparisons were statistically different from one another (*p*’s < 0.001) besides morally good compared to morally bad (*p* = 0.99) in 2a. In Experiment 2b, morally bad was also statistically different from morally ambiguous (*p* = 0.009).

For Experiment 2a, we also conducted exploratory analyses on preferences over the course of the task. We first tested whether the patterns looked similar for the first trial of the task as they do for the task overall. This is a particularly strong test of the hypothesis as it allows us to investigate which agents people are most curious about before gaining access to any additional information about the different agents. The patterns of results did indeed match the patterns looking across the task, χ^2^ (3, *N* = 305) = 93.28, *p* < 0.001. People more frequently selected an immoral agent on the first trial compared to all other possible agents (*p*_*good*_: = 0.039; *p*_*ambig*_: < 0.001; *p*_*avg*_ < 0.001). We also examined whether previous Phase 1 selection influenced target variables in Phase 2 (curiosity) and Phase 3 (perceived learning). We found no statistically significant effects of previous trial Phase 1 selection on these variables. These additional analyses, and time course analysis for 2b, are reported in the Supplemental Materials.

### Phase 2: expected information judgments

For the Phase 2 analyses, all models were specified following the same format. We specified by-participant random intercepts and slopes, included similarity as a covariate (though patterns of results remain unchanged when excluding them; see Model Comparisons in the Supplemental Material), and Phase 1 decision was included as a fixed effect. All models use morally average as the comparison group.

### Curiosity and confidence

**Figure 3:**
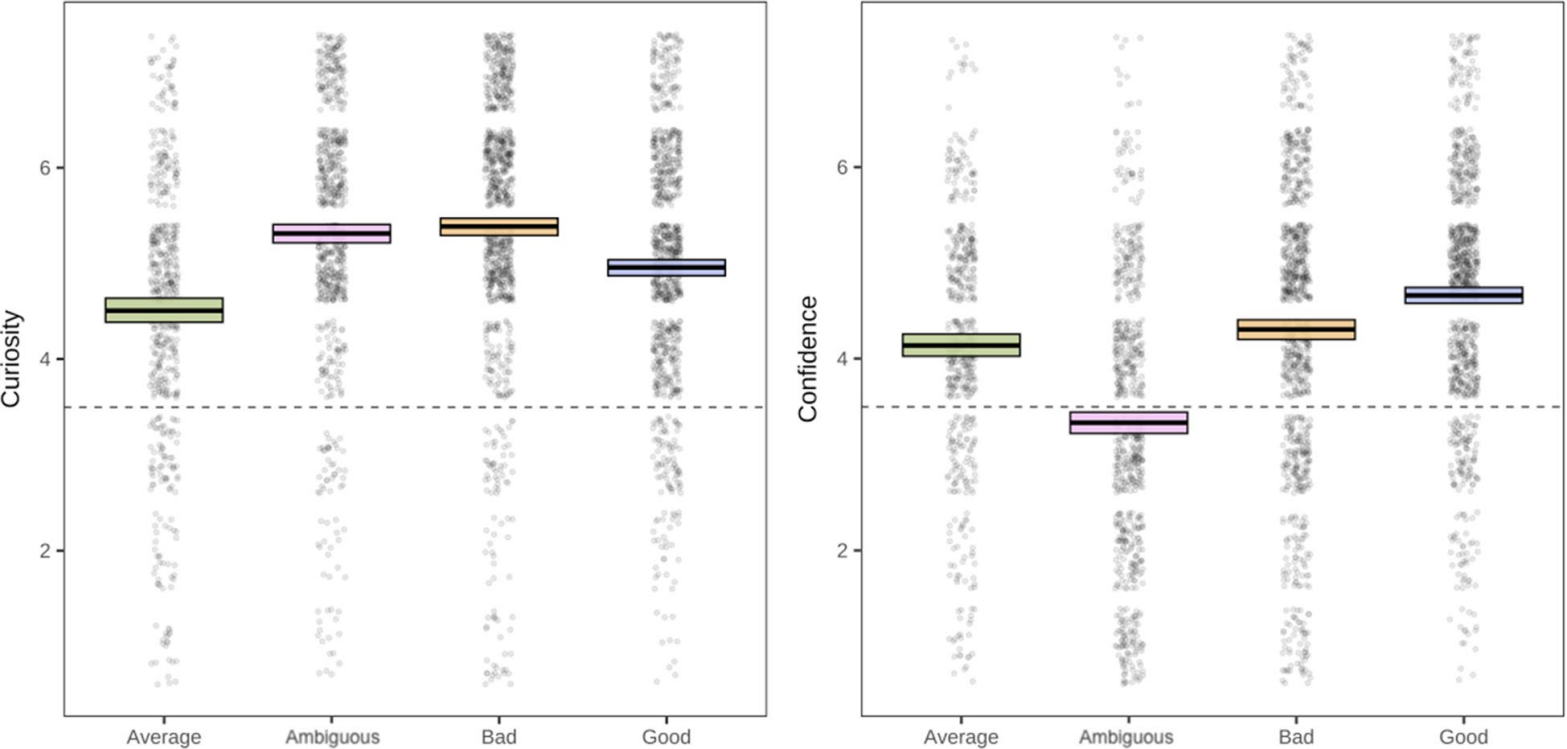
Patterns of results for the curiosity and confidence ratings. The thick black line in the boxes represents the mean, and the edges represent mean standard error from bootstrap.

First, we tested whether participants reported different levels of curiosity for the expected information about the moral agent they selected, and confidence about what they would learn about them. Despite our expectation that people are more curious for information when they are less confident about that information (i.e., want to close the information-gap;^71^), confidence and curiosity were moderately positively corrected, Experiment 2a: *r* = 0.22, *p* < 0.001; Experiment 2b *r* = 0.25, *p* < 0.001 (Holm corrections). Looking to curiosity, results from Experiment 2a suggested that participants were more curious about ambiguous (*b* = 0.89, *SE* = 0.08, *t*(282) = 11.31, *p* < 0.001, *r* = 0.56, *95% CI* [0.74, 1.05]), bad (*b* = 1.24, *SE* = 0.09, *t*(386) = 13.49, *p* < 0.001, *r* = 0.57, *95% CI* [1.06, 1.43]), and good (*b* = 0.42, *SE* = 0.02, *t*(252) = 6.75, *p* < 0.001, *r* = 0.39, *95% CI* [0.30, 0.54]) moral agents than average ones. Experiment 2b found the same pattern of results, which is reported in the Supplement. We also measured and analyzed self-reported interest; patterns mirror curiosity for both Experiment 2a and 2b and it is reported in the Supplemental Material as well. Overall, in both experiments, participants reported the most curiosity for the ambiguous and the bad moral agents (see Fig. 3).

For the confidence judgments, Experiment 2a was fit with by-participant random slopes only (model was singular with more complex random effect structure). Participants reported significantly less confidence for ambiguous agents (*b* =  − 0.61, *SE* = 0.07, *t*(2813) =  − 9.27, *p* < 0.001, *r* = 0.17, *95% CI* [−0.74, −0.48]) than average agents. For the two morally extreme categories—good and bad—participants reported more confidence. Compared to average, bad agents (*b* = 0.58, *SE* = 0.08, *t*(2868) = 7.55, *p* < 0.001, *r* = 0.14, *95% CI* [0.43, 0.73]), and good ones (*b* = 0.50, *SE* = 0.06, *t*(2831) = 8.05, *p* < 0.001, *r* = 0.15, *95% CI* [0.38, 0.62]) induced the most feelings of confidence (see Fig. 3). Confidence was lowest for the ambiguous agents compared to all other agents in both experiments, situating moral ambiguity as uniquely mysterious. People were both relatively curious about these agents and unsure of what they would learn about them (results for 2b reported in the Supplement and include random slopes).

Given the correlated nature of confidence and curiosity, we also conducted additional exploratory analyses that control for confidence when predicting curiosity for both Experiment 2a and 2b. We control for confidence here rather than curiosity because curiosity is central to our theorizing, but these analyses should be interpreted with caution because they are exploratory and because their causal structure is not clear^72^. When we fit this simultaneous regression with the Phase 1 decision and all Phase 2 predictors, we find that the effect of moral agent type selected in Phase 1 retained statistical significance in predicting curiosity judgments during Phase 2 (Table 2). When we included each of the Phase 2 judgments and the interaction term between confidence and moral agent selection in an exploratory analysis, we find that people report more curiosity for morally ambiguous and morally bad agents but not morally good ones relative to average agents. This suggests that curiosity and confidence are not uniform across moral agent types and relate to selection differentially. For example, people are most curious about ambiguous agents when they are unsure what they will learn. Full model results are reported in the Supplemental Materials. We find that when controlling for confidence, expected learning about human nature, and similarity, judgments of curiosity are still greater for morally ambiguous and morally bad agents compared to morally average agents. When controlling for these same variables, we also find that morally ambiguous (2a: b = 0.57, SE = 0.07, *t*(329) = 8.27,* p* < 0.001*;* 2b: *b* = 0.28, *SE* = 0.05, *t*(733) = 5.63, *p* < 0.001) and morally bad (2a: *b* = 0.73, *SE* = 0.07, *t*(494) = 9.78, *p* < 0.001; 2b: *b* = 0.69, *SE* = 0.05, *t*(1111) = 12.55, *p* < 0.001) agents elicited more curiosity than morally good agents. See Table 2 for full results for Experiment 2a and 2b. Greater curiosity for morally ambiguous and bad agents over good and average ones was not explained by confidence, similarity, or learning about human nature alone.

**Table 2:** Estimates for regression models predicting curiosity in Experiment 2a and 2b.

Curiosity						
Variable	Experiment 2a			Experiment 2b		
	Beta	95% CI^1^	*p* value	beta	95% CI^1^	*p* value
**Moral Agent Selection**			** < 0.001**			** < 0.001**
*Average (reference group)*	–	–		–	–	
*Ambiguous*	0.88	0.73, 1.0		0.68	0.59, 0.78	
*Bad*	1.0	0.88, 1.2		1.1	0.98, 1.2	
*Good*	0.31	0.19, 0.44		0.40	0.31, 0.49	
**Confidence**	0.07	0.04, 0.10	** < 0.001**	0.05	0.03, 0.08	** < 0.001**
**Expected Learning**	0.29	0.26, 0.32	** < 0.001**	0.37	0.35, 0.40	** < 0.001**
**Human Nature**						
**Similarity**	0.06	0.03, 0.09	** < 0.001**	0.04	0.02, 0.07	** < 0.001**

^1^CI = Confidence Interval.

Significant values are in bold.

### Expected learning about human nature

We also asked participants to indicate whether they expected to learn something about human nature from the selected target. Results yielded similar patterns to curiosity. Participants reported more expected learning about human nature for ambiguous (*b* = 0.19, *SE* = 0.07, *t*(268) = 2.66,* p* = 0.008, *r* = 0.16, *95% CI* [0.05, 0.33]), bad (*b* = 0.57, *SE* = 0.09, *t*(406) = 6.34, *p* < 0.001, *r* = 0.30, *95% CI* [0.39, 0.75]), and good (*b* = 0.25, *SE* = 0.07, *t*(231) = 3.79, *p* < 0.001, *r* = 0.24, *95% CI* [0.12, 0.38]) compared to average moral agents. When excluding similarity as a covariate, ambiguous agents are no longer statistically different from average ones *(b* = 0.06, *SE* = 0.07, *t*(256) = 0.85, *p* = 0.397, *r* = 0.05) in Experiment 2a, but ambiguous agents retain statistical significance (relative to average agents) in Experiment 2b, *b* = 0.12, *SE* = 0.05, *t*(485) = 2.45, *p* = 0.015, *r* = 0.11. Expected learning about human nature was greater for the morally bad, good, and ambiguous agents compared to morally average despite morally average people being the ones you might be most likely to meet. These patterns were again consistent in Experiment 2b (*p*’s < 0.001; see Supplemental Materials).

### Phase 3: revealed information judgments

In Phase 3, we preregistered using a similar specification as used in Phase 2, testing whether Phase 1 moral character selection predicted Phase 3 revealed information judgments. We also explored whether Phase 2 curiosity judgments predicted Phase 3 revealed information judgments for the different moral agents. All of the following models include by-participant random slopes and intercepts unless otherwise stated.

### Normality judgments

As a manipulation check, we explored how normality ratings (comprising both average and ideal judgments;^64^) were predicted by moral agent selection. Results are consistent for Experiments 2a and 2b (see Supplement for 2b results) for both average and ideal judgments. For Experiment 2a, findings suggested that ambiguous moral agents, *b* =  − 1.58, *SE* = 0.09, t(284) =  − 17.32, *p* < 0.001,* r* = 0.72, *95% CI* [−1.76, -−1.40], morally bad agents, *b* =  − 2.44, *SE* = 0.10, *t*(458) =  − 23.58, *p* < 0.001, r = 0.74, *95% CI* [−2.65, −2.24], and morally good agents,* b* = –1.94, SE = 0.09, t(276) =  − 22.31, *p* < 0.001, r = 0.80, *95% CI* [−2.11, −1.77], were all rated as less average than average moral agents. Interestingly, only ambiguous (*M* = 3.59, *SD* = 1.42) and average (*M* = 5.24, *SD* = 1.29) moral agents had mean ratings above the mid-point on the scale, suggesting that both morally average and morally ambiguous people were seen as typical agents that people are likely to encounter. Curiosity and the interaction between curiosity and Phase 1 target selection did not significantly predict average judgments, *p*’s > 0.18. These patterns were consistent in Experiment 2b (see Supplemental Materials).

**Figure 4:**
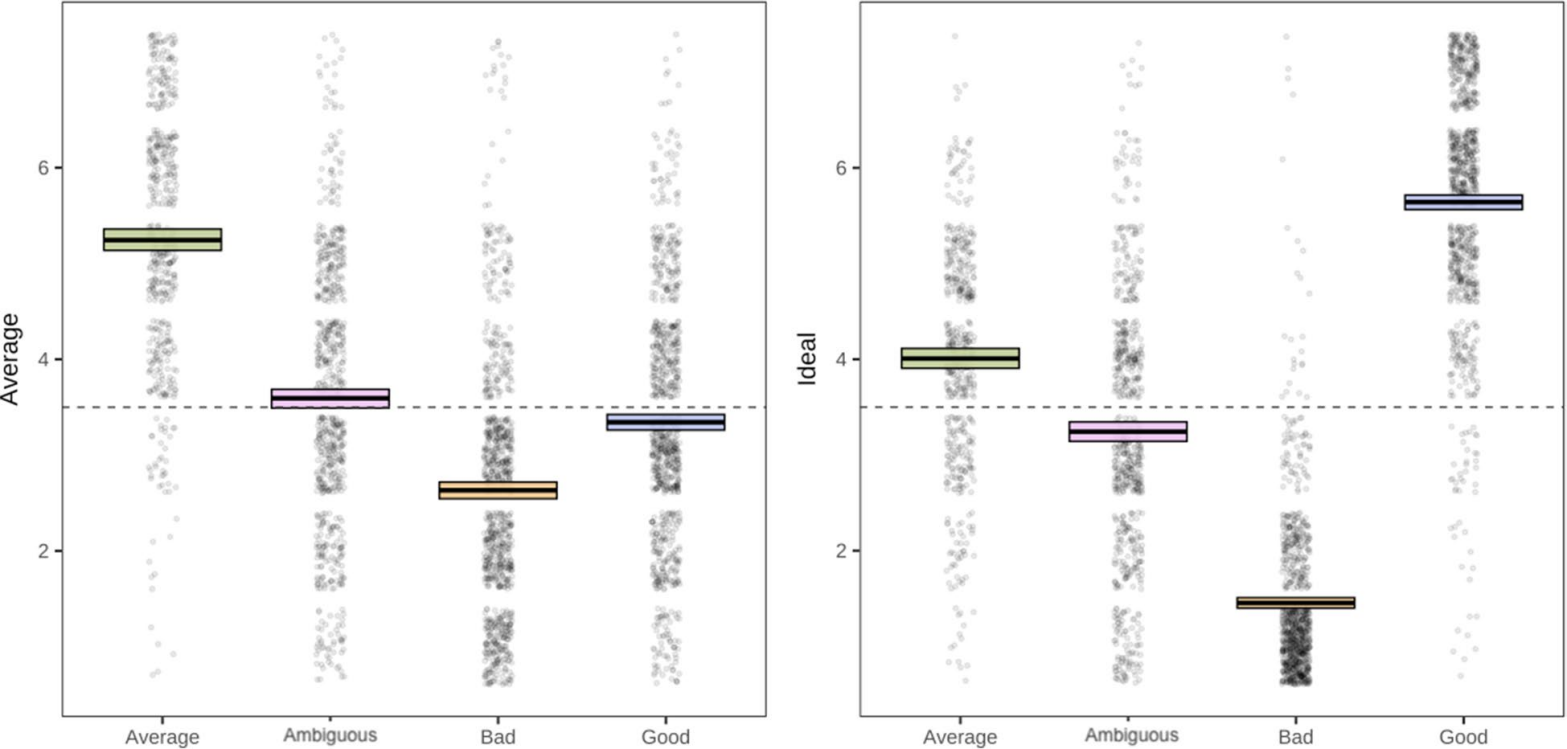
Patterns of results for the average and ideal ratings in Experiment 2a. The thick black line in the boxes represents the mean, and the edges represent mean standard error from bootstrap.

As expected, for ideal judgments in Experiment 2a, participants rated morally ambiguous, *b* =  − 0.60, *SE* = 0.08, *t*(252) =  − 7.99, *p* < 0.001, *r* = 0.45, *95% CI* [−0.75,−0.45], and morally bad agents, *b* =  − 2.26, *SE* = 0.08, *t*(456) =  − 27.68, *p* < 0.001, *r* = 0.79, *95% CI* [−2.42, −2.10], as less ideal than morally average. But that morally good agents were more ideal than morally average, *b* = 1.71, *SE* = 0.08, *t*(283) = 21.74, *p* < 0.001, *r* = 0.79, *95% CI* [1.55, 1.86] (see Fig. 4). Average (*M* = 4.01, *SD* = 1.19) and good (*M* = 5.64, *SD* = 1.20) moral agents were rated above mid-point on the scale, suggesting people think average and good people are both more good than they are bad. Findings were consistent in Experiment 2b. Curiosity and the interaction between curiosity and Phase 1 target selection were again not statistically significant predictors, *p*’s > 0.32 (see Supplement for Experiment 2b). Overall, the normality judgments confirmed that our manipulations were successful: the morally average agent was seen as most average, and the absolute value of the ideal judgments for the morally good and morally bad agents were similar. The ideal judgments for the morally good and bad were most extreme. Altogether, people found the good and bad people to be extreme and counter-normative, the average and morally ambiguous people to be more common, and the good and average people to be good.

### Satisfaction

We tested whether moral agent selection at Phase 1 affected satisfaction at Phase 3 without including curiosity as a predictor in an exploratory manner. For both Experiments 2a and 2b, results suggested that satisfaction of the revealed explanation during the third phase of the trial was higher for the ambiguous, *b* = 0.33, *SE* = 0.09, *t*(292) = 3.78, *p* < 0.001, *r* = 0.22, *95% CI* [0.16, 0.51], and the good agents, *b* = 0.92, *SE* = 0.09, *t*(272) = 9.77, *p* < 0.001, *r* = 0.51, *95% CI* [0.74, 1.11], compared to the average agents (see Supplement for 2b). Contrary to our expectations, satisfaction was lower for the bad moral agents than the average agents, *b* =  − 0.31, *SE* = 0.11, *t*(464) =  − 2.82, *p* = 0.005, *r* = 0.13, *95% CI* [−0.53, −0.09] though only marginally so for Experiment 2b, *b* =  − 0.15, SE = 0.08, *t*(870) =  − 1.96, *p* = 0.050, *r* = 0.07, *95% CI* [−0.31, 0.001]. Overall, satisfaction was greatest for the ambiguous and good moral agents. We also preregistered investigating whether curiosity predicted satisfaction, and we explored whether the type of moral agent moderated that relationship. When including curiosity and the interaction term, the patterns of results for Phase 1 decision remained consistent, though the marginal effect for bad agents and ambiguous agents were no longer statistically significant, *p*’s < 0.17. Critically, curiosity was a significant predictor of satisfaction in both Experiment 2a (*b* = 0.27, *SE* = 0.04, *t*(465) = 6.27, *p* < 0.001, *r* = 0.28, *95% CI* [0.18, 0.36]) and Experiment 2b (*b* = 0.27, *SE* = 0.03, *t*(1173) = 10.80, *p* < 0.001, *r* = 0.30, *95% CI* [0.24, 0.30]). There were no significant interaction effects in either experiment.

### Perceived learning

We also sought to investigate what kind of learning people expected to gain from moral agents. It is possible that one type of target (e.g., bad) would provide the most learning overall, or that different agents provide different opportunities for learning. Accordingly, we first investigated whether the perceived learning questions were related. The correlations among the measures were moderate, *r*’s < 0.48 and the internal reliability low (Cronbach’s alpha = 0.62). As such, we analyzed each of the learning items separately. For each of the perceived learning items, we used only one model specification: we entered curiosity, Phase 1 moral agent selection, and their interaction term as fixed effects, including random intercept and slopes for participant for each learning item.

### Learning utility

For perceived utility of learning in Experiment 2a, we asked whether people believed they learned information that was useful for the future. The overall effect of curiosity was statistically significant, *b* = 0.18, *SE* = 0.04, *t*(565) = 4.51, *p* < 0.001, *r* = 0.19, *95% CI* [0.10, 0.27], such that more curiosity predicted higher ratings of learning utility. There was no significant interaction or marginal main effect of Phase 1 target selection. The more individuals reported being curious, the more they reported learning something useful regardless of which moral agent they chose to learn more about.

In Experiment 2b, Curiosity again predicted perceived utility of learning, *b* = 0.27, SE = 0.03, t(1173) = 10.80, *p* < 0.001, r = 0.30, *95% CI* [0.22, 0.33], such that more curiosity predicted more learning utility in Phase Three. The relationship between curiosity and learning utility was moderated by moral target. Morally good agents did not differ from morally average in magnitude of relation, *p* = 0.895, but both morally ambiguous moral agents, *b* =  − 0.09, SE = 0.03, t(1318) =  − 2.86, *p* = 0.004, r = 0.08, *95% CI* [−0.16, −0.03], and bad ones, *b* =  − 0.11, SE = 0.04, t(2169) =  − 2.98, *p* = 0.003, r = 0.06, *95% CI* [−0.18, −0.03], showed an attenuated relationship between curiosity and perceived learning—people may feel they are curious about bad and ambiguous agents for reasons other than learning, or the question about learning may suggest learning what to do (and not what not to do).

### Revealing a pattern

For the judgment that Phase 3 information reveals a real-life pattern in Experiment 2a, results suggested that ambiguous, *b* =  − 0.90, *SE* = 0.28, t(674) =  − 3.18, *p* = 0.002, r = 0.12, *95% CI* [−1.46, −0.33], good, *b* =  − 0.96, *SE* = 0.28, t(957) =  − 3.42, *p* < 0.001, r = 0.11, *95% CI* [−1.59, −0.43], and bad, *b* =  − 1.01, *SE* = 0.29, t(1026) =  − 3.44, *p* < 0.001, r = 0.11, *95% CI* [−1.53, −0.40] moral agents were each less likely to reveal a real-life pattern than average moral agents. There was no overall effect of curiosity alone (*b* = 0.05, *p* = 0.21). The interaction between moral curiosity and morally good target selection predicting perceiving a pattern was statistically significant, *b* = 0.12, *SE* = 0.06, *t*(1043) = 2.10, *p* = 0.036, *r* = 0.06, *95% CI* [0.01, 0.23], relative to average moral agents. There were no other significant interactions. Participants rated the average agents as most informative of patterns encountered in real life despite opting to learn more about them the least often. In Experiment 2b, curiosity again predicted learning, *b* = 0.17, *SE* = 0.03, *t*(1306) = 5.80, *p* < 0.001,* r* = 0.16, [0.11, 0.23] but there were no significant interactions between curiosity and moral agent.

### Broad versus. narrow learning

**Figure 5:**
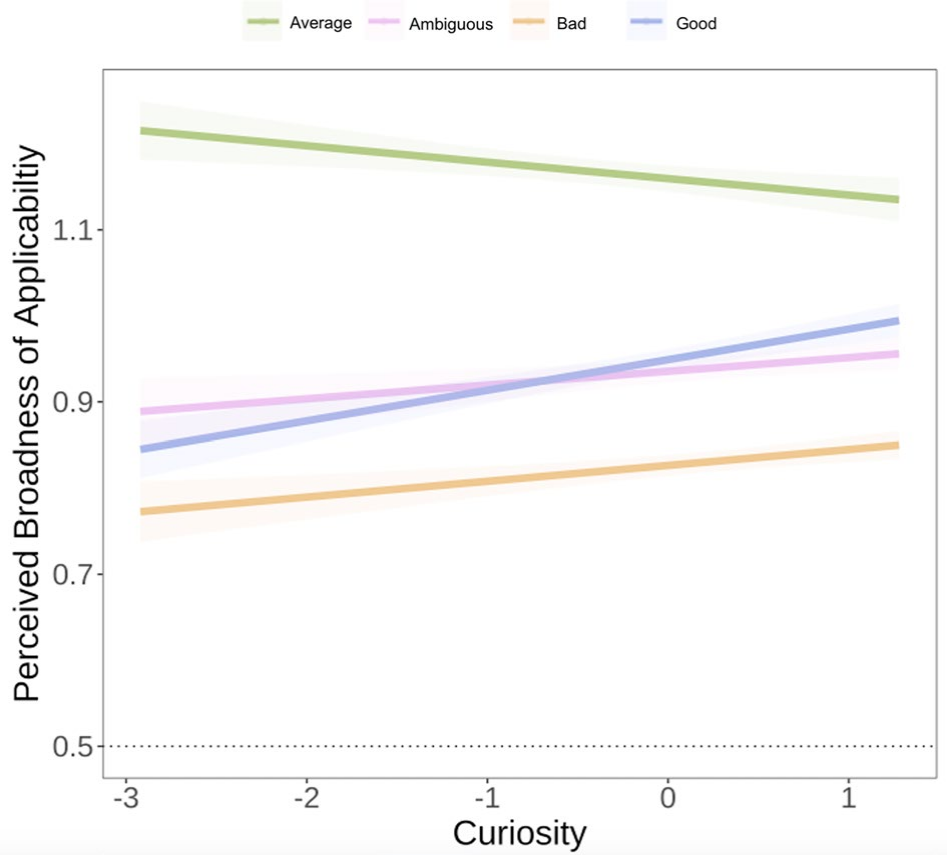
Interaction between curiosity (scaled) and Phase 1 moral agent selection. Confidence intervals represent ± 1 SE. Model is fit with participants included as random intercepts.

Lastly, we investigated whether curiosity predicted learning about something broad (vs. something narrow and only related to the present experiment). Perceptions that Phase 3 revealed broadly applicable knowledge in Experiment 2a showed similar patterns of effects as the pattern-related learning question. Again, ambiguous, *b* =  − 1.80, *SE* = 0.32,* t*(684) =  − 5.65, *p* < 0.001, *r* = 0.21, *95% CI* [−2.43, −1.17], good, *b* =  − 1.89, *SE* = 0.30, *t*(806) =  − 6.23, *p* < 0.001, *r* = 0.21, *95% CI* [−2.49, −1.29], and also bad, *b* =  − 2.60, SE = 0.33, t(970) =  − 7.99, *p* < 0.001, r = 0.25, *95% CI* [3.24, −1.95], moral agents were rated as lower in their broad applicability compared to average moral agents. There was also no overall effect of curiosity alone (*b* = −0.07, *p* = 0.16). However, significant interactions again emerged. For morally ambiguous agents, increases in curiosity predicted broader applicability, *b* = 0.13, *SE* = 0.05, *t*(741) = 2.04, *p* = 0.042, *r* = 0.07, *95% CI* [0.004, 0.25]. The same was true for morally good agents, *b* = 0.15, *SE* = 0.06, *t*(861) = 2.47,* p* = 0.014, *r* = 0.08, *95% CI* [0.03, 0.28], and morally bad agents, *b* = 0.18, *SE* = 0.06, *t*(1022) = 2.78, *p* = 0.005,* r* = 0.09, *95% CI* [0.05, 0.30]. Overall, relative to morally average (which showed a negative slope but was not different from 0; simple slope:* b* = -0.07, *SE* = 0.05, *t* = -1.41, *p* = 0.16), as curiosity increased so too did perceived broadness of learning (see Fig. 5). In Experiment 2b, neither curiosity, nor interactions between curiosity and moral agent, predicted broadness of learning.

### Individual differences

Given that the dispositional measures had different scales, we first z-scored the individual difference traits prior to analysis. Next, we examined the correlational structure of the individual difference measures for Experiment 2a and report them in Table 3.

**Table 3:** Correlation matrix for individual difference measures in Experiment 2a, see Supplement for 2b.

Measure	1	2	3	4	5
1. Morbid curiosity	–				
2. Need for cognition	0.02	–			
3. Belief in a Just world	0.03	0.19**	–		
4. Imaginative resistance	− 0.30***	− 0.02	0.11	–	
5. Perspective-taking empathy	0.18*	0.32***	0.08	0.07	–

N = 305. *p < .05, **p < .01, ***p < .001. Holm method for *p*-value adjustment.

We tested whether the individual differences moderated the relationship between Phase 1 moral agent selection and curiosity. For each of the dispositions measured, people on the high end of the scales reported more curiosity for the moral agents. There were significant interactions for each of the models as well. Here, we report the results for the imaginative resistance model because it was the only one where the interaction was not simply a magnitude effect (e.g., people high in morbid curiosity were more curious about bad moral agents). The other individual differences are reported in Supplemental Materials.

**Figure 6:**
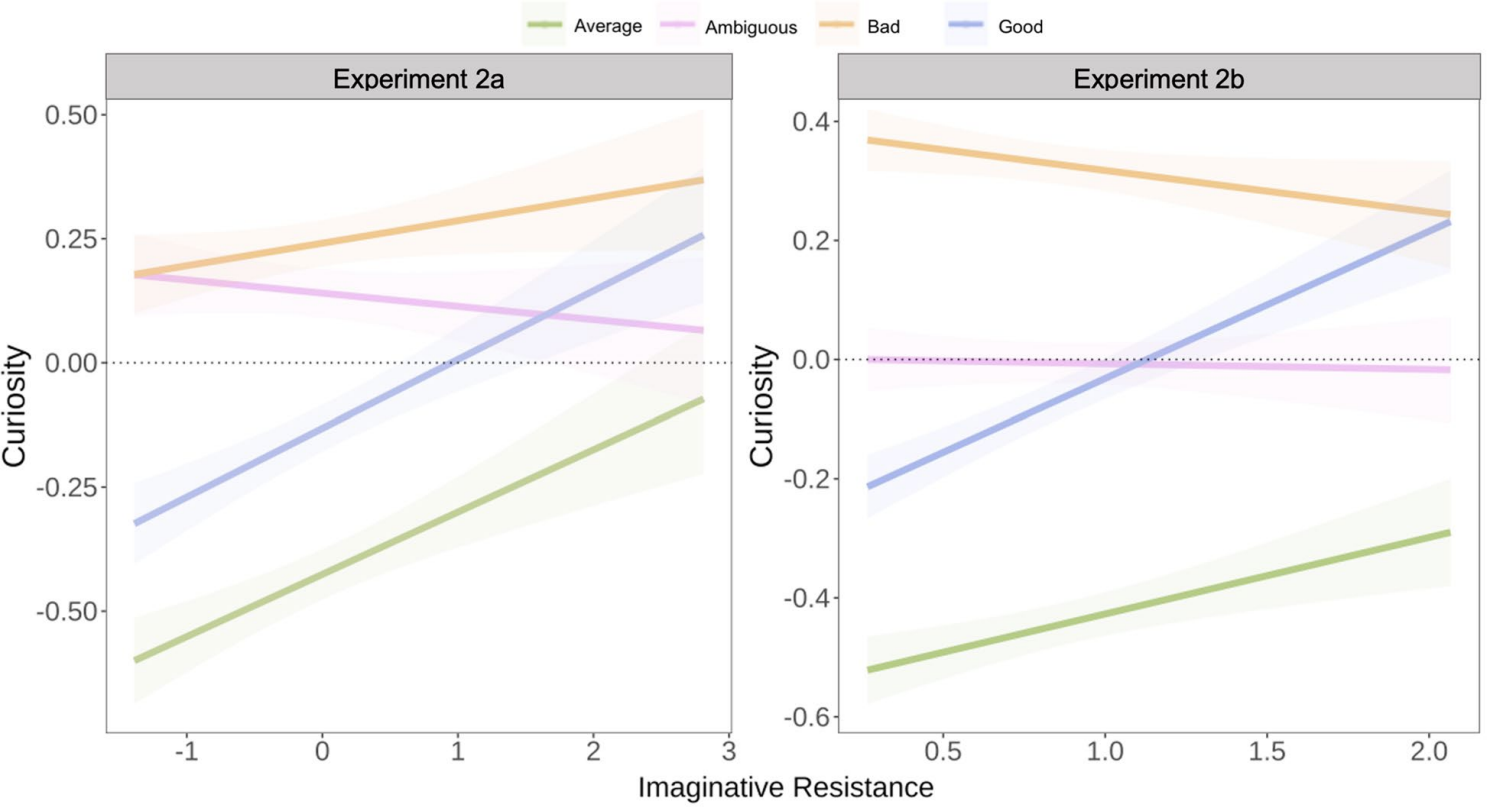
Interaction between imaginative resistance (scaled) and Phase 1 moral agent selection in Experiments 2a. Confidence intervals represent ± 1 SE. Model is fit with participants included as random intercepts. Experiment 2a is depicted in the left panel, 2b in the right.

When including Phase 1 moral agent selection as a fixed effect, random slopes and intercepts for participants, and the interaction term, the marginal effect of imaginative resistance was statistically significant, *b* = 0.16, *SE* = 0.08, *t(*271) = 2.05, *p* = 0.042, *r* = 0.12, *95% CI* [0.01, 0.32] (see Fig. 6), and the effects of morally ambiguous (*b* = 0.80, *SE* = 0.08, *t*(262) = 10.44, *p* < 0.001, *r* = 0.5, *95% CI* [0.65, 0.95]), morally bad (*b* = 0.96, *SE* = 0.08, *t*(256) = 11.94, *p* < 0.001, *r* = 0.60, *95% CI* [0.81, 1.12]), and morally good (*b* = 0.41, *SE* = 0.06, *t*(251) = 6.55, *p* < 0.001, *r* = 0.38, *95% CI* [0.29, 0.53]) agents relative to morally average retained  statistical significance. However, there was a significant interaction between morally ambiguous agents and imaginative resistance relative to morally average agents, *b* =  − 0.20, *SE* = 0.08, *t*(276) =  − 2.58, *p* = 0.010, *r* = 0.15, *95% CI* [−0.35, −0.04]. Simple slopes analysis suggested that the slopes for average and the good moral agents were statistically significant, average: *b* = 0.16, *SE* = 0.08, *t* = 2.05, *p* = 0.04; good: *b* = 0.20, *SE* = 0.07, *t* = 3.02, *p* < 0.001, but the slopes for morally ambiguous or morally bad individuals were not (morally ambiguous*: b* = −0.04, *SE* = 0.07, *t* = −0.55, *p* = 0.58; morally bad: *b* = 0.05, *SE* = 0.07, *t* = 0.66, *p* = 0.51). People willing to engage in the idea of alternate moral possibilities (i.e., those low in imaginative resistance) were less likely to be curious about the morally good and average others, while those who do not want to engage with alternate moral worlds were curious the morally good and average agents. Patterns are again similar for Experiment 2b with one exception: Unlike in Experiment 2a, the interaction between curiosity for morally bad agents and imaginative resistance was statistically different from that of average agents, *b* =  − 0.15, *SE* = 0.06, *t*(542) =  − 2.75, *p* = 0.006, *r* = 0.12, *95% CI* [−0.14, −0.02] (see Fig. 6). While the simple slope for bad agents was not significant (*b* = −0.06, *SE* = 0.05, *t* = −1.19, *p* = 0.24), this difference between imaginative resistance for morally bad agents and average ones suggests that people who are more resistant to imagining moral deviance were less curious for information about the morally bad agents. When the choice set was restricted to two options, the advantage in curiosity that morally bad agents have was attenuated for those high in imaginative resistance. The other individual differences and full statistics are reported in the Supplemental Materials.

### Actual learning

We examined whether moral curiosity influenced actual learning of incidental target faces in an exploratory manner. To do this, we created an average score for the memory task in both 2a (*M* = 0.75, *SD* = 0.13) and 2b (*M* = 0.30, *SD* = 0.15), and we tested whether collapsed curiosity ratings or predicted utility of learning ratings predicted this overall memory task performance.

### Experiment 2a

Results did not support our predictions. Moral curiosity did not predict later memory performance,* p* = 0.41. Patterns of results remain unchanged when investigating these ratings for the different Phase 1 selected moral agents, and moral agent type alone did not predict memory performance. In contrast, perceived utility of learning did emerge as a small but statistically significant predictor of memory performance,* b* = 0.01, *SE* = 0.006, *t(*303) = 2.25, *p* = 0.025, *r* = 0.13, *95% CI* [0.002, 0.03].

### Experiment 2b

Results again did not support our predictions: Curiosity was not a significant predictor of actual learning, *p* = 0.573. However, perceived utility of learning again significantly predicted actual accuracy, *b* =  − 0.01, *SE* = 0.005, *t*(593) =  − 2.32, *p* = 0.021, *r* =  − 0.10, *95% CI* [-0.02, -0.002], but in the opposite direction as expected. The more people perceived there to be utility to what they learned, the less accurate their actual memory performance.

We also explored whether accuracy was better for the immoral compared to morally good or average phase one selections. Relative to morally average, results suggested that actual memory was best when people selected the morally bad agent in phase one, *b* = 0.32, *SE* = 0.02, *t*(2242) = 17.26, *p* < 0.001, *r* = 0.34. Memory performance for the morally good and morally ambiguous selections was slightly worse than for morally average (Good: *b* =  − 0.05, *SE* = 0.02, *t*(2242) =  − 2.65, *p* = 0.008, *r* =  − 0.06; Ambiguous:* b* =  − 0.07, *SE* = 0.02, *t*(2242) =  − 3.91, *p* < 0.001, *r* =  − 0.08). These patterns remain consistent (though were attenuated) when excluding those with memory performance below 25% (chance). Overall, participants actually learned more about the morally bad agents, despite reporting curiosity for bad, good, and ambiguous agents alike.

### Exploratory measures

We also included two exploratory measures of ease and fascination in Experiment 2a. Overall, 40% participants reported that the ambiguous moral agents were most “fascinating” (vs. 31% for bad, 23% for good, and 6% for average moral agents), and 58% reported that the good moral agents were “easiest” (vs. 21% for average, 16% for bad, and 4% for ambiguous moral agents).

## Discussion

Moral agents who are morally atypical—both the morally bad and morally good ones—were more likely to be selected for further learning than both the morally ambiguous and morally average. We also found that morally ambiguous agents were selected more than the morally average agents. When the choice set included all four possible options simultaneously, participants were more eager to learn about the moral agents who were least average and whose ideal judgments were most extreme. We found little variation in preferences between the differing choice sets (4 vs. 2), besides a minor (but not statistically significant) increase in the total number of immoral agents (the bad and the ambiguous agents individually) selected relative to the number of good agents selected. We think this raises the possibility that people are less susceptible to the influence of choice architecture on decision-making when the choices are in the moral domain.

Interestingly, the curiosity and confidence judgments painted a slightly different picture. People were least confident about what they would learn about morally ambiguous agents (making them the most mysterious), curious about all moral agents more than the average moral agent, and most likely to seek out more information about morally extreme agents. We also examined some of the functions of moral curiosity. Matching previous work^30^, we found that curiosity was a better indicator of expectations and perceptions about learning rather than actual learning. Interestingly, we also found that memory was better for the bad moral agents than any other group in Experiment 2b. This suggests that while epistemic motives may drive preferences, the value of the information encountered had a stronger effect on memory than the motives that engendered the action. This pattern also fits well with literature on moral learning which suggests people quickly learn about morally bad others, despite being more uncertain about their behavior^23^. It is also interesting that confidence and curiosity were positively correlated, which we did not expect [see e.g., 7]. People are sometimes most curious to learn about things they think they know but aren’t completely sure about or can’t access^73,74^—a desire which may be particularly potent in the moral domain. People are motivated to maintain coherent views of their moral worlds^19^. Gaining access to information that confirms what one believes about what constitutes good and evil may be one way to uphold and maintain a coherent moral worldview. Ambiguous moral agents also appear to be uniquely mysterious, participants tend to say they pique their curiosity and that they are relatively unsure what they will learn about them.

We also found that people expect to learn distinct things from different moral agents. While people say that the ambiguous, bad, and good agents can tell them more about human nature than the average agents, average agents were most likely to reveal a real-life pattern and have broader applicability. In other words, participants recognize that average people can in some sense tell them something useful about everyday life because they are most likely to encounter them. Yet, they are not the moral agents that people opt to learn more about.

Finally, we also investigated whether individual differences moderated moral curiosity. Interestingly, we found that people high in imaginative resistance were less curious about morally ambiguous agents than those low in imaginative resistance. We take this as evidence of the unique space that moral ambiguity exists in. Moral ambiguity is nuanced and unpredictable. People high in imaginative resistance, those who do not want to explore the boundaries of their moral values, reported more curiosity for the clearer moral agents—the morally good and bad agents.

Taken together, our findings suggest that there is something to be learned from each moral agent. People do not seek out moral goodness alone (as we might expect from a desire to maximize good and minimize bad) or badness alone (as we might expect from their Netflix watching). Instead, explanation-seeking for morally good agents was driven by the perceptions of possible learning. Gaining additional information about how to emulate morally good people has value for day-to-day life, and it may also serve to reinforce the belief that people are good at heart^75–78^. We also found that perceptions of learning were high, and confidence was low for morally ambiguous agents. This may reflect the unpredictable nature of morally ambiguous agents. By selecting morally ambiguous people, we can satisfy the urge to know what it is that makes them so mysterious, and close an information-gap^71^.

While we found that explanation preferences for moral agents are related to their atypicality, people may nonetheless seek out different kinds of information depending on the kind of moral agent. People may want to know, for example, *why* people do bad things, but have different informational preferences for doing good things. To better understand what motivates the drive to seek out moral information, the next set of experiments pit information types—descriptive and explanation information—against one another for the different moral agents.

### Experiment 3

Experiment 3 tested whether people seek distinct information from morally ambiguous and bad people compared to morally good people. We suspected that people would seek different kinds of information about different moral agents. Specifically, people may want insight into the minds of morally ambiguous and bad people because it reveals *why* someone did what they did. For morally good agents, people may be more interested in who or how it looks to be morally lauded. As such, we expected that people would be more drawn to information specifically about the minds and motives of people are who are immoral compared to more descriptive information about those who are morally good. Here again, we tested whether, contrary to many theories of morality, moral badness does not unilaterally spark disengagement, avoidance, or withdrawal, but prompts curiosity for explanations. Using the same manipulation of moral status from Experiments 2a and 2b, we predicted that morally ambiguous and bad agents would pique curiosity about their minds and motives more than morally good agents. We also conducted a pilot experiment using fictional characters from television and movies and report those results in the Supplemental Materials. In general, the decision-making patterns from the pilot and Experiment 3 are consistent: People prefer to learn moral motive information about immoral agents.

## Method

### Design

We used a fully within-subjects design with 3 levels for Moral Agent Type (morally good, bad, and ambiguous). Participants made a single decision about which type of information (appearance or mental state) they would like for each of the three types of moral agents followed by judgments about that target.

### Participants

Data collection began online using the student subject pool at Brooklyn College and Queens College during the Spring 2019 semester in exchange for course credit. Due to COVID-19, the student subject pool was shut down. An a priori power analysis based on the pilot was conducted using PANGEA^79^ to accommodate the multi-level nature of the model using a small to medium effect size of *d* = 0.28. The power analysis suggested 270 participants was required that to achieve power of about 0.80, with alpha set to 0.05. We collected additional participants online using Prolific (accounting for about a 15% attention check failure rate). All participants, including those online and using the subject pool, provided informed consent. The only inclusion criterion for Prolific data collection was current United States residence and a 95% or higher rating on Prolific. Prolific participants were paid $3.40 on average for their participation and students participated in exchange for course credit. We recruited a total of adult (18 and older) 319 participants (74 from Queens College, 64 from Brooklyn College, and 181 from Prolific). We conducted our analyses on a final sample of 239 participants (*M*_age_ = 23.4, *SD*_age_ = 6.8, Male = 67, Female = 166, Other = 6) after removing participants who failed preregistered attention and comprehension checks (*N* = 80). Additionally, we tested for, but did not find, any statistical differences between the different samples and do not discuss them further.

## Materials

### Information seeking task

**Figure 7:**
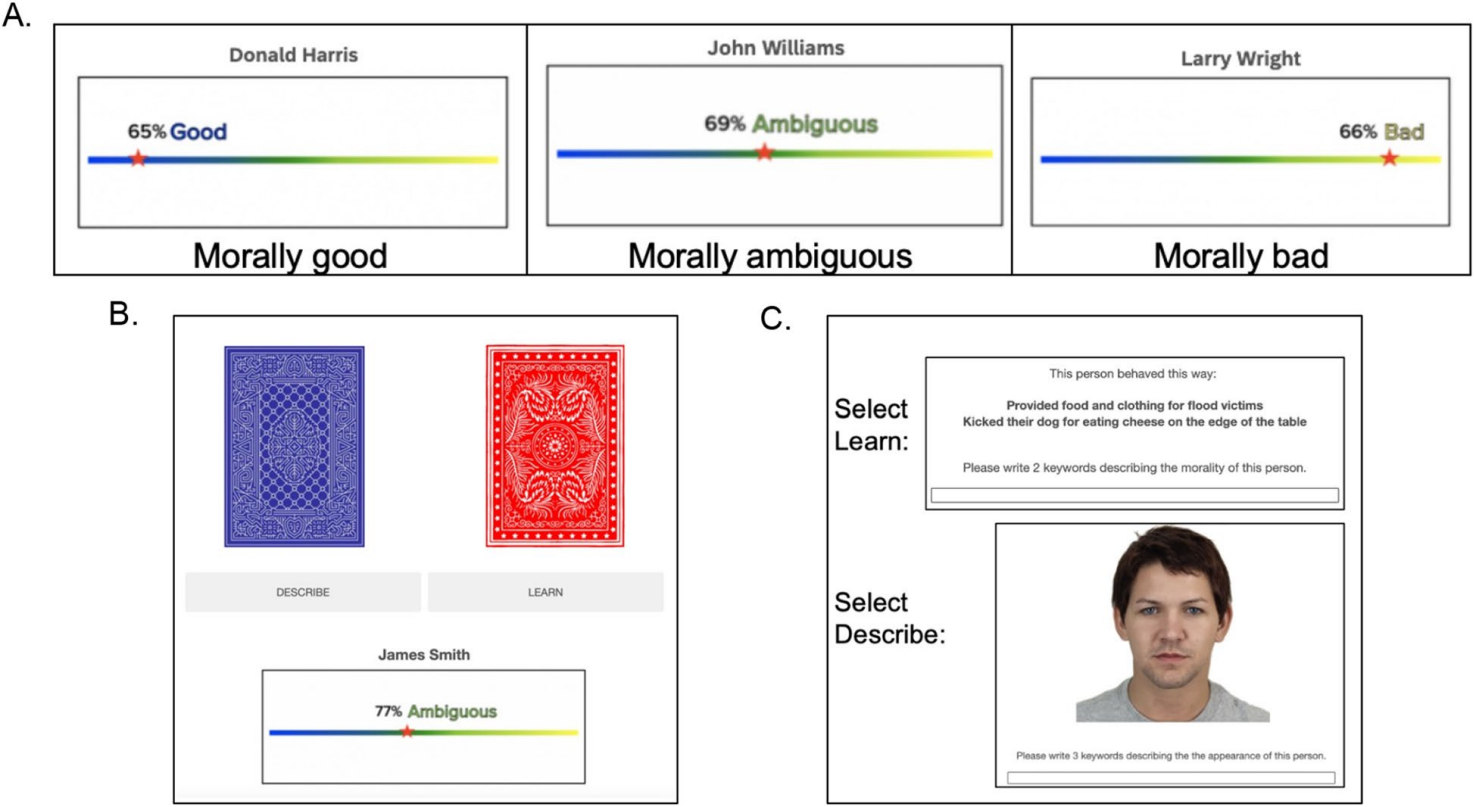
Panel A includes example images of the moral status manipulation (morally good, ambiguous, and bad, respectively). Panel B is an example of a single trial of the information seeking task. Panel C includes an example of the information that would be revealed if participants selected the Learn Deck or the Describe Deck on a given trial. The face pictured is reproduced with permission from the Chicago Face Database.

We adapted the information seeking task from Cameron et al.^42^. Participants saw two decks of cards. In the instructions portion of the experiment, participants were told that one deck was associated with “Learn” information, which provided explanation information about the moral agent (e.g., “you will be told about the actions and experiences of the named person”) and prompted participants to imagine the feelings of the agent, and the other deck was associated with “Describe” information, which provided descriptive information about the moral agent (e.g., “you will see an image of their face”) and prompted participants to resist imagining the feelings of the agent. The full description of the instructions is available in Supplemental Material. Participants were asked to select between the two decks to reveal the selected type of information for the moral agent presented (see Fig. 7A). The manipulation of moral status for the agents matched that of Experiments 2a and 2b. On each trial, if a participant selected “Describe”, they would see a neutral expression from a White male face selected from the Chicago Face Database (CFD;^61^) and matched on trustworthiness and attractiveness (see^80^). If a participant selected the “Learn” deck, they would see the moral actions of the person that led to their moral categorization (e.g., This person provided food for flood victims and kicked their dog) see Fig. 7B). For both decks, participants were also asked to write two keywords that describe the information they were shown and then answer questions about their similarity to the character, and how good and bad the agent was as a manipulation check. This task was used to see whether individuals preferred to learn more about *why* someone is an immoral agent (vs. access appearance information).

### Moral agent Stimuli

As in Experiment 2a and 2b, thirty first and last names were selected from a list of common United States names. Full list is available on the project OSF page. These names were combined using a random list and adjusted if the random pairing created a familiar name (e.g., Peter Jackson changed to Peter Johnson) and yoked to labels identical to Experiment 2a and 2b.

### Manipulation checks

Participants were asked to indicate how morally bad and how morally good the character they learned about (or saw) was following each decision in the main experimental task.

### Similarity

We also measured similarity at the trial level. We asked participants “Do you feel as though this person is like you?” rated on a scale from 1 = *Not at all like me* to 9 = *Very much like me*.

### Individual difference measures

#### Morbid curiosity

We again used the same six items from the Morbid Curiosity Scale (MSC;^81^) as used in Experiments 2a and 2b. These items were collapsed into a single MSC variable (α = 0.88).

### Other individual differences

We also measured Need for Cognition (NFC;^82–84^), Need for Consistency^85^ and Evil Essentialism (modified from^86^). These are reported in full in the Supplemental Material.

### Procedure

On each trial, participants saw the name of a person, a slider scale indicating their moral status, and the “Describe” deck and the “Learn” deck to select from. Moral status types were shown in completely random order. After making a selection for the moral agent presented, participants wrote two keywords that describe the information they were shown, and then answered manipulation checks and questions about similarity to each of the selected agents. [We also measured “interest” in the moral agents and report those results in the supplemental materials.] Participants completed 30 trials of the deck task where ten moral agents were rated good, ten rated ambiguous and ten rated bad. After completion of the main experimental procedure, participants completed individual difference measures and demographics questions. Finally, participants were debriefed and provided credit or paid for their participation. We also included deck color as a single between-subjects variable. For half of the participants, the “Describe” information deck was blue and “Learn” information deck was red, and for the other half of participants this was flipped. Deck color had no effect on results and will not be discussed further.

## Results

We preregistered running two mixed-effects models: One generalized mixed-effects model with participants and stimuli entered as random effects and Moral Agent Type as the predictor, and another with the same specification but including similarity as a covariate. We also used a linear mixed-effects model to test whether Moral Agent Type predicts self-reported interest and report those findings in the Supplemental Materials. For both models, conclusions remain similar with the addition of the covariate except for where noted. Each model includes by-participant random slopes and intercepts.

### Manipulation check

Results suggested that the manipulation was successful. Participants rated the good moral agents as more morally good than both the ambiguous, *b* = 1.53, *SE* = 0.07, *t*(238) = 22.36, *p* < 0.001, *r* = 0.82, *95% CI* [1.39, 1.66], and bad, *b* = 2.95, *SE* = 0.10, *t*(238) = 28.76, *p* < 0.001, *r* = 0.88, *95% CI* [2.75, 3.15] agents. Additionally, the bad moral agents were rated as more morally bad than both the morally good, *b* = 2.82, *SE* = 0.10, *t*(238) = 27.27, *p* < 0.001, *r* = 0.87, *95% CI* [2.62, 3.02] and morally ambiguous, *b* = 1.22, *SE* = 0.07, *t*(238) = 18.41, *p* < 0.001, *r* = 0.77, *95% CI* [1.09, 1.35] people.

### Moral curiosity for different kinds of information

**Figure 8:**
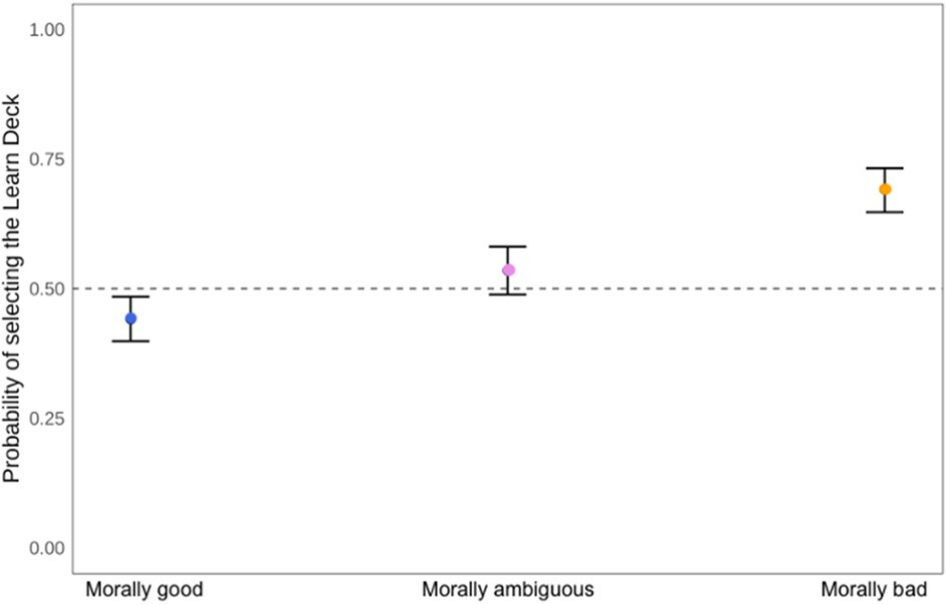
Probability of choosing the “Learn” deck for each of the Moral Agent Types. Error bars represent 95% Confidence intervals. This model includes similarity as a covariate, by-participant random slopes and random intercepts.

We used ‘glmer’ to accommodate the binary nature of the variable and along with the random intercepts and slopes for participants, we included by-participant random slopes to test which Moral Agent Type sparked the most moral motive explanation-seeking. Moral Agent Type was entered as a fixed effect, and similarity as a covariate (pattern of results remains consistent when excluding random slopes). Overall, results partially supported predictions. As predicted, relative to morally good individuals, morally ambiguous individuals elicited significantly more moral motive explanation-seeking, *b* = 0.27, *SE* = 0.08, *z* = 3.38, *p* < 0.001, *r* = 0.07, *95% CI* [0.11, 0.42], *OR* = 1.30. However, this effect did not hold when we excluded the preregistered covariate similarity (*p* = 0.38). Further, in contrast to our predictions, morally bad agents elicited significantly more explanation-seeking behavior than both the ambiguous and good agents. Bad agents lead to more “Learn” (vs. “Describe”) choices compared to both ambiguous individuals, *b* = 0.66, *SE* = 0.09, *z* = 7.65, *p* < 0.001, *r* = 0.18, *95% CI* [0.50, 0.83], *OR* = 1.93, and good individuals, *b* = 0.93, *SE* = 0.09, *z* = 10.45, *p* < 0.001, *r* = 0.25, *95% CI* [0.75, 1.10], *OR* = 2.53 (see Fig. 8). Moral valence drove seeking out moral motive explanations—people selected to learn “why” for morally bad and ambiguous agents but selected to learn descriptive information about morally good agents.

**Figure 9:**
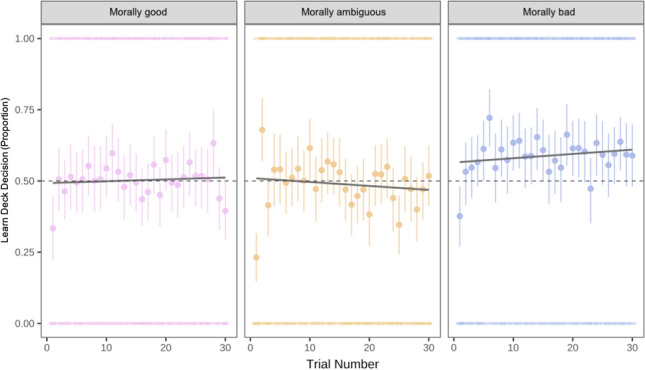
Moral agent selection over time for the three types: morally good, morally ambiguous, and morally bad. The dots indicate the average proportion of “Learn” deck across participants, and the vertical lines reflect 95% Confidence Intervals.

We also explored whether information preferences differed as the trials went on. We specified a generalized mixed effects model with by-participant random intercepts and with trial and moral agent type, and their interaction term entered as simultaneous predictors. Results suggested that there was no main effect of time in the experiment, *p* = 0.87; people selected more learn-information for bad agents relative to morally good agents throughout the task, *b* = 0.30, *SE* = 0.14, *z* = 2.21, *p* = 0.027, *r* = 0.08, *OR* = 1.01, *95% CI* [0.035, 0.58] (see Fig. 9). We also found that people spend more time on the Learn Deck than the Describe Deck (*p* = 0.008). Though we explicitly told participants that both decks will require the same amount of time in the instructions (see Supplemental Materials for full instructions), the Describe Deck was likely easier. We think this provides an even stronger test of our hypothesis; participants were opting to engage in the more effortful task specifically for the morally bad agents throughout the experiment.

### Individual differences

We also preregistered that we would explore the influence of individual differences on explanation-seeking curiosity. Overall, we found that morbid curiosity predicted more curiosity for explanations, *b* = 0.18, *SE* = 0.08, *z* = 2.42, *p* = 0.015, *r* = 0.05, *95% CI* [0.03, 0.33]. We report additional preregistered individual difference analyses in the Supplemental Material.

## Discussion

People were more likely to choose to learn *why* the morally bad and morally ambiguous agents were categorized the way that they were compared to good agents. Central to the present argument is that curiosity in the moral domain drives epistemic behaviors in unique ways: Moral badness and moral ambiguity are uniquely engaging because they pique curiosity for explanations (see also^87^). We also found that people are drawn to morally good agents, but for more descriptive information. Additionally, in a pilot experiment reported in the supplemental materials, we found this same general pattern for fictional heroes, antiheroes, and villains (e.g., Superman, Batman, and Lex Luthor, respectively)—people prefer to learn explanations about immoral (and ambiguous) characters. However, in the pilot experiment, we did not find significant differences between antiheroes and villains. We suspect that this is because our ambiguous novel agents are seen as more “average” while the ambiguous characters created to star in TV and movies are likely less typical than an everyday morally ambiguous person (e.g., Deadpool).

We found that over the course of the task, people more frequently selected learn information for bad agents, but no such patterns emerged for the other two types of moral agents. This provides further evidence that explanations are particularly satisfying for immoral minds. Finally, we expected that people high in morbid curiosity would select to learn about immoral minds more than those low in morbid curiosity, but that is not what we found. Instead, we found a small but significant positive correlation between morbid curiosity and Learn Deck selection. We think this is suggestive of a general interest in others’ mind for people high in morbid curiosity, but future work is needed to better understand how morbid curiosity affects information preferences. While we have found support for the claim that people are curious for explanations about immoral agents, it is not yet clear whether this drive to understand and engage—and specifically the enjoyment of ambiguity—is unique to the moral domain.

## Experiment 4

A great deal of research suggests that humans are cognitive misers^42,88–94^. We avoid cognitive effort when possible, and we are careful about the kinds of things we choose to expend cognitive effort to engage with—factoring in rewards^95^ and likelihood of errors^96^. The amount of cognitive effort expended to process a stimulus also affects our evaluative judgments of that stimulus. For example, when works of art are easy to (visually) process, people tend to rate them more positively^97^. In the context of risk evaluations, when words are difficult to pronounce, people tend to rate them as more risky and more harmful^98^. That it, there is a direct link between cognitive ease and affective evaluation. People largely prefer to engage with familiar, predictable, and easy to understand content.

Engaging with others’ minds is also a demanding task that often requires incentives to initiate^99^. The avoidance of effortful engagement in the social domain is especially consequential for empathy, which is critical to sharing and understanding the experiences of others^80^. When possible, people avoid feeling empathy for other humans, but do not do so for other living things like animals^100^. Altogether, research suggests that there is something uniquely taxing about engaging with the minds of other people. Yet, evidence from hours watched on Netflix (Study 1) and the desire to learn more about morally bad fictional characters (Supplemental Pilot Experiment) as well as novel people (Experiments 2a and 2b), suggests that deviance and ambiguity may be uniquely motivating to engage with in the moral domain. Further, in Experiments 2a and 2b, moral ambiguity uniquely piqued curiosity, despite people having relatively low levels of confidence about the kind of information that would be revealed (see Fig. 3). People seem to be curious about these otherwise disfluent, mysterious, contradictory people that require effortful engagement to understand. As such, we conducted Experiment 4 to test whether the attraction to ambiguity is specific to the moral domain.

Experiment 4 expands our understanding of moral curiosity by comparing moral to aesthetic ambiguity, testing the unique capacity of moral ambiguity to pique curiosity and promote explanation-seeking. Specifically, we tested whether curiosity and explanation-seeking was higher for ambiguity in someone’s moral character or in the aesthetic value of an artist’s work. Aesthetic judgments, like moral judgments, gain value from social consensus^101^ and are often directly linked (e.g., beauty and moral goodness;^102^). By comparing moral ambiguity to aesthetic ambiguity, we can better understand whether ambiguity in consensus-based judgments piques curiosity more generally or whether it is specific to the moral domain. If participants are uniquely interested in moral ambiguity, this would suggest that there is something unique about moral badness that spurs us to overcome the effort it takes to engage with ambiguity to approach and learn more.

## Method

### Design

The design mirrored Experiment 2a and 2b, but Phase 1 only included moral ambiguity and aesthetic ambiguity as decision options. We used a within-in subjects design to examine the effect of ambiguity status (morally ambiguous and aesthetically ambiguous) on moral curiosity. Phase 2 again asked participants about their judgments of their selection from Phase 1. Phase 3 revealed vignettes of motive information for the selected target. There was no memory task.

### Participants

We again preregistered our recruitment goal and exclusion criteria. We recruited 421 adult (18 and older) participants from Prolific with the goal of preserving a sample size of about 348. We conducted a power analysis using G*Power^98^ to detect an effect with an odds ratio of 1.4. After excluding people who failed attention (*N* = 29) and instructions comprehension checks (*N* = 2), we conducted analyses on a final sample of 391 participants (*M*_age_ = 39.84, *SD*_age_ = 14.36, Male = 190, Female = 194, Other = 6). The inclusion criterion for Prolific data collection was current United State residence, no participation in pilot studies related to this experiment, 99% approval rating on Prolific, use of a desktop or laptop computer for the experiment, and a minimum of 5 submissions of Prolific. Prolific participants provided informed consent and were paid $2.40 on average for their participation.

## Materials

### Phase 1: ambiguous agent selection

#### Ambiguity type stimuli

**Figure 10:**
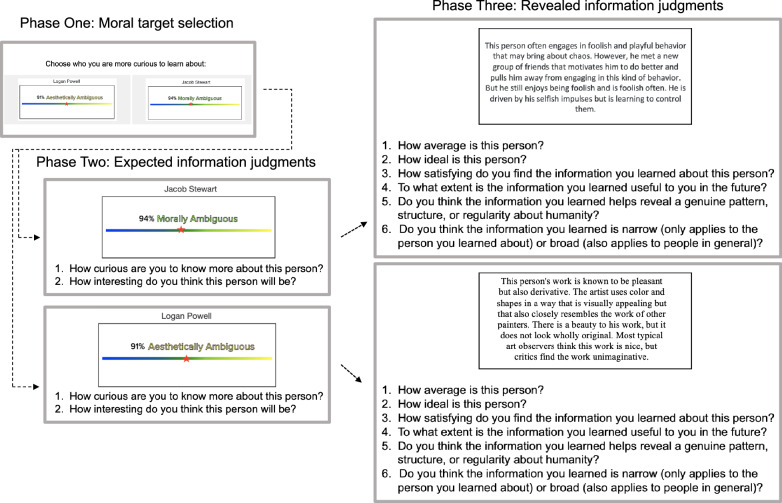
Three phases of Experiment 4 and items rated at each stage. Participants first select either a morally or aesthetically ambiguous agent, then in Phase 2, they make judgments about that selected agent, and then Phase 3 reveals additional information about the agent. The example stimuli are included in the Supplemental Material.

We used a subset of the same names from Experiment 2a and paired them with 20 total ambiguity sliders: 10 morally ambiguous and 10 aesthetically ambiguous. The ratings on the sliders (i.e., how ambiguous they were) for the two ambiguity types were matched across trials but jittered for realism on individual trials so they were not identical on any given trial. An example of the slider stimuli is presented in Fig. 10.

#### Ambiguity information seeking task

Here, we used the same decision task as in Experiment 2b but included only two possible options: moral ambiguity or aesthetic ambiguity. As in Experiment 2a and 2b, participants were instructed to select the agent that they wanted to learn more about. They were also told they could move freely from option to option and that the task would take the same amount of time regardless of the selection made. Specifically, participants were told:

To better understand what makes people curious, we will ask you to select between two different people in this task. To make your decision, we will show you the name of each of these people, and an accompanying “ambiguity score”. This score is calculated from other people's ratings about the mind/thoughts of a person or from ratings about the works of art created by a person (with their real names changed to pseudonyms). The ambiguity score you will see for each person is an average calculated from the responses of all the participants in that previous study. This means that on each trial of the task, you will see an option to select a person who was rated morally (which means concerned with right and wrong) ambiguous, and an option to select a person who created works of art that were rated as aesthetically (which means concerned with beauty or the appreciation of beauty) ambiguous. […] To make your decision, try to imagine the actions or the works of art that may have prompted others to rate the person as they did. Let yourself get caught up in imagining what the person feels, how they think, and what their motives or reasons for acting or for their art probably are. The goal of the task is to select the person who is most fascinating to you on each trial. […] Overall, this task will take the same amount of time regardless of which type of person you choose.”

### Phase 2: expected information judgments

#### Curiosity

During Phase 2, participants indicated their curiosity for more information about the selected target. We asked participants, “How curious are you to know more about this person?” rated on a scale from 1 = *Not at all curious* to 9 = *Extremely curious*.

#### Interest

Participants also indicated their confidence in their prediction about how the selected person will be. We asked participants “How interesting do you think this person will be?” rated on a scale from 1 = *Not at all* to 9 = *Extremely*.

### Phase 3: Revealed information judgments

#### Revealed moral information

In the final phase of the trial, selecting moral ambiguity revealed moral information about the target, and selecting aesthetic ambiguity revealed explanatory information about the body of work by the artist. No images were presented in this experiment. The information revealed was either morally ambiguous (describing someone who is both morally good and morally bad), or aesthetically ambiguous (describing someone whose works were rated as both good and bad; see Fig. 10 for task flow and examples).

#### Normality

The same measures of normality were used as in Experiment 2a-2b (7-point scales).

#### Satisfaction

We used the same measure of satisfaction as in Experiment 2a-2b.

#### Perceived learning

The same three items measured perceived learning. A utility of learning item, a pattern item, and a broadness of applicability item.

### Individual difference measures

#### Morbid curiosity

We included the same items to measure MSC. We collapsed these into a single variable (α = 0.94).

#### Imaginative resistance

We used the same three items to measure imaginative resistance items from Experiments 2a and 2b, and we collapsed them into a single index (α = 0.81).

#### Perspective taking

We also included the same three items to measure perspective taking as used in Experiments 2a and 2b. We again collapsed it into a single index (α = 0.87).

#### Procedure

Participants provided informed consent and then completed 10 trials each with three phases. The flow of the phases matched that of Experiment 2a-2b. After completing 10 trials, participants answered the individual difference measures, demographics questions, and were debriefed and compensated for their time.

## Results

The analysis strategy was similar to Experiments 2a and 2b. However, we preregistered conducting a mixed-effects logistic regression as the primary model in error. Given the nature of the task, like Experiments 2a and 2b, we conducted a Chi-square test to examine whether participants selected one option more frequently than the other. Mirroring Experiments 2a and 2b, we use mixed-effects models for all other analyses and specify random intercepts and slopes for participants. All models use aesthetic ambiguity as the reference group.

### Phase 1: target selection

#### Information seeking task

**Figure 11:**
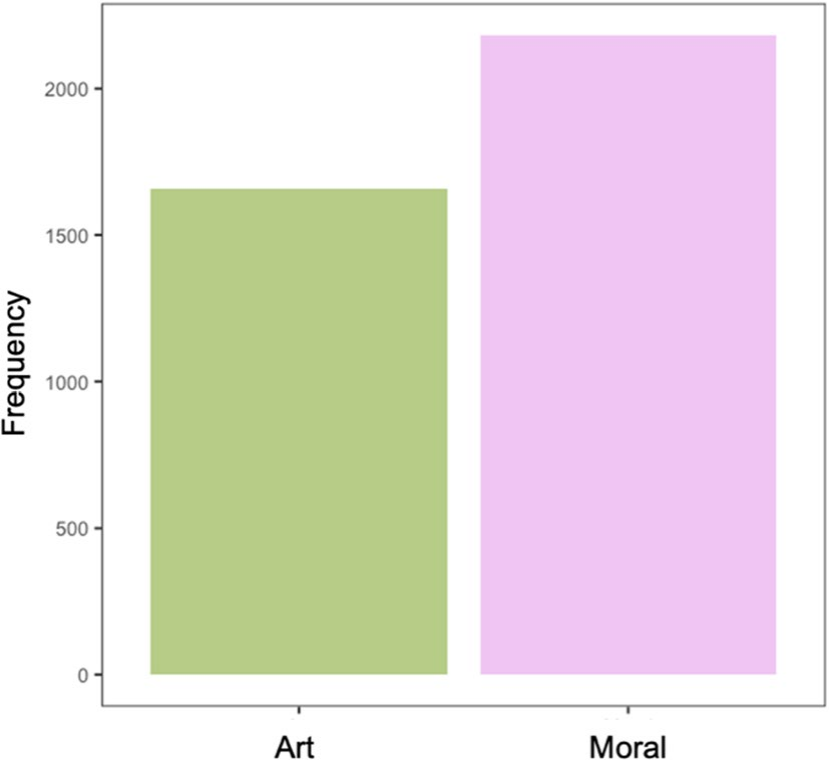
Total counts for the number of selected Ambiguous agents across participants. Agents were either morally ambiguous (labeled moral) or aesthetically ambiguous (labeled art).

To examine whether participants showed a preference to learn more about one type of ambiguity over the other, we conducted a Pearson Chi-square test. Results suggested that compared to what would be expected if participants were choosing at random between the two agents, participants more frequently chose morally ambiguous agents to learn more about *χ*^*2*^(1, *N* = 3910) = 72.39,* p* < 0.001 (see Fig. 11). People opted to learn more about moral ambiguity than aesthetic ambiguity.

**Figure 12:**
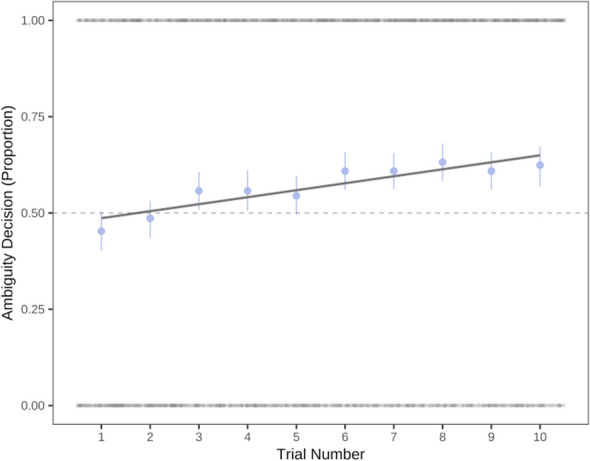
Proportion of moral choices over time on the ambiguous agent information-seeking task. Error bars reflect bootstrapped 95% confidence intervals.

We also explored whether choices differed across time. We conducted a generalized mixed effects model with trial entered as the predictor and by-participant random intercepts to examine how time affected preferences. Results suggested that time had a small, but statistically significant effect on preferences such that the more trials people saw, the more frequently they selected the moral agent, *b* = 0.08, *SE* = 0.01, *z* = 6.87, *p* < 0.001, *r* = 0.02, *OR* = 1.09, *95% CI* [0.06, 0.11] (see Fig. 12). One worry about the selection of moral targets over time is that it could reveal that the moral information provided was more satisfying than the aesthetic information for reasons besides a sustained interest in morally ambiguous targets (e.g., it was better-written). Critically, this preference for moral targets over time cannot be explained by satisfaction alone; satisfaction does not interaction with time to predict choice (*p* = 0.28), and when we include satisfaction as a covariate the effect of increased moral selections over time remains robust, *b* = 0.08, *SE* = 0.01, *p* < 0.001.

We also examined decisions on Trial 1 as it is possible that our effects are driven by the difference in social vs. non-social information. Moral information is inherently more social than information about artistic ability (though we did try to include information about the artist’s motives to some extent), and people tend to be interested in social information. We reasoned that if this were the case, participants would choose the moral trials more often on Trial 1, but we do not find a significant difference. Instead, we find a marginally significant effect in the opposite direction—people select the aesthetic target on Trial 1 marginally more often *b* = −0.21, *SE* = 0.11, *t* = −1.90, *p* = 0.06.

### Phase 2: expected information judgments & phase 3: revealed information judgments

**Figure 13:**
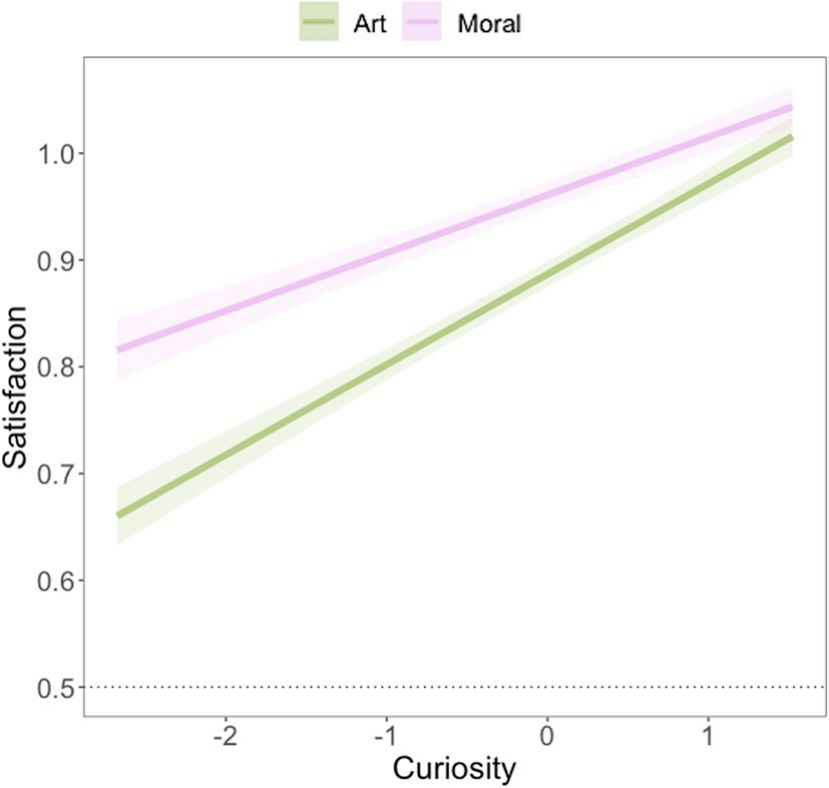
Interaction between curiosity (scaled) and Phase 1 ambiguity target selection. Confidence intervals represent ± 1 SE. Model is fit with random intercepts and slopes for participants.

For Phase 2 and 3, we report abbreviated results. Full statistical analyses are reported in the Supplemental Material. Contrary to our predictions, there were no significant differences between Phase 1 ambiguity decision on ratings of curiosity or interest, further supporting the idea that the moral trials were not necessarily more interesting by nature of being social than the aesthetic trials. For normality, aesthetically ambiguous information was rated as more normal than morally ambiguous information, [ideal: *b* = 0.45, *SE* = 0.05, *p* < 0.001; average: *b* = 0.56, *SE* = 0.06, *p* < 0.001]. Results also suggested that morally ambiguous agents were more satisfying to learn about than aesthetically ambiguous ones,* b* = 0.31, *SE* = 0.06, *t*(372) = 5.21, *p* < 0.001, *r* = 0.26, *95% CI* [0.19, 0.43]. When we entered curiosity, decision type, and their interaction term to predict satisfaction, results yielded a statistically significant interaction, *b* =  − 0.06, *SE* = 0.03, *t*(1289) =  − 2.37, *p* = 0.018, *r* = 0.07, *95% CI* [−0.12, −0.01], such that the more curiosity participants reported, the more satisfaction they felt, particularly for the aesthetic domain (see Fig. 13). For those people who are curious about art, the revealed information at Phase 3 was more satisfying than those who reported low curiosity for the aesthetic agents. Lastly, compared to aesthetic ambiguity, moral ambiguity was judged as having greater utility for learning (*b* = 0.64, *SE* = 0.05, *p* < 0.001), more reflective of genuine patterns (*b* = 0.70, *SE* = 0.06, *p* < 0.001), and applying more broadly (*b* = 0.29, *SE* = 0.07, *p* < 0.001). We report full results (controlling for other measured variables) for satisfaction and utility for learning in Table 4 (as in Experiments 2a/2b these were exploratory analyses and should be interpreted with caution). Overall, people report being more satisfied to learn about moral agents than aesthetic ones, and that they have learned more.

**Table 4:** Estimates for simultaneous predictors of utility of learning and satisfaction in Experiment 4.

	Perceived utility of learning	Satisfaction				
Variable	Beta	95% CI^1^	*p*-value	Beta	95% CI^1^	*p*-value
**Ambiguous Agent Selection**			**0.019**			** < 0.001**
*Art*	–	–		–	–	
*Moral*	0.31	0.05, 0.57		0.53	0.25, 0.81	
**Curiosity**	0.13	0.10, 0.16	** < 0.001**	0.10	0.07, 0.13	** < 0.001**
**Average**	− 0.05	− 0.07, − 0.02	**0.001**	− 0.14	− 0.17, − 0.11	** < 0.001**
**Ideal**	0.01	− 0.02, 0.04	0.36	0.41	0.38, 0.44	** < 0.001**
**Satisfaction**	0.24	0.21, 0.27	** < 0.001**			
**Reflects a pattern**	0.24	0.21, 0.27	** < 0.001**	0.11	0.08, 0.14	** < 0.001**
**Broadness of applicability**	0.02	0.00, 0.05	0.086	− 0.01	− 0.04, 0.02	0.43
Ambiguous selection * curiosity			0.63			**0.004**
Moral * curious	0.01	− 0.03, 0.05		− 0.06	− 0.11, − 0.02	
**Utility of learning**				0.28	0.26, 0.31	** < 0.001**

^1^CI = Confidence Interval.

Significant values are in bold.

### Individual difference measures

Lastly, we examined whether individual difference measures (see Table 5 for correlations among variables) moderate the relationship between Phase 1 decision selection and Phase 2 curiosity. We again specify both random intercepts and slopes and report imaginative resistance and perspective-taking in the main text. The patterns of results for morbid curiosity are reported in the Supplemental Materials.

**Table 5:** Correlations among individual difference traits for Experiment 3.

Measure	1	2	3
1. Morbid curiosity	–		
2. Imaginative resistance	− 0.40***	–	
3. Perspective-taking empathy	0.12*	− 0.02	–

*p < 0.05, **p < 0.01, ***p < 0.001. Holm method for *p*-value adjustment.

**Figure 14:**
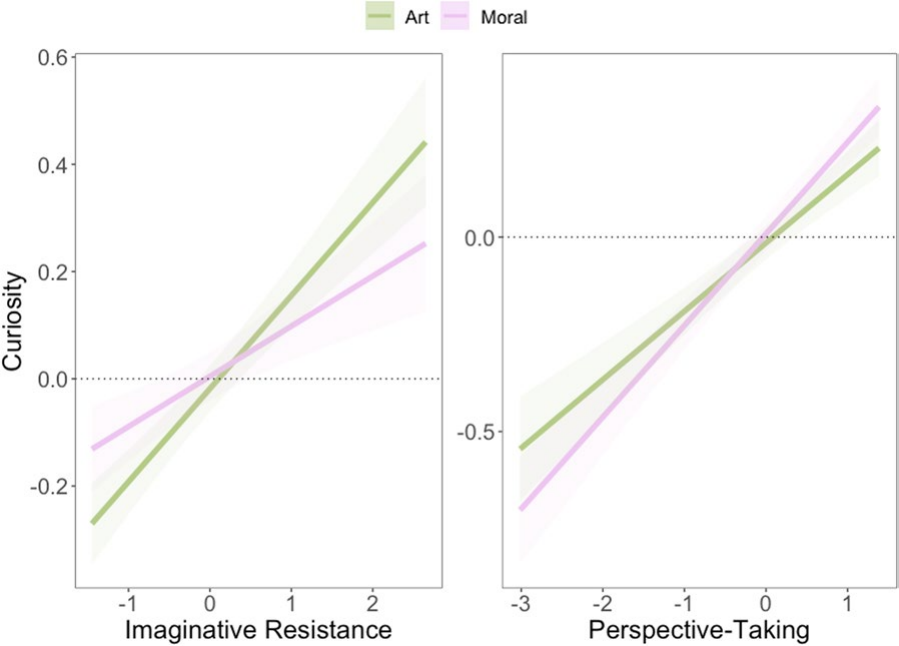
Interaction between individual difference measures and Phase 1 ambiguity selection. The patterns flip for imaginative resistance and perspective-taking. Confidence intervals represent ± 1 SE. Model is fit with random intercepts and slopes for participants.

When we entered imaginative resistance, Phase 1 decision, and their interaction term, imaginative resistance, but not Phase 1 decision (*b* = 0.03, *SE* = 0.05, *p* = 0.521), significantly predicted curiosity, *b* = 0.33, *SE* = 0.08, *t*(394) = 4.06, *p* < 0.001, *r* = 0.20, *95% CI* [0.17, 0.48]. There was also a significant interaction, *b* =  − 0.14, *SE* = 0.05, *t*(369) =  − 3.02, *p* = 0.003, *r* = 0.16, *95% CI* [−0.23, −0.05]. Participants high in imaginative resistance reported more curiosity for the aesthetic domain than they did for the moral domain (see Fig. 14). Those who do not wish to imagine alternate moral possibilities prefer to engage with aesthetic ambiguity over moral ambiguity; this further supports the interpretation that results are specific to the moral (rather than merely social) domain.

Perspective-taking empathy showed a similar pattern of results. Perspective-taking, but not Phase 1 decision (*b* = 0.03, *SE* = 0.05, *p* = 0.506), was a significant predictor of curiosity, *b* = 0.32, *SE* = 0.08, *t*(390) = 3.98,* p* < 0.001, *r* = 0.20, *95% CI* [0.16, 0.48]. Additionally, there was a significant interaction between the two, *b* = 0.10, *SE* = 0.05, *t*(347) = 2.18, *p* = 0.030, *r* = 0.12, *95% CI* [0.01, 0.19], such that the more perspective-taking participants reported, the higher their reported curiosity for the morally ambiguous agents (though the difference in slopes was not as large as for imaginative resistance; see Fig. 13).

## Discussion

Overall, we found that participants were more likely to select the morally ambiguous agents compared to the aesthetically ambiguous ones. They reported greater satisfaction and perceived learning for moral relative to aesthetic agents. However, we did not find the predicted difference in self-reported curiosity, which deviates from the pattern of results in Experiments 2a and 2b. Relative to another domain in which consensus, rather than objectivity, drive value judgments, participants view them as equally intriguing. We take this to be preliminary, suggestive evidence of the uniqueness of moral ambiguity, but more research needs to be done to investigate this question. While self-reported curiosity for these categories was similar, selection preferences and differences in satisfaction and learning suggest that moral ambiguity draws in curiosity and provides an avenue for learning that differs from other sources of ambiguity. This curiosity might lead to sustained engagement with morally ambiguous but not aesthetically ambiguous content.

Morally ambiguous agents were also rated as less ideal and less average than aesthetically ambiguous agents, even though both target categories had the same average amount of explicit ambiguity in our manipulation. For moral ambiguity, the ideal judgments were below the mid-point (though the average judgments were not). This suggests that participants see the morally ambiguous agents as being somewhat morally bad, but they nonetheless still pique interest, are preferred, and provide affective and learning benefits. Moreover, participants who were dispositionally inclined to perspective-take, and especially those low in imaginative resistance reported more curiosity for the morally ambiguous agents than the aesthetically ambiguous ones. People who do not want to engage with the minds of immoral agents also do not show approach tendencies toward that information. We think this pattern speaks to the specificity of *moral* curiosity for ambiguity.

An important limitation in this experiment is that we do not test a full array of moral and aesthetic agents (good, bad, ambiguous, average) nor do we pit decisions against each other (selecting morally good vs. morally ambiguous compared to aesthetically good vs. aesthetically ambiguous). We opted for a simpler design with ambiguity for a few reasons. First, we reasoned that ambiguity would be the strongest test of our predictions; both engaging with ambiguity and engaging in empathy are effortful and people dislike cognitive effort. In this way, the ambiguity trials provide a stringent test; the moral ambiguity trials may seem more effortful than the aesthetic ambiguity trials (and this may be reflected in a marginal effect for selecting the aesthetic trials on Trial 1). Second, it is difficult to disentangle moral ambiguity from other forms of social ambiguity like warmth as these dimensions may all collapse into one^81^. We also thought aesthetic ambiguity might be interesting given the popularity of shows about people attempting to make beautiful things like *Great British Baking Show* and *Project Runway*. That said, our results do not directly speak to whether people would select moral agents over aesthetic agents for good, bad, and average agents. It is possible that morality dominates in all of these decisions, in keeping with prior work on the dominance of morality, specifically over competence, in person perception^52,103,104^. If this is the case, our work adds nuance to this general phenomenon, suggesting people prefer negative and ambiguous moral information, even though we might expect information about bad and inconsistent people in the world to be more aversive than bad or inconsistent art (which can sometimes be played for laughs, as in programs like *Nailed It* in which people fail at elaborate baking projects).

### General discussion

Why do we spend hours watching a stalker, a scammer, a drug dealer on our screens? These characters commit acts of violence and theft, yet we spend hours engaging with them, often rooting for their success. This research started with an under-theorized aspect of moral life: moral ambiguity and moral badness are fascinating. Antiheroes––ambiguous moral figures in popular culture––and villains elicit our curiosity, and we devote countless hours to watching them. Here, we offered an initial investigation into the drivers of moral curiosity, and some of its functions. We found evidence that the majority of the most watched shows on Netflix feature immoral protagonists, and that immorality and hours watched were correlated—the more immoral the protagonist, the more hours people spent watching the program. We also found that, across all experiments, moral badness piqued curiosity, prompting explanation-seeking behaviors, though people opted to learn about both bad and good moral extremes when only moral motive information was available. When the kind of information available was manipulated, people more often chose to learn explanatory information about the morally ambiguous and bad people compared to the morally good ones. People also demonstrated a behavioral preference for learning about people who were immoral, and curiosity for explanations of ambiguous others was greater for the moral compared to the aesthetic domain. These results provide experimental evidence that we are drawn to moral badness, despite theorizing that we should avoid it (see also^43^). People are curious about morally and ambiguous people, especially *why* they do the things that they do.

Overall, our results build on and fit well within the rich body of literature that demonstrates the function of domain general curiosity. For example, some research suggests that curiosity often tracks functional value; curiosity for explanations can be driven by future-oriented expectations, like expected learning^30^. These findings extend this work into the moral domain and show that many of these same motives and functions drive curiosity toward moral agents, but that moral agents are unique in important ways. Namely, people seek explanations for negativity in the moral domain—ambiguity and badness are more closely linked and elicit more curiosity to learn explanations about the minds of others than goodness. Further, while people recognize that morally average agents have the most potential to teach them about day-to-day morality and the realities of our moral worlds (i.e., are statistically common), people are not drawn to them. We suggest that people whose morality differs from our own provides a learning opportunity—to learn about the inner workings of a mind different from our own or the ones we are familiar with—they tell us about the boundaries of our moral worlds.

This inquiry provides new insight into the seemingly paradoxical delight of morally bad things, which we suggest motivate exploration, and elicit specific explanation-seeking behaviors that are critical to learning^6–8^. This is especially interesting given that evidence presented across the experiments shows that it is not always obvious to people what draws them toward specific moral agents. For example, in Experiment 2b, we found that people learn the most (in an incidental memory task) about immoral agents, even though they reported learning the most from morally good ones. This evidence fits with previous research (e.g.,^30^) suggesting that perceptions of learning and actual learning can come apart. It is possible that judgments of what piques curiosity and what actually motivates information-seeking behavior come apart as well. Taken together, these results provide a path for understanding what moral curiosity is, and why people are drawn to learn about moral ambiguity and immorality in real life.

We also set out to examine the individual differences that best explained moral curiosity and the variance in preferences seen in real life and in our data. Across all studies, Morbid Curiosity consistently predicted more curiosity for immoral agents. We also found preliminary evidence that imaginative resistance, an unwillingness to explore alternate moral possibilities, and perspective-taking traits play a role in determining what kinds of moral content people choose to learn more about. People who were high in imaginative resistance were consistently more curious about morally good agents than those low in imaginative resistance. Looking to trait perspective-taking, there also seems to be a unique cost to exploring moral badness. Greater trait perspective-taking predicted more curiosity for morally good agents, and it predicted greater curiosity for moral compared to the aesthetic ambiguity. These findings suggest that a major contributor to the kind of moral content that people are curious about is a combination of morbid curiosity on the one hand, and perspective-taking ability and imaginative resistance on the other. Further, the link between perspective-taking and curiosity for moral goodness also fits well within the larger body of literature on goodness and morality. People believe that the true self is both good and moral^105,106^, and this motivation to construe the moral self as good shapes how people remember their own past transgressions^107^. Morality dominates person perception and formation of self-concept^3,103,108,109^, even in children^110^. These individual difference findings suggest that some people may perceive a cost to engaging with the minds of those who are morally bad because being morally good is important to adaptive social behavior. They also provide some evidence that the curiosity and learning we investigate here is specific to the moral domain.

The centrality of moral goodness is echoed in the attention that psychologists have given the topic. Moral psychological theory has focused extensively on identifying what people and cultures deem to be morally valuable, and how we deride and want to punish those who commit immoral actions. Theorists have pointed to harm (e.g.,^111–113^; but see^114^), to many intuitions (e.g.,^115–117^ but see^118^), and to communication (e.g.,^119^ but see^120^, alongside many other concepts, as unifying lenses from which to understand morality. Each of these theoretical perspectives, regardless of their approach, share the fundamental assumption that people should, and often do, seek to maximize moral good. However, this assumption does not always seem to be true (see^121^). When looking instead to everyday human behaviors and preferences, people are not always drawn to maximizing moral good (see also^122,122^; see^124^ for review). Here, we demonstrated that moral ambiguity and moral badness can be interesting and engaging, which conflicts with models, both moral and not, that suggest we are driven to seek out goodness and pleasure, and to avoid badness and pain (see also^125^). There currently exists no parsimonious way to integrate many of these surprising findings in morality—findings that suggest that people are often drawn to learn about and engage with immorality (not to mention commit actual acts of violence and aggression). We posit that people seek out explanations about the minds of morally bad and ambiguous others as a way to hone and construct an accurate view of the moral world, even if it means engaging with morally bad ideas. This framework aligns with previous research that suggests humans excel at learning about bad others—even when the information is inconsistent^23,126^.

Indeed, real-world, revealed preferences^127^ suggest moral ambiguity and moral badness are interesting and likely activate approach motivation. We do not avoid stories about morally bad people, but instead flock to them. By using an epistemic lens to peer through and comprehend moral cognition, we can understand this behavior and what motives it. Moral cognition is likely not only for maximizing moral goodness, but in the service of helping us make sense of our moral worlds, the good and the bad. Taken together, this research provides the first evidence for the functions and epistemic consequences of moral curiosity. While there are many epistemic emotions for which morality may be tuned, curiosity is a powerful drive that contributes to several important information-seeking and explanation-seeking behaviors. For instance, while surprise is typically regarded as the first reaction to schema-inconsistent information^128^, curiosity often follows and promotes exploratory behaviors and personal growth^129^. Curiosity is critical to knowledge acquisition, rationality, and other largely epistemic functions^13,14,130^. These findings suggest that moral curiosity guides knowledge seeking in ways that mirror domain general curiosity, but the antecedents and functions differ in important ways. Moral curiosity prompts us to explore and understand the morality of others, of ourselves (i.e., self-knowledge), and our society, our moral words—and partly explains why we spend so many hours watching people behave badly on our TV screens.

## Supplementary Information

Supplementary Information.

## Data Availability

The datasets generated during the current studies are available in the project’s page on the Open Science Foundation repository, [https://osf.io/2ucxt/].
