# Supplementary material for: People are curious about immoral and morally ambiguous others
Source: Sci Rep. 2023 May 5;13:7355. doi: 10.1038/s41598-023-30312-9 (PMC10162000; doi:10.1038/s41598-023-30312-9)
Supplement: Supplementary file 1 — Supplementary Information. [file 41598_2023_30312_MOESM1_ESM.docx]

**Supplemental Materials**

Table of Contents

[Materials and Stimuli 2](#_Toc117177586)

[**Experiment 2a-2b** 2](#_Toc117177587)

[**Instructions** 2](#_Toc117177588)

[**Full List of Vignette Stimuli** 3](#_Toc117177589)

[**Experiment 2a Additional Measures** 4](#_Toc117177590)

[**Experiment 3** 5](#_Toc117177591)

[**Instructions** 5](#_Toc117177592)

[**Full List of Learn Deck Stimuli** 6](#_Toc117177593)

[**Experiment 4** 7](#_Toc117177594)

[**Instructions** 7](#_Toc117177595)

[**Full List of Vignette Stimuli** 7](#_Toc117177596)

[Pilot Experiment: Exploring curiosity for fictional characters 11](#_Toc117177597)

[**Method** 11](#_Toc117177598)

[**Results** 13](#_Toc117177599)

[Additional Analyses 15](#_Toc117177600)

[**Study 1** 15](#_Toc117177601)

[**Experiment 2a** 17](#_Toc117177602)

[**Experiment 2b** 23](#_Toc117177603)

[**Experiment 3** 31](#_Toc117177604)

[**Experiment 4** 33](#_Toc117177605)

[Model Comparisons 35](#_Toc117177606)

[**Experiment 2a** 35](#_Toc117177607)

[**Experiment 2b** 41](#_Toc117177608)

[**Experiment 3** 48](#_Toc117177609)

[**Experiment 4** 49](#_Toc117177610)

[Supplementary References 53](#_Toc117177611)

# **Materials and Stimuli**

**OSF: (https://osf.io/2ucxt/)**

##

## **Experiment 2a-2b**

### ***Instructions***

In this task, we will ask you to select among four "types" of people.

To make your decision, we will show you their name, and a “**morality score**". The score is from a previous study that we did. In that study, we had about 70 participants read about some people (with their real names changed to pseudonyms) and rate them in terms of how morally good, bad, ambiguous, and average they are.

The morality score you will see for each person is an average calculated from the responses of all the participants in that previous study.

Your task is to choose who you would like to learn more about based on their morality score.

To make your decision, try to imagine the morality and motives of the people, letting yourself get caught up in imagining what this person feels, how they think, and what their motives or reasons for acting probably are.

When you make a selection, you will answer some questions and then get to see a description of that person.

**This means that you will be able to learn about the actions, moral motives and mind of the person you select.**

After you make a selection, you will be asked to make some judgments about them. Then, the motives and morality of the individual will be revealed in short description, and you will be asked to make some additional judgments.

The goal of the task is to select people who seem **most fascinating to you**. When people follow what they are naturally curious about, learning tends to be better. After you make decisions about several people, we will test your knowledge of them.

For example, you will see a series of trials that looks like this:

Choose who you want to learn more about:


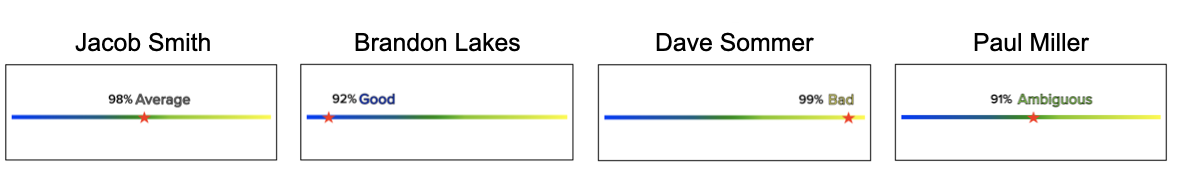


Your task is to select the person you would like to have the motive and moral information revealed for.

One person will always be from the group of people rated morally "average", one from the group of people rated morally "good", one from the group of people rated morally "bad", and one from the group rated morally "ambiguous".

You are free to choose whatever kind of person on any trial, and are free to move from person to person. If one kind of person begins to seem preferable, feel free to choose that category more often.

Overall, this task will take the same amount of time regardless of which type of person you choose.

***LIWC analyses on Phase 3 information***

Table S1 includes summary LIWC data for the stimuli used in both Experiments 2a and 2b.

**Table S1.** Example of stimuli revealed for each Moral Agent Type in Experiment 2a-2b.

| **Moral Agent** | **Word Count** | **Care Virtue** | **Care Vice** |
| --- | --- | --- | --- |
| Morally Bad | 61.7 | 0.5 | 1.5 |
| Morally Ambiguous | 65.1 | 1.2 | 0.3 |
| Morally Average | 62.6 | 1.3 | 1.4 |
| Morally Good | 63 | 3.0 | 0.3 |

### ***Full List of Vignette Stimuli***

**Table S2.** Table of all revealed stimuli for Experiment 2a-2b. All stimuli had the same set up as the first row in the following table, However, faces have been removed to comply with copyright restrictions.

| Morally Ambiguous | Morally Bad | Morally Good | Morally Average |
| --- | --- | --- | --- |
| 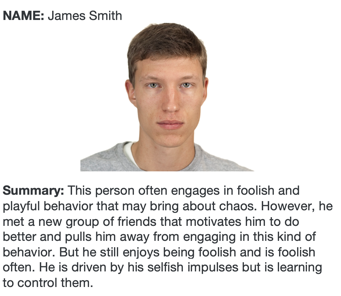 | 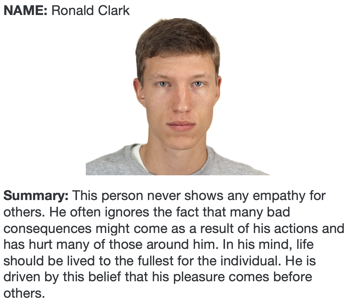 | 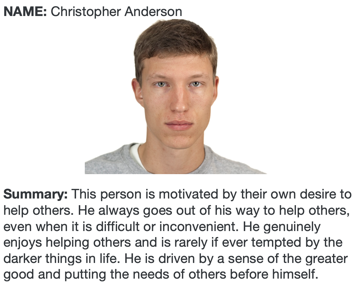 | 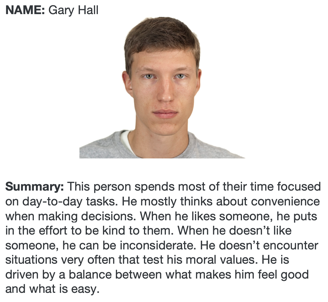 |
| 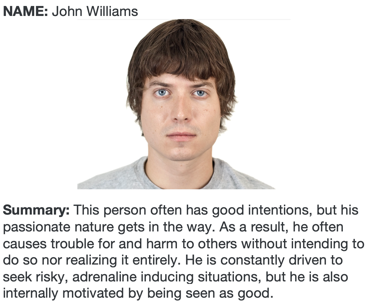 | 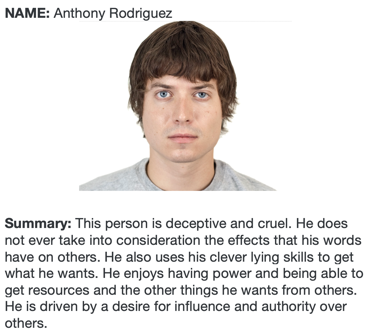 | 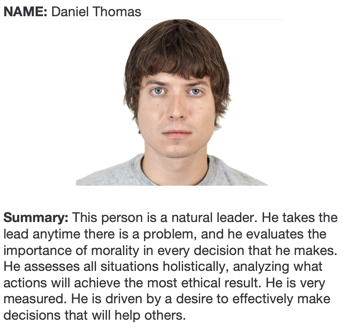 | 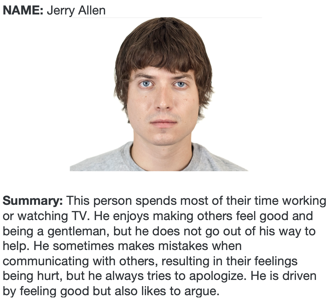 |
| 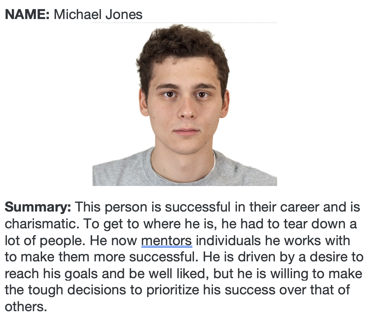 | 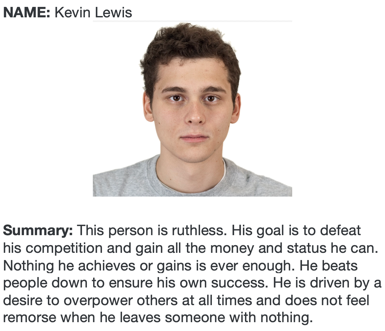 | 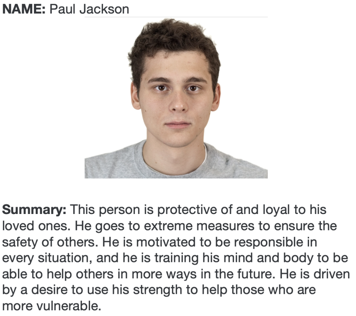 | 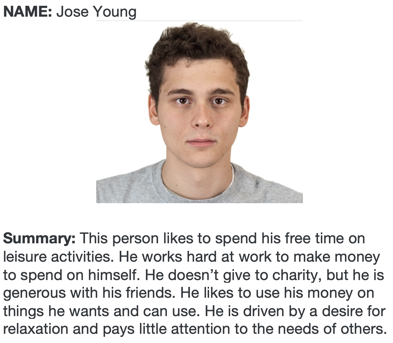 |
| 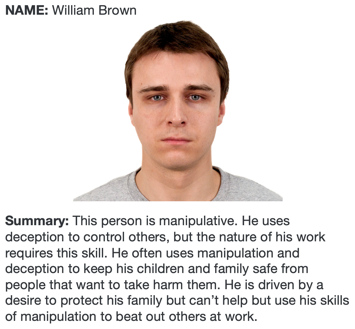 | 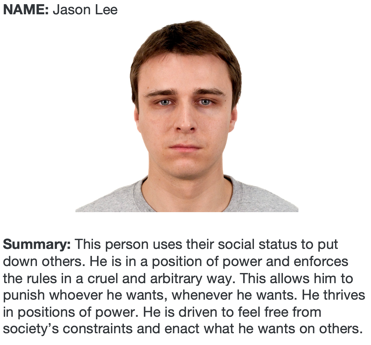 | 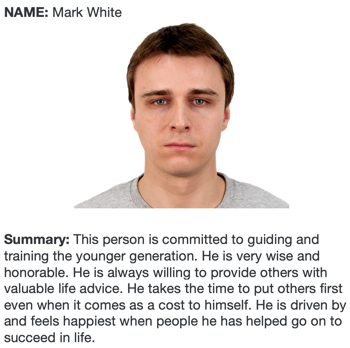 | 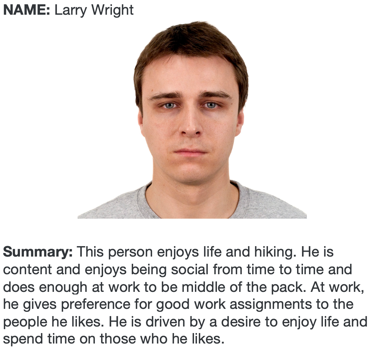 |
| 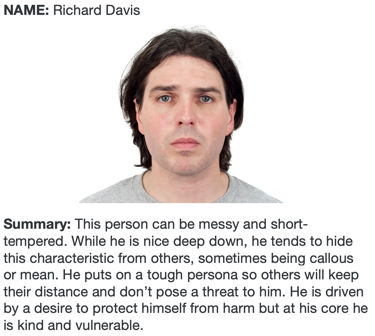 | 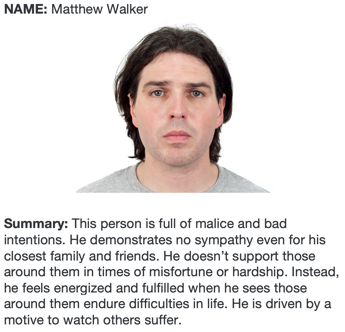 | 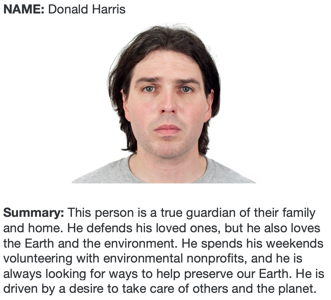 | 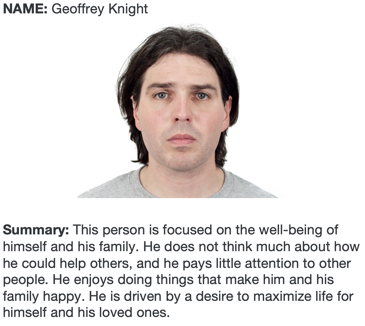 |
| 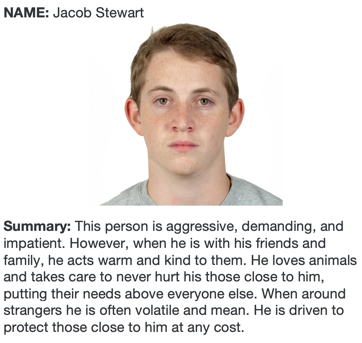 | 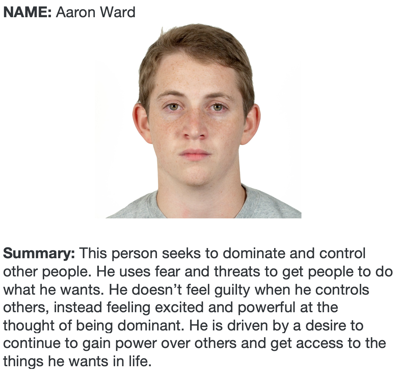 | 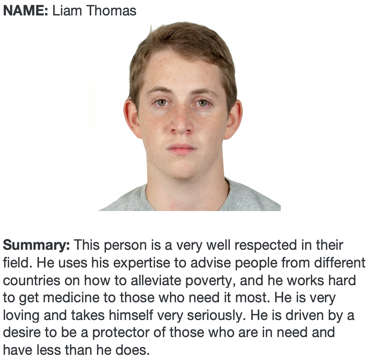 | 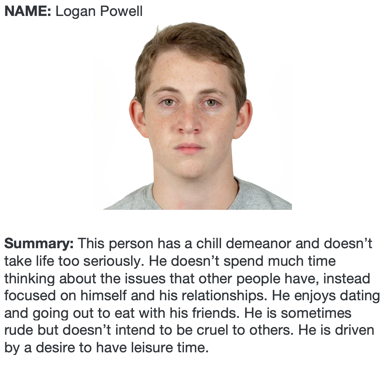 |
| 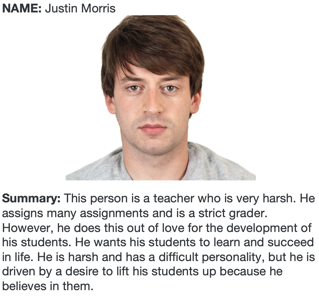 | 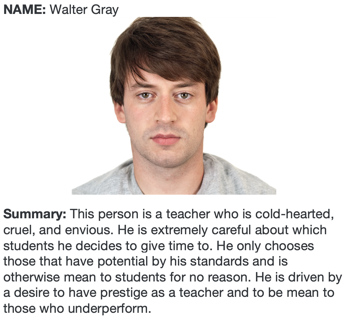 | 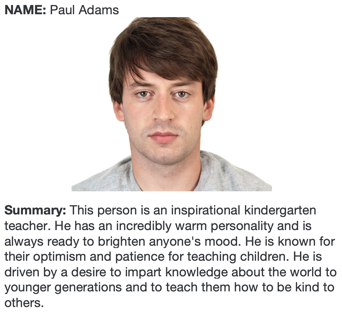 | 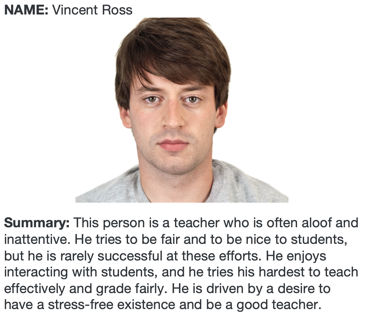 |
| 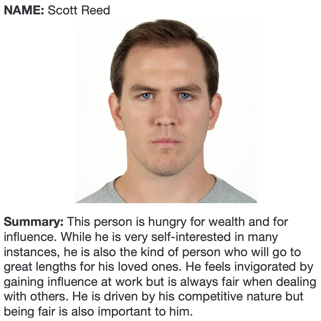 | 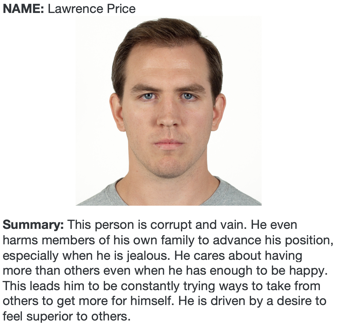 | 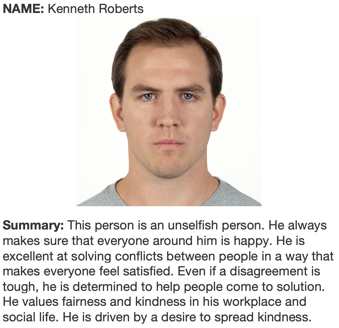 | 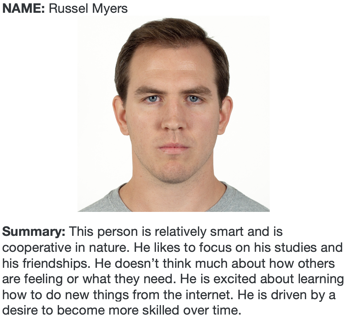 |
| 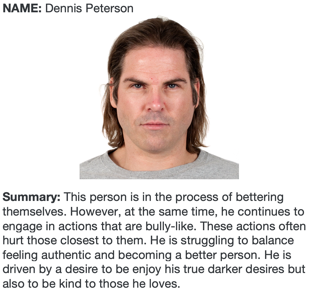 | 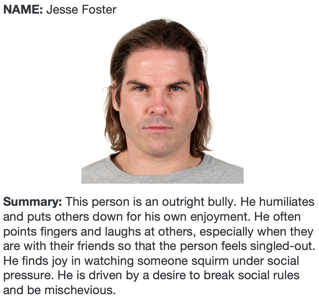 | 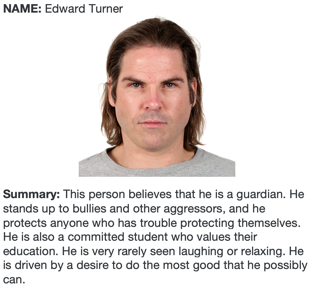 | 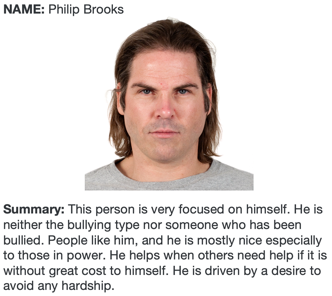 |
| 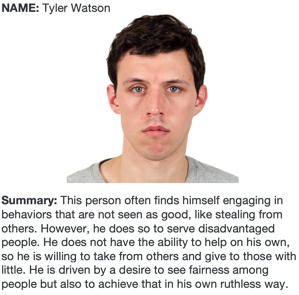 | 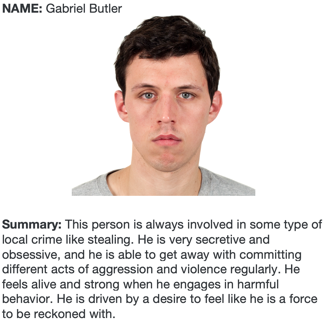 | 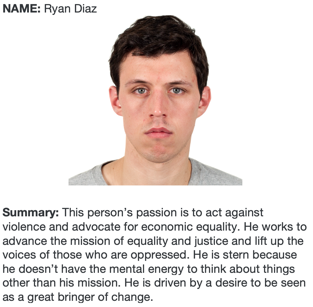 | 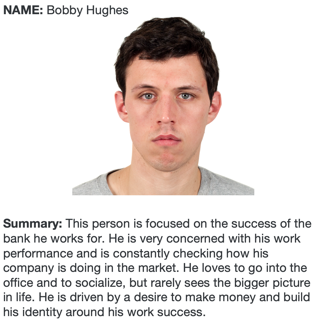 |

### **Experiment 2a Additional Measures**

**General and Personal Belief in a Just World Scale.** We also included Belief in a Just World [106] as a measure of moral worldview. A representative sample item is “I think basically the world is a just place”, rated from 1 = *strongly disagree* to 6 = *strongly agree*. This measure also had high internal reliability and was collapsed into a single variable (α = .93).

##

## **Experiment 3**

### ***Instructions***

In this task, you will complete a series of trials. On each trial, you will see two decks of cards, the name of a person, and a morality score. In a previous study, we had about 70 participants learn about these people and rate them on how morally good or bad they were. The morality score you see, is an averaged score calculated from all of their responses. 

Your task is to choose between these two decks based on the morality score you see. One of the decks is called the **Describe** deck and one is called the **Learn** deck. You will learn more about the decks on the next pace. Once you choose a deck, you will then be asked to answer some questions about the person.

On the **DESCRIBE** trials, you will see an image of their face. When completing this kind of trial, you will be asked questions after seeing the person, and you should try to be as objective as possible. To be objective, do not let yourself get caught up in imagining what this person feels, and what their motives or reasons for acting are.

On these trials, after reading the additional information, please provide two keywords to describe the physical appearance of the person, as if you were describing them to a sketch artist. (Example: “White, long hair” or “woman, blue eyes”). It is ok to use the same keywords multiple times, just make sure that you are accurately describing the physical appearance of the person in the image (e.g., age, gender, race, etc.).

On the **LEARN** trials, you will be told about the actions and experiences of the named person. When completing this kind of trial, try to understand their morality and motives as much as possible. To be understanding, let yourself get caught up in imagining what this person feels, how they think, and what their motives or reasons for acting are.

On these trials, after reading the additional information, please provide two keywords to describe the motives of the person. (Example: “survival, family” or “revenge, justice”). It is ok to use the same keywords multiple times, just make sure that you are a describing the motives of the person in the image.

You are free to choose from either deck on any trial and should feel free to move from one deck to the other whenever you choose. If one deck begins to seem preferable, feel free to choose that deck more often.

Overall, this task will take the same amount of time regardless of which deck you choose.

**Table S3.** Example of stimuli revealed for Describe Deck and Learn Deck in Experiment 3.

| Describe Deck | Learn Deck |
| --- | --- |
| 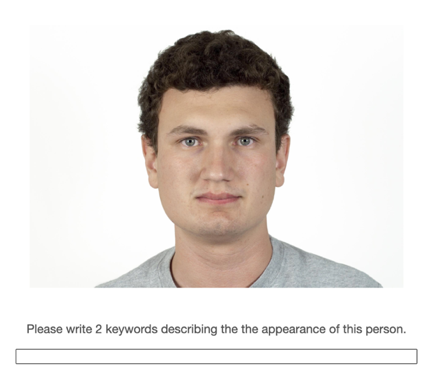 | This person provided food for flood victims. This person kicked their dog for eating cheese off the edge of the table. |

###

### ***Full List of Learn Deck Stimuli***

**Table S4.** Table of all revealed stimuli for Experiment 3.

| Morally Good | Moral Ambiguity | Morally Bad |
| --- | --- | --- |
| Christopher Anderson  66% Good  Daniel Thomas  68% Good  Paul Jackson  64% Good  Mark White  68% Good  Donald Harris  65% Good  George Martin  64% Good  Kenneth Thompson  69% Good  Steven Garcia  66% Good  Edward Martinez  64% Good  Brian Robinson  71% Good | James Smith  77% Ambiguous  John Williams  69% Ambiguous  Robert Johnson  62% Ambiguous  Michael Jones  77% Ambiguous  William Brown  74% Ambiguous  David Miller  62% Ambiguous  Richard Davis  80% Ambiguous  Charles Wilson  67% Ambiguous  Joseph Moore  67% Ambiguous  Thomas Taylor  71% Ambiguous | Ronald Clark  64% Bad  Anthony Rodriguez  52% Bad  Kevin Lewis  67% Bad  Jason Lee  64% Bad  Matthew Walker  65% Bad  Gary Hall  55% Bad  Jose Young  70% Bad  Larry Wright  66% Bad  Geoffrey King  78% Bad  Jerry Allen  61% Bad |

## **Experiment 4**

### ***Instructions***

We are interested in finding out about what kinds of things make you most curious.

To better understand what makes people curious, we will ask you to select between two different people in this task.

To make your decision, we will show you the name of each of these people, and an accompanying “**ambiguity** **score**”. This score is calculated from other people's ratings about the mind/thoughts of a person or from ratings about the works of art created by a person (with their real names changed to pseudonyms).

The ambiguity score you will see for each person is an average calculated from the responses of all the participants in that previous study.

This means that on each trial of the task, you will see an option to select a person who was rated **morally** (which means concerned with right and wrong) **ambiguous**, and an option to select a person who created works of art that were rated as **aesthetically** (which means concerned with beauty or the appreciation of beauty) **ambiguous**.

Your task is to choose which person you'd like to learn more about based on their ambiguity score.

To make your decision, try to imagine the actions or the works of art that may have prompted others to rate the person as they did. Let yourself get caught up in imagining what the person feels, how they think, and what their motives or reasons for acting or for their art probably are.

After you make a selection, you will answer some questions and then get to learn about why the individual was rated that way.

The goal of the task is to select the person who is **most fascinating** to you on each trial.

You are free to choose whatever kind of person on any trial, and are free to move from person to person. If one kind of person begins to seem preferable, feel free to choose that category more often. You will be shown a short paragraph about the selected person, regardless of the option selected. It is only the kind of information you will read about that differs.

Overall, this task will take the same amount of time regardless of which type of person you choose.

### ***Full List of Vignette Stimuli***

**Table S5.** Table of all revealed stimuli for Experiment 4.

| Aesthetic Ambiguity | Moral Ambiguity |
| --- | --- |
| This person's work is known to be pleasant but also derivative. The artist uses color and shapes in a way that is visually appealing but that also closely resembles the work of other painters. There is a beauty to his work, but it does not look wholly original. Most typical art observers think this work is nice, but critics find the work unimaginative. | This person often engages in foolish and playful behavior that may bring about chaos. However, he met a new group of friends that motivates him to do better and pulls him away from engaging in this kind of behavior. But he still enjoys being foolish and is foolish often. He is driven by his selfish impulses but is learning to control them. |
| This person's work is technically impressive but it lacks depth. The artist focuses on demonstrating their skill more than they focus on conveying a deeper message or feeling. Critics have said many complimentary things about the technique, but the lack of a message makes this work feel flat. Average art observers find the color use pleasing, but critics largely find the work uninspired. | This person often has good intentions, but his passionate nature gets in the way. As a result, he often causes trouble for and harm to others without intending to do so nor realizing it entirely. He is constantly driven to seek risky, adrenaline inducing situations, but he is also internally motivated by being seen as good. |
| This person's work is known to be harmonious but also unsuccessful in its use of symbolism. The artist uses shapes and textures to create a balanced piece but the work is also lacking in its use of clearly powerful symbols. This artist's work has the right balance, but it doesn't quite convey the depth of human experience. Most typical art observers think this work is boring, but critics find the work technically skilled. | This person is successful in their career and is charismatic. To get to where he is, he had to tear down a lot of people. He now mentors individuals he works with to make them more successful. He is driven by a desire to reach his goals and be well liked, but he is willing to make the tough decisions to prioritize his success over that of others. |
| This person's work is known to use discordant colors but also lacks clarity of intent. The artist uses colors that don't usually go well together in a way that is visually interesting but that also lacks direction or substance. There is a potential in this work, but it does not have clear artist intent, making the piece look sloppy. Most typical art observers think this work is unappealing, though critics find the work inventive. | This person is manipulative. He uses deception to control others, but the nature of his work requires this skill. He often uses manipulation and deception to keep his children and family safe from people that want to take harm them. He is driven by a desire to protect his family but can't help but use his skills of manipulation to beat out others at work. |
| This person's work is known to be emotional but lacking in technical ability. The artist uses wild brush strokes to create an intense feeling, but does not match the skill that other professional painters have, even in a similar genre. It is emotional but does not look professional. Most typical art observers think this work is powerful, but critics find the work too amateur. | This person can be messy and short-tempered. While he is nice deep down, he tends to hide this characteristic from others, sometimes being callous or mean. He puts on a tough persona so others will keep their distance and don't pose a threat to him. He is driven by a desire to protect himself from harm but at his core he is kind and vulnerable. |
| This person's work is known to create a sense of awe but the identity of the artist is not well conveyed. The artist work is large-scale, but does not seem to come from any particular perspective. There is strong technique in this work, but it lacks message. Most typical art observers think this work is fun to look at, but critics find the work lacking in substance. | This person is aggressive, demanding, and impatient. However, when he is with his friends and family, he acts warm and kind to them. He loves animals and takes care to never hurt his those close to him, putting their needs above everyone else. When around strangers he is often volatile and mean. He is driven to protect those close to him at any cost. |
| This person's work is known to be fun but also conventional. The artist crafts simple images using common and easily identifiable shapes along with cohesive color schemes. But this work also lacks originality. There is a simplicity to this work that makes it easy to understand, but it does not have an imaginative element. Most typical art observers think this work is inviting, but critics find the work boring. | This person is a teacher who is very harsh. He assigns many assignments and is a strict grader. However, he does this out of love for the development of his students. He wants his students to learn and succeed in life. He is harsh and has a difficult personality, but he is driven by a desire to lift his students up because he believes in them. |
| This person's work is known to be lack in color and style but also well connected to elements from the rest of the art canon. The artist uses very few colors and symbols, but they engage with the larger culture of art. There is a lack of a wow factor to this work, but it is well contextualized. Most typical art observers think this work is unfun, but critics find it evocative. | This person is hungry for wealth and for influence. While he is very self-interested in many instances, he is also the kind of person who will go to great lengths for his loved ones. He feels invigorated by gaining influence at work but is always fair when dealing with others. He is driven by his competitive nature but being fair is also important to him. |
| This person's work is known to be stimulating but lacking in its conceptual meaning. The artist uses color in a way that is visually alluring but does not convey a message or demonstrate technical skill. There is something absorbing about this work, but it does not have a clear message or the skill to convey it. Most typical art observers think this work is entertaining, but critics find the work vapid. | This person is in the process of bettering themselves. However, at the same time, he continues to engage in actions that are bully-like. These actions often hurt those closest to them. He is struggling to balance feeling authentic and becoming a better person. He is driven by a desire to be enjoy his true darker desires but also to be kind to those he loves. |
| This person's work is known to be clean and minimalist but also bland. The artist uses color and sharp lines to craft an image that is simple and austere, but the work fails to do justice to the prior work that inspired it. There is a beauty to this work, but it does not forge connections. Most typical art observers think this work is dull, but critics find the work aesthetically pleasing. | This person often finds himself engaging in behaviors that are not seen as good, like stealing from others. However, he does so to serve disadvantaged people. He does not have the ability to help on his own, so he is willing to take from others and give to those with little. He is driven by a desire to see fairness among people but also to achieve that in his own ruthless way. |

# **Pilot Experiment: Exploring curiosity for fictional characters**

Themes around good and evil permeate throughout many narratives in life. From the stories we engage in fiction and in religious texts to the judgments that we make about others. The morality of characters in fiction and entertainment represents a crystallization of the powerful moral norms in our modern society. In films, video games, and more, the plot often centers around a character that fights with and makes salient the moral norms. They do this through breaking some norms and upholding others, and their motives thus seem complicated.

We set out to investigate whether people prefer to learn about fictional characters with complicated moral pasts compared to characters that are either morally good or morally bad. In a pilot study, we pit famous fictional antiheroes and villains against heroes from TV and movies. This approach builds on work in media psychology that suggests morally ambiguous characters are highly identifiable with the self (e.g., Krakowiak & Tsay-Vogel, 2015; Tsay & Krakowiak, 2011), and research that suggest moral badness piques interest (Bloom 2021; Krause & Rucker, 2020). We hypothesized that the ambiguous characters and villains would be more interesting to learn about than the heroes.

### **Method**

**Design**

The pilot study used a fully within-in subjects design to examine the effect of fictional character type (antiheroes, heroes, or villains) on decision-making.

**Participants**

We collected data from 69 consenting participants on Prolific. The only inclusion criterion was current United States residence to ensure that participants were likely to be familiar with the fictional characters. Of these participants, 7 did not pass the task comprehension check items. As such, we report the subset of participants who completed all measures and passed task comprehension checks. We conducted our analyses on a final sample of 62 participants (*M*_age_ = 29.85, *SD*_age_ = 9.3, Male = 26, Female = 34, 2 = Other) who answered questions about at least one of three presented issues. All 62 of those participants passed self-reported attentiveness checks. Participants were paid $3.80 on average for their participation

**Materials**

***Information Seeking Task***

The pilot experiment tested whether moral ambiguity sparks curiosity for explanation-seeking (i.e., it is engaging) by assessing preferences for competing sources of information. We used a modified empathy task (Cameron et al., 2019) where participants were introduced to two playing card decks, one called the “Describe” deck and one called the “Learn” deck (see Figure S1). Participants were asked to select one of the decks on each trial. In the instructions portion of the experiment, participants were told about the kind of information available when selecting each of the decks, and they were told that they could select any deck on any trial and were free to change their minds often. The “Describe” deck yielded physical appearance related information and a single image of the character (i.e., factual information), and the “Learn” deck yielded information about the moral motives (i.e., explanation information) of that character with no image. That is, the descriptive information does not offer the same in-depth mind explanation for the character that the “Learn” deck did, thus allowing us to test whether morally ambiguous and morally bad minds more often elicit curiosity for explanations. We also assumed that descriptive information is generally more boring than motive information, and so we included a picture of the target in the describe deck only in order to create two closely matched options. On each trial, participants were shown the two decks to select from and a name of a familiar hero (e.g., Captain America), antihero (e.g., Severus Snape), or villain (e.g., Hannibal Lecter) in completely random order. For both decks, participants were also asked to write two keywords that describe the information they were shown and then answer questions about their interest in the character, and how good and bad the character is. This task was used to see whether individuals preferred to learn about the motives of morally ambiguous characters (vs. a description of their appearance).

***Character Stimuli***

We brainstormed a series of characters and selected 30 popular characters from TV and cinema who we thought best met the criteria of a hero (e.g., Superman), a villain (e.g., Lex Luthor), and an antihero (e.g., Han Solo; Eden et al., 2015; Krakowiak & Tsay-Vogel, 2015; Raney, 2004). We then had laboratory research assistants indicate their familiarity with each of the characters and selected the top 10 most familiar characters from each of the three categories. All name stimuli were presented in Arial 16-point font in all caps. The full list of selected characters is available on the project’s OSF page.

***Manipulation Checks***


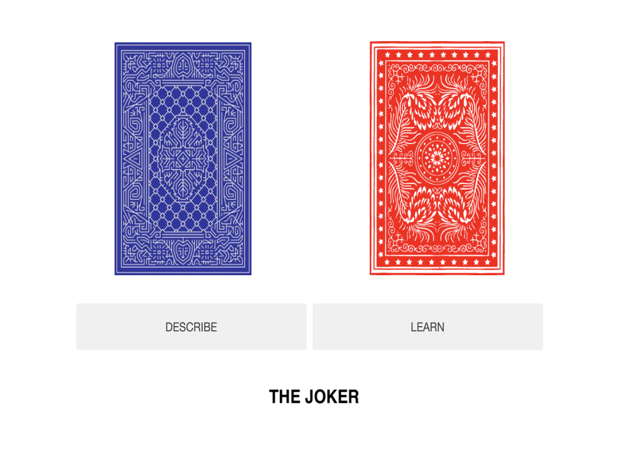
To ensure that we accurately categorized our selected characters used as antiheroes, heroes, or villains, we asked participants to answer two manipulation check items. Following each decision in the main experimental task, participants were asked “How morally good is this person?” and “How morally bad is this person?” measured on a scale from 1 = *Not at all* to 9 = *Extremely*.

**Figure S1.** Example of a single deck task trial with a villain name as the stimulus.

***Self-reported Interest***

As noted above, we also measured self-reported interest for each of the characters on a trial level. We asked participants “How interesting is this person?” on a trial level, again rated on a scale from 1 = *Not at all* to 9 = *Extremely*.

***Character Familiarity Checks***

At the end of the experiment, we also asked participants to indicate whether they were familiar with each of the fictional characters they were presented prior to this experiment. They were asked a binary “yes” or “no” for each name presented. This measure was used to ensure that results of the main task replicated with only the subset of participants who knew about the characters prior to the experiment. We did not conduct any additional analyses using this measure.

**Procedure**

Participants chose between the two decks: a “learn” deck that offered information about the motives and morals of the character, or a “describe” deck that offered an image and information about their appearance. On each randomly presented trial, participants saw the name of a character and the two decks to choose from. After selecting their deck, participants had to write in three words to describe the character and indicate their interest and complete manipulation checks for each character (i.e., on a trial level). A total of 30 characters, ten heroes, ten antiheroes, and ten villains were presented. Participants were then asked whether they recognized each character individually, asked about their attentiveness, and basic demographic information. Finally, participants were debriefed and paid for their participation.

### **Results**

Target analyses were run using all trials from participants and excluding participants who did not understand instructions (*N* = 7). However, the results remain mostly unchanged when we use only the subset of participants who were familiar with the characters they saw. When we subset only familiar character trials, all predicted patterns get stronger. As a stringent test, we include all trials here. These analyses use the ‘lme4’ package (Bates et al, 2015) and the ‘lmerTest’ package (Kuznetsova et al., 2017) to compute model *p*-values using R statistical analysis software (R Core Team, 2019). Each survey was distributed via Qualtrics survey software (Qualtrics, Provo, UT). Each of the target analyses uses linear mixed-effects models with participants modeled as random unless otherwise stated.

**Self-reported Interest**

When we entered Moral Agent Type as the predictor and self-reported interest as the outcome into a mixed-effects model, results suggested a significant difference among the three groups. Moral heroes were rated as more interesting than villains (*b*= −0.58, *SE* = 0.12, *t*(1179) = −4.81, *p* < .001, *r* = 0.14, 95% *CI* [-0. 82, -0.35]) and antiheroes (*b*= −0.30, *SE* = 0.12, *t*(1179) = −2.49, *p* = .013, *r* = 0.07, 95% *CI* [-0. 54, -0.06]).

**Interestingness of antiheroes**

We used a generalized mixed effects models with subjects and stimulus included as random effects to test whether morally ambiguous characters lead to more “learn” trials in the binary choice task. As predicted, participants selected the “Learn” deck significantly more for antiheroes compared to heroes, *b* = 0.35*, SE* = 0.17*, z* = 2.09*, p* = .037*, OR* = 1.45, *95% C*I [0.02, 0.69] (see Figure S2). They also selected the “Learn” deck significantly more for villains compared to heroes, *b* = 0.37, *SE* = 0.17, *z* = 2.18, *p* = .029, *r* = 0.10, *95% C*I [0.03, 0.70]. However, we found no significant differences between antiheroes and villains (*b* = 0.01, *p* =.94, *95% C*I [−0.34, 0.35]). These patterns remained robust when we included only participants who knew the characters in analyses (ambiguous: *b* = 0.42, *p* = .025; villains: *b* = 0.41, *p* = .028).

***
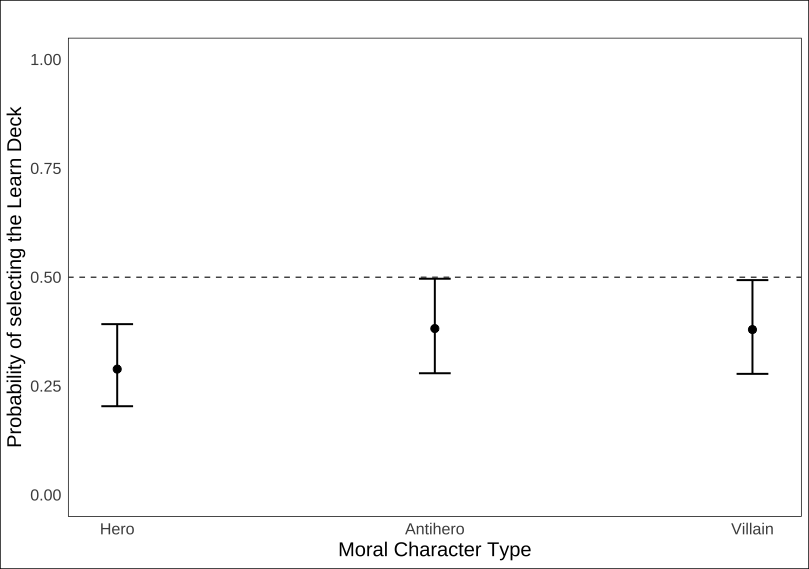
***

**Figure S2.** Predicted probabilities for selecting the “Learn” deck by character type. Error bars represent 95% Confidence intervals.

**Discussion**

In the pilot experiment, we found the predicted pattern of results: The “Learn” deck, which revealed moral information about the characters was greater for antiheroic characters compared to heroes. However, there was no significant difference between antiheroes and villains. It is noteworthy that ambiguity and badness are more closely linked and elicit more explanation-seeking than goodness. We take this difference as evidence that the basic assumption that people seek moral goodness is not always true (see also Pizarro & Baumeister, 2013).

Another important pattern to note is general pattern of low rates of “Learn” deck selection. There are a few reasons this pattern may have emerged. One reason is that the “Describe” deck offers an image of the fictional character, many of whom are famous, attractive actors. Participants may have been more inclined to see these familiar and attractive faces. This may have also contributed to the differences evidenced between good and the other two Moral Agent Types. The actors cast to play heroes tend to be conventionally attractive, which may have driven participants to select the “Describe” deck more often than the other two types. Another reason we might see the “Describe” deck emerge as the default is that this deck may be easier—reading factual information does not require a create deal of cognitive engagement. As such, it may be experienced as easier compared to the other deck. If this is the case, the selection of the morally bad and morally ambiguous characters is even more impressive—this interpretation implies that the curiosity to gain access to explanation information is strong enough to overcome the desire to find the easy way out in an online survey setting. Overall, this pilot provides preliminary evidence that antiheroes are more interesting than morally good characters but not more interesting than bad characters.

# **Additional Analyses**

## **Study 1**

**Correlations when including genre**

We ran an exploratory model that included genre/type of show as a covariate and one that included average show/movie length to test whether effort and morality remained correlated. When accounting for the show length the relationship between morality and effort (the negative relationship reported in the main text) remains robust, *b* = −0.06, *SE* = 0.02, *t*(3407) = −3.70, *p* < .001, *r* = −0.06. However, when accounting for the genre/type of show, the relationship between morality and effort is no longer present, *b* = -0.02, *SE* = 0.02, *t* = -1.09, *p* = 0.28.

**Top shows by hours viewed**

We conducted additional analyses to test the data from the five-month period. We split each Netflix TV show/movie into two groups—entertainment having morally good or morally protagonists. We then looked at the total hours viewed across each of the shows. Figure S3 depicts the total hours viewed broken down by moral categorization. This figure takes the top shows from the larger list of 133 shows. Notably, *The* *Witcher* was categorized as good by participants, but his character commits many acts of violence but only against “bad” guys in a fantasy world. This is an interesting future direction that fits well with previous research on the effects of genre on imaginative resistance (Liao et al., 2014). Violence perpetrated in a fantasy world may not engage the same cognitive processes as violence perpetrated in worlds that more closely resemble our own. Thus, the same moral wrongs that occur in *The* *Witcher* as do in *Ozark* are perceived differently because of the context. Ultimately, however, both of these top shows have characters who kill others, and people are largely drawn to engage with those shows.


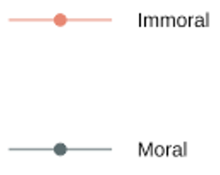

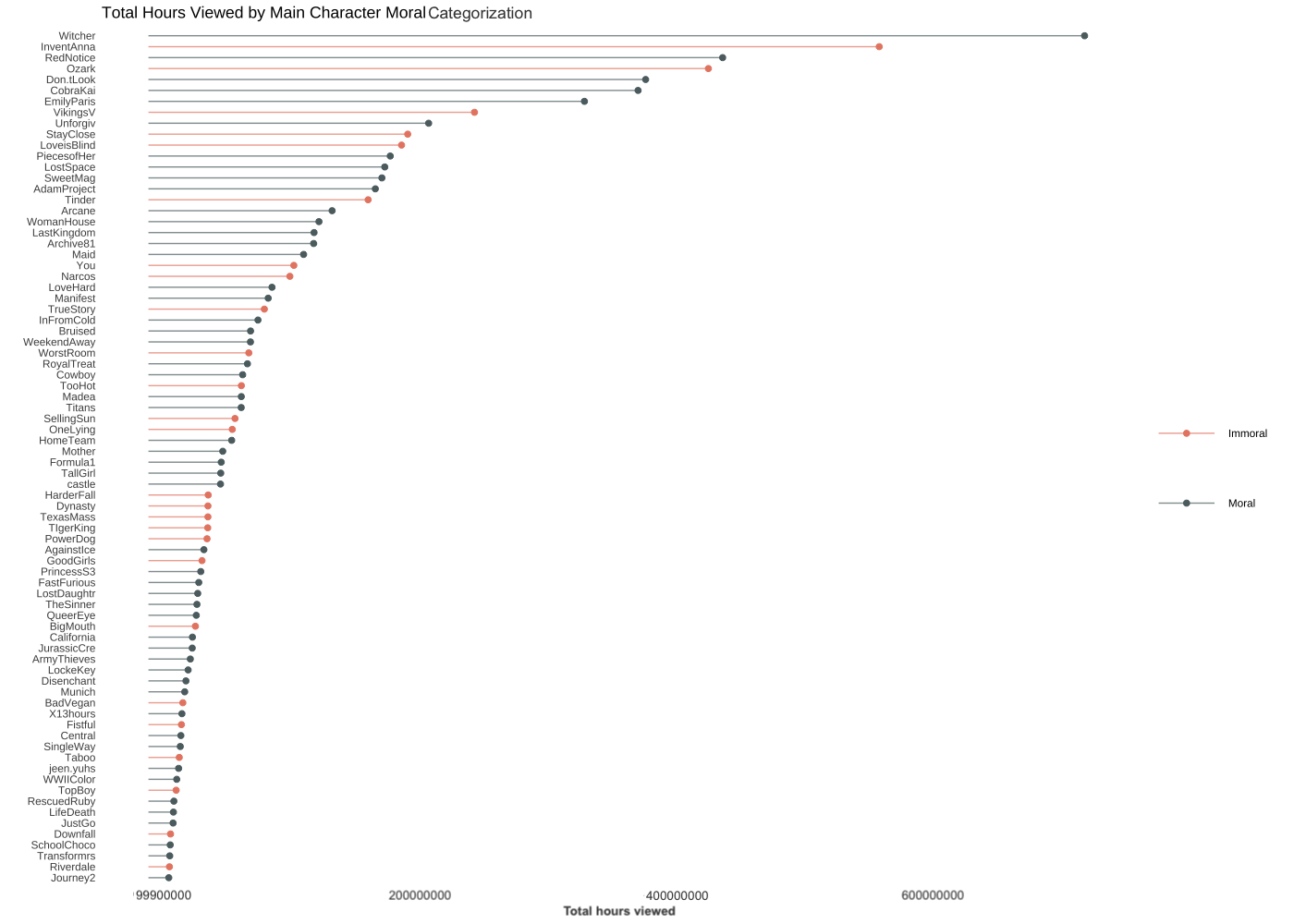


**Figure S3.** Total hours viewed for top half of Netflix shows/movies from a five-month period. Shorthand title names are used here. Full list of TV shows/movies available on OSF. During the window of investigation, the most watched show was Witcher followed by Inventing Anna.

**Bayesian approach to model fitting**

In order to conduct an analysis that included random effects for participants in Study 1, we fit a linear mixed effects model that includes all measured variables (morality of the protagonist, perception of learning, similarity, effort) as predictors and hours watched (target variable) as the outcome using the “rstanarm” package (Goodrich et al., 2020). This modelling strategy allowed us to estimate the parameters of interest with the desired random-effects structure. When we run this model, the estimates are similar to the linear model that does not include random-effects, which is reported in the main text. Critically, results again suggest that the morality of the protagonist negatively predicts hours watching (beta = -0.18, 95% CI [-0.23, -0.13]). We also found that learning (beta = 0.09, SE = .02, 95% CI [0.04, 0.14]) and effort (beta = 0.10, SE = .02, 95% CI [0.05, 0.14]) were significant positive predictors of hours spent watching. Together these results suggest the results using a less complex modeling approach are comparable.

## **Experiment 2a**

Here, we report any ancillary analyses that were preregistered for Experiment 2a but not included in the main text. We also included exploratory items to better understand the information-seeking task and report those patterns as well.

**Method**

**Self-reported Interest**

Participants answered questions about their self-reported interest at the trial level. Participants were asked, “How interesting do you think this person is?” rated on a scale from 1 = *Not at all interesting* to 7 = *Extremely interesting* to measure interest.

**Additional individual differences**

We report patterns of results only for a subset of the individual differences in the main text. As such, we report the findings from the Morbid Curiosity scale (Scrivner, 2021), Belief in a Just World (Dalbert, 1999), Perspective-taking Empathy (Davis, 1980) and short Need for Cognition scale (NFC-short; Lins de Holanda Coehlo et al., 2020).

**Exploratory items**

We asked participants three questions adapted from Ferguson et al. (2020). We asked, “To what extent were you trying to show that you had good ethics and values on the decision task (4 option decision) you just completed?” (Self-presentation), “To what degree did you believe that the decision task you just completed was a measure of moral character and values?” (Self-evaluation), and “To what extent does moral character [evaluation] involve feeling empathy for other people?” (Skill-evaluation). Each item was rated on a 7-point scale.

**Results**

**Moral Information Seeking Task: Time Series Analysis**

We tested whether phase one selections differed across time. To do this, we created a numerical version of the categorical moral agent decision outcome that was coded to go from least morally bad (good agents followed by average agents) to most morally bad (ambiguous agents followed by bad agents). We entered time as a continuous predictor and included by-participant random intercepts to predict choice. Results suggested that there was a small but significant effect of time on choices (*b* = −0.02, *SE* = 0.007, *t*(2744) = −3.18, *p* = .002, *r* = 0.06)—as the trials went on, people were more likely to select morally good agents compared to the beginning of the task, when people more frequently selected immoral agents (see Figure S4).

We also tested whether feelings of satisfaction changed over time (i.e., after gaining access to explanations). We did not find any statistically reliable effects.

**
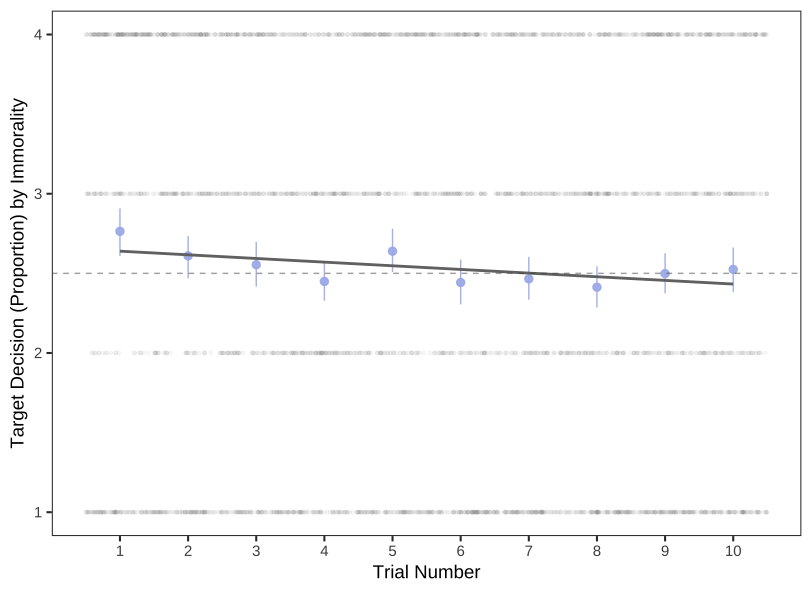
**

**Figure S4.** Average Moral Information Seeking Task decisions over time. The dots indicate the average across participants and the vertical lines indicate bootstrapped 95% Confidence Intervals.

**Self-reported Interest**

Phase one moral target selection predicted similar patterns for self-reported interest as with curiosity (*r* = 0.69, *p* < 0.001). Results suggested that participants reported more interest for ambiguous (*b* = 1.09, *SE* = 0.09, *t*(284) = 12.24, *p* < .001, *r* = 0.59, *95% CI* [0.91, 1.26]), bad (*b* = 1.49, *SE* = 0.11, *t*(367) = 13.75, *p* < .001, *r* = 0.58, *95% CI* [1.28, 1.71]), and good (*b* = 0.66, *SE* = 0.07, *t*(254) = 10.04, *p* < .001, *r* = 0.53, *95% CI* [0.53, 0.79]) compared to average moral targets. Like the patterns from curiosity judgments, the ambiguous and bad targets were rated as most interesting.

**Additional Models**

We ran additional models to examine the robustness of the effect of moral agent selection on curiosity. When we included each of the Phase 2 judgments (confidence, expected learning about human nature, similarity) and the interaction term between confidence and moral agent selection, the patterns of results for curiosity change slightly. While the effects of selecting a morally ambiguous agent (b = 1.26, SE = 0.18, t(920) = 6.94, p < .001, r = 0.22) and a morally bad (b = 0.98, SE = 0.19, t(1094) = 5.20, p < .001, r = 0.16) remain statistically significant—people report more curiosity for morally ambiguous and morally bad targets relative to average ones—morally good targets are no longer statistically different from morally averages ones (b = 0.24, SE = 0.13, t(1291) = 1.78, p = .075, r = 0.05). Further, the interaction between the ambiguous agent and confidence was statistically significant, b = −0.11, SE = 0.04, t(1221) = −2.51, p = .012, r = 0.07. Relative to the morally average targets, the more confident people reported being about the morally ambiguous targets, the less they reported being curious about them. There were no other statistically significant interactions. Full model results are reported in Table S6.

**Additional individual differences**

Overall, patterns were similar across additional moderators such that significant interactions were ordinal. All models reported below include a moderator, phase one target selection, and their interaction term as predictors of curiosity. Random intercepts and slopes are included for all models.

***Belief in a Just World***

There was a marginal main effect of belief in a Just World on curiosity, *b* = 0.18, *SE* = 0.09, *t*(280) = 1.98, *p* = .049, *r* = 0.12 (see Figure S5). The interaction terms were not significant. Relative to the average moral agent target, only morally ambiguous approach approached significance, *b* = −0.16, *SE* = 0.09, *t(*268) = −1.83, *p* = .068, *r* = 0.11. People high in belief in a just world were slightly less curious about ambiguous targets, potentially avoiding the non-worldview confirming information.

**
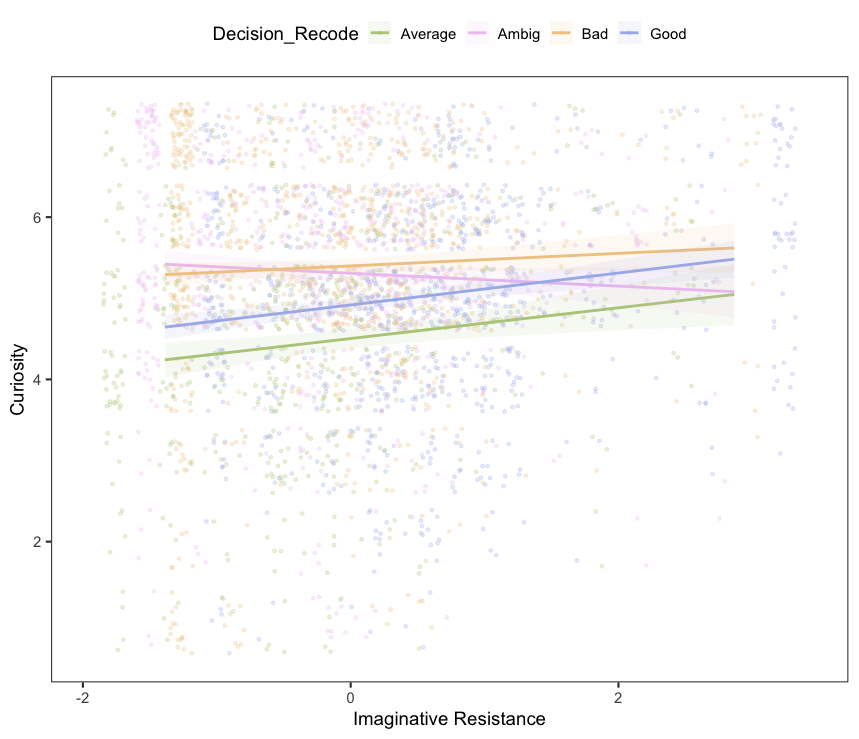
**


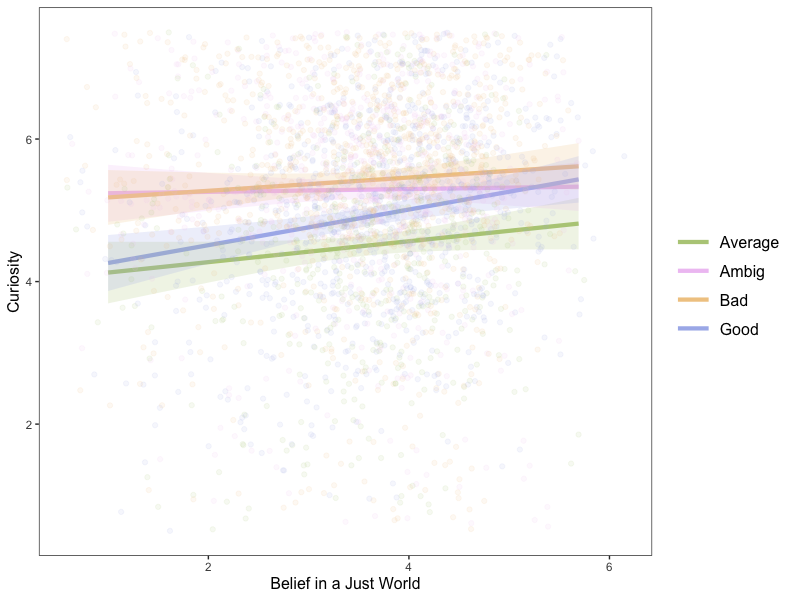


**Figure S5.** Individual differences in Belief in a Just World predicting curiosity for each of the phase one moral agent type decisions. Shading represents 95% Confidence Intervals.

***Perspective taking***

Perspective taking was a significant predictor of curiosity, *b* = 0.28, *SE* = 0.05, *t*(274) = 5.57, *p* < .001, *r* = 0.32 (see Figure S6). Additionally, relative to morally average targets, the interaction between perspective-taking and morally ambiguous, morally good, and morally bad were all statistically significant (ambiguous: *b* = −0.18, *SE* = 0.05, *t*(260) = −3.50, *p* < .001, *r* = 0.21; bad: *b* = −0.11, *SE* = 0.05, *t*(259) = −2.09, *p* = .038, *r* = 0.13; good: *b* = −0.09, *SE* = 0.04, *t*(244) = −2.27, *p* = .024, *r* = 0.14). For those high in perspective-taking, curiosity was highest for the non-average targets.

**
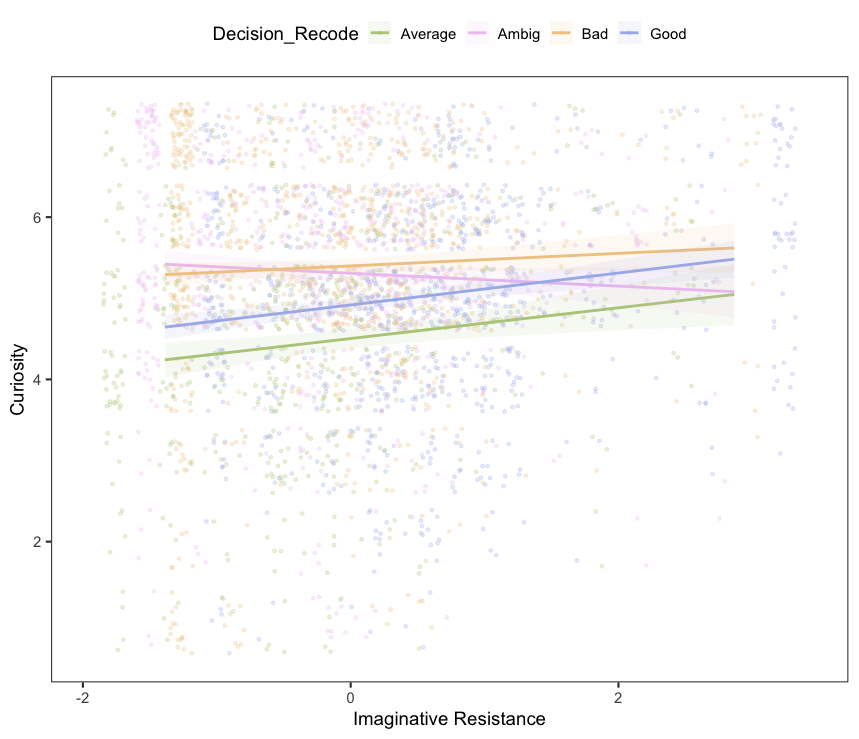
**


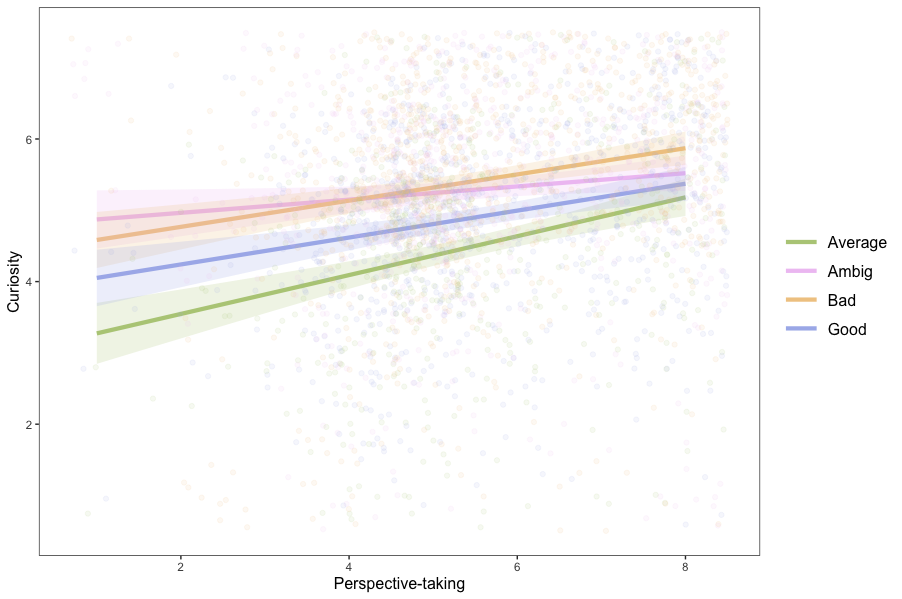


**Figure S6**. Individual differences in Perspective-taking predicting curiosity for each of the phase one moral agent type decisions. Shading represents 95% Confidence Intervals.

***Morbid curiosity***

For morbid curiosity, we again saw that the individual difference predicted more curiosity, *b* = 0.18, *SE* = 0.06, *t*(600) = 3.04, *p* = .003, *r* = 0.12 (see Figure S7). There was also a significant interaction between morbid curiosity and the morally bad target relative to morally average, *b* = 0.13, *SE* = 0.05, *t*(2762) = 2.82, *p* = .005, *r* = 0.05. Morally bad targets were most interesting to those high in morbid curiosity.

**
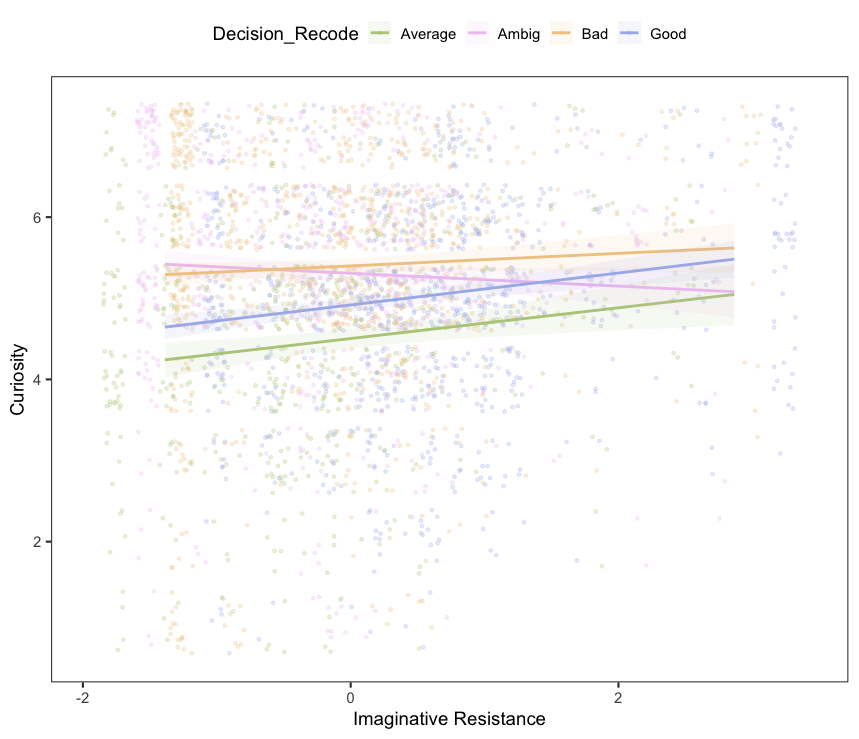
**


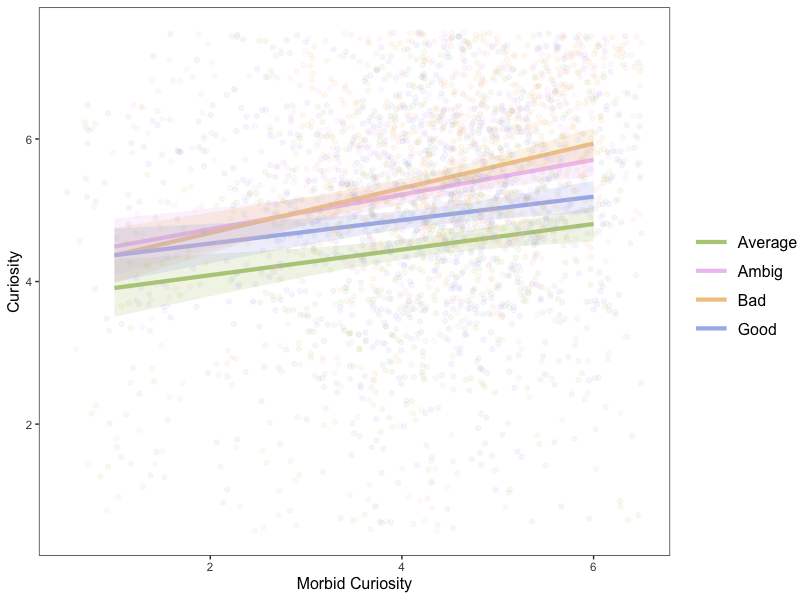


**Figure S7.** Individual differences in Morbid Curiosity predicting curiosity for each of the phase one moral agent type decisions. Shading represents 95% Confidence Intervals.

***Need for Cognition***

NFC was also associated with increases in curiosity, *b* = 0.22, *SE* = 0.07, *t*(621) = 2.95, *p* = .003, *r* = 0.12. We did not find any significant interactions, suggesting that need for cognition contributes to general curiosity for more information, rather than specific worldview related information.

**Exploratory items**

We also included three additional exploratory items to better understand the motives that might interact with curiosity in the experimental setting. For the following items, we use linear mixed-effects models that specify random-intercepts for participant.

**Curiosity**. The self-presentation and the skill-evaluation items predicted curiosity. People who used their task decisions to illustrate their own moral values reported greater curiosity, *b* = 0.06, *SE* = 0.03, *t*(303) = 2.06, *p* = .040, *r* = 0.12. Similarly, those who believed the task required empathy also reported more curiosity, *b* = 0.16, *SE* = 0.05, t(303) = 3.38, *p* < .001*, r* = 0.19. The self-evaluation item did not predict curiosity, *p* = .503.

**Perceived Learning.** We also examined how these items predicted perceptions of learning. We found that both self-items predicted greater perceptions of utility of learning. People who reported that task decisions show their own moral values reported greater learning utility, *b* = 0.19, *SE* = 0.04, *t*(303) = 4.85, *p* < .001, *r* = 0.27. Similarly, those who felt that the task measured their own morals also reported greater learning utility, *b* = 0.16, *SE* = 0.04, *t*(303) = 4.06, *p* < .001, *r* = 0.23. There was no relationship between task empathy and learning utility, *p* = .183.

For the learning about patterns questions, results mirrored the learning utility item relationships. People who reported that task decisions show their moral values reported learning more about a genuine pattern, *b* = 0.10, *SE* = 0.03, *t*(303) = 3.84, *p* < .001, *r* = 0.22. Similarly, those who felt that the task measured their own morals also reported learning more about a genuine pattern, *b* = 0.09, *SE* = 0.03, *t*(303) = 3.07, *p* = .002, *r* = 0.17. There was no relationship between task empathy and pattern learning, *p* = .300.

Lastly, we investigated the broadness of applicability item. For this item, only the self-presentation item was a significant predictor, *b* = 0.09, *SE* = 0.03, *t*(303) = 3.60, *p* < .001, *r* = 0.20. The two others were not statistically significant, *p*’s > .29.

Overall, we found that how participants approached the task had an influence on their curiosity and learning. This was especially true for the perceived utility of learning—those who felt the task was related to real life morality reported more perceived learning than those who did not.

## **Experiment 2b**

Experiment 2b was nearly identical to Experiment 2a with a few exceptions. First, this experiment reduced the choice set from four moral agents to a random selection of two moral agents (for the list of four). This had minimal effect on selection patterns, which are discussed in the main text. Next, Experiment 2b used a slightly different memory task, which is also discussed in detail in the main text. For the rest of the analyses, the patterns of results are nearly identical and so we report them here. Any instance where the patterns deviate has been noted in the main text and in this supplement. Additionally, as in Experiment 2a, we again include ancillary, preregistered analyses here. We also include an exploratory analysis of moral information task decisions over time.

**Method**

**Self-reported Interest**

We measured interest the same as in Experiment 2a

**Additional individual differences**

Following the analysis strategy of Experiment 2a, we report patterns of results for morbid curiosity, need for cognition, belief in a just world, and perspective-taking empathy here.

**Results**

**Moral Information Seeking Task: Time Series Analysis**

We tested whether phase one selections differed across time for the different choice sets. To do this, we collapsed choice sets that contained the same two moral agents into a single set, regardless of the order that the agent was presented in (i.e., a choice set that presented a bad agent on the left and a good agent on the right and a choice set that presented a good agent on the left and a bad agent on the right were collapsed into a single good vs. bad choice set). This left us with six total choice sets: Average vs. Ambiguous, Average vs. Good, Average vs. Bad, Ambiguous vs. Bad, Ambiguous vs. Good, and Good vs. Bad. We then recoded the data to reflect our predictions—that people would choose immoral and nonnormative agents more frequently than moral or typical ones. For example, when the choice set was Average vs. Ambiguous, a 1 reflected the ambiguous agent and a 0 reflected the average agent (Average = 0 vs. Good = 1, Average = 0 vs. Bad = 1, Ambiguous = 0 vs. Bad = 1, Ambiguous = 1 vs. Good = 0, and Good = 0 vs. Bad = 1). When looking at the means, we found that a preference for immoral and atypical agents emerged (where the means are closer to 1 than to 0): when compared to average agents, people selected ambiguous (*M* = 0.613, *SD* = 0.487), good (*M* = 0.673, *SD* = 0.469), and bad (*M* = 0.618, *SD* = 0.486) more frequently. Looking to comparisons among agents matched on immorality or atypicality, we found that patterns over time mirror the main findings. While atypicality drove curiosity, preferences for atypical and immoral targets was greatest. People selected bad more than ambiguous (*M* = 0.565, *SD* = 0.496), good more than ambigious (*M* = 0.454, *SD* = 0.498), and bad more than good (*M* = 0.540, *SD* = 0.499). Patterns of results are depicted in Figure S8.

We also tested whether averaged decision patterns differed statistically from random choice selection. That is, we tested whether average selection over time revealed a preference for one target over the other, or if people used a random selection strategy for each of the choice sets. For Average vs. Ambiguous choice sets, there was a statistically significant difference from 50%, *t*(506) = 7.2059, p< .001, such that people preferred ambiguous agents. For Average vs. Bad, results statistically differed from 50%, *t*(497) = 7.2752, p < .001, such that people preferred bad agents to average ones. The same pattern emerged for Average vs. Good, t(492) = 10.587, p <.001 such that people preferred good agents to average ones. When comparing immoral agents Bad vs. Ambiguous, people preferred bad agents, a pattern which differend again from 50%, *t*(503) = 3.9443, p < .001. Preferences for good agents emerged for Good vs. Ambiguous and were statistically different from 50%, *t*(478) = -2.295, p = 0.022. Lastly, when comparing Good vs. Bad, the patterns again statistically differed from 50%, *t*(492) = 2.4911, p = 0.013 and revealed a preference for bad agents relative to good ones.


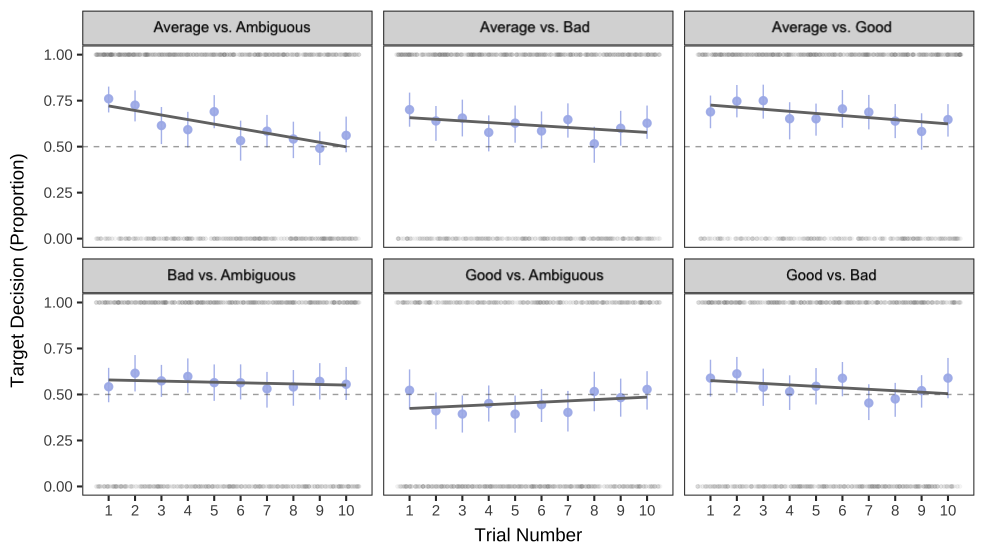


**Figure S8***.* *Moral Information Seeking Task decisions over time. The dots reflect the mean pooled across participants, and the vertical lines represent bootstrapped 95% Confidence Intervals. The data have been coded as follows: Average = 0 vs. Ambiguous = 1, Average = 0 vs. Good = 1, Average = 0 vs. Bad = 1, Ambiguous = 0 vs. Bad = 1, Ambiguous = 1 vs. Good = 0, and Good = 0 vs. Bad = 1.*

**Self-reported Interest**

Phase one selection predicted similar patterns for self-reported interest as with curiosity (*r* = 0.69, *p* < 0.001). Results suggested that participants reported more interest for ambiguous (*b* = 1.02, *SE* = 0.06, *t*(579) = 17.82, *p* < .001, *r* = 0.60, *95% CI* [0.91, 1.13]), bad (*b* = 1.68, *SE* = 0.07, *t*(794) = 22.76, *p* < .001, *r* = 0.63, *95% CI* [1.53, 1.83]), and good (*b* = 0.84, *SE* = 0.05, *t*(553) = 15.70, *p* < .001, *r* = 0.56, *95% CI* [0.74, 0.95]) compared to average moral targets. Again, mirroring the curiosity findings, interest was highest for ambiguous and bad moral targets.

**Additional Models**

We again conducted an exploratory analysis that included all Phase 1 and Phase 2 predictors to examine whether moral agent selection was a robust predictor of curiosity. The pattens of results for Experiment 2b were similar to those of 2a. Only the effect of selecting morally good agents was no longer significant when including the interaction term, *b* = 0.24, *SE* = 0.13, *t*(1291) = 1.78, *p* = .075, *r* = 0.05. However, for Experiment 2b, the only statistically significant interaction was between morally bad agents and confidence, *b* = 0.09, *SE* = 0.03, t(1946) = 3.11, *p* = .002, *r* = 0.07. Here, the more people reported being confident about morally bad agents, the more curious they were to learn about them. Full model results are reported in Table S6.

**Table S6.** Estimates for each predictor from Phase 1 and Phase 2 predicting Curiosity.

| Curiosity | | | | | | |  |
| --- | --- | --- | --- | --- | --- | --- | --- |
|  | Experiment 2a | | | Experiment 2b | | | |
| Variable | Beta | 95% CI^1^ | p-value | Beta | 95% CI^1^ | p-value | |
| **Moral Agent Selection** |  |  | **<0.001** |  |  | **<0.001** | |
| *Average (reference)* | — | — |  | — | — |  | |
| *Ambiguous* | 1.3 | 0.90, 1.6 |  | 0.70 | 0.46, 0.95 |  | |
| *Bad* | 0.98 | 0.61, 1.3 |  | 0.72 | 0.47, 0.98 |  | |
| *Good* | 0.22 | -0.15, 0.60 |  | 0.24 | -0.02, 0.50 |  | |
| **Confidence** | 0.09 | 0.02, 0.16 | **0.013** | 0.01 | -0.03, 0.06 | 0.58 | |
| **Expected Learning about Human Nature** | 0.29 | 0.26, 0.32 | **<0.001** | 0.37 | 0.35, 0.40 | **<0.001** | |
| **Similarity** | 0.06 | 0.03, 0.10 | **<0.001** | 0.05 | 0.02, 0.07 | **<0.001** | |
| **Moral Agent * Confidence** |  |  | **<0.001** |  |  | **<0.001** | |
| *Ambiguous * Confident* | -0.11 | -0.19, -0.02 |  | -0.01 | -0.07, 0.05 |  | |
| *Bad * Confident* | 0.02 | -0.07, 0.10 |  | 0.09 | 0.03, 0.15 |  | |
| *Good * Confident* | 0.02 | -0.06, 0.10 |  | 0.04 | -0.02, 0.10 |  | |
| ^1^CI = Confidence Interval | | | | | | |  |

**Phase Two: Expected information judgments**

***Curiosity & Confidence***

Confidence and curiosity were again moderately positively corrected, *r* = .25, *p* < 0.001 (Holm correction). For curiosity, results mirrored the patterns from Experiments 2a. Participants reported more curiosity for ambiguous (*b* = 0.74, *SE* = 0.05, *t*(539) = 13.84, *p* < .001, *r* = 0.51, *95% CI* [0.64, 0.85]), bad (*b* = 1.43, *SE* = 0.06, *t*(820) = 22.46, *p* < .001, *r* = 0.62, *95% CI* [1.31, 1.56]), and good (*b* = 0.55, *SE* = 0.05, *t*(536) = 11.16, *p* < .001, *r* = 0.43, *95% CI* [0.45, 0.65]) moral targets than for average targets. Overall, participants reported more curiosity for the ambiguous and the bad Moral Target Types.

As in Experiments 2a, participants again reported significantly less confidence for ambiguous targets (*b* = −0.54, *SE* = 0.05, *t*(578) = −10.26, *p* < .001, *r* = 0.39, *95% CI* [-0.65, -0.44]) than average targets. Compared to morally average targets, participants reported more confidence about bad moral targets (*b* = 0.60, *SE* = 0.06, *t*(878) = 9.92, *p* < .001, *r* = 0.32, *95% CI* [0.48, 0.72]), and good ones (*b* = 0.44, *SE* = 0.04, *t*(531) = 9.85, *p* < .001, *r* = 0.39, *95% CI* [0.35, 0.53]) (see Figure 7). Confidence was again lowest for ambiguous targets compared to all other moral targets.


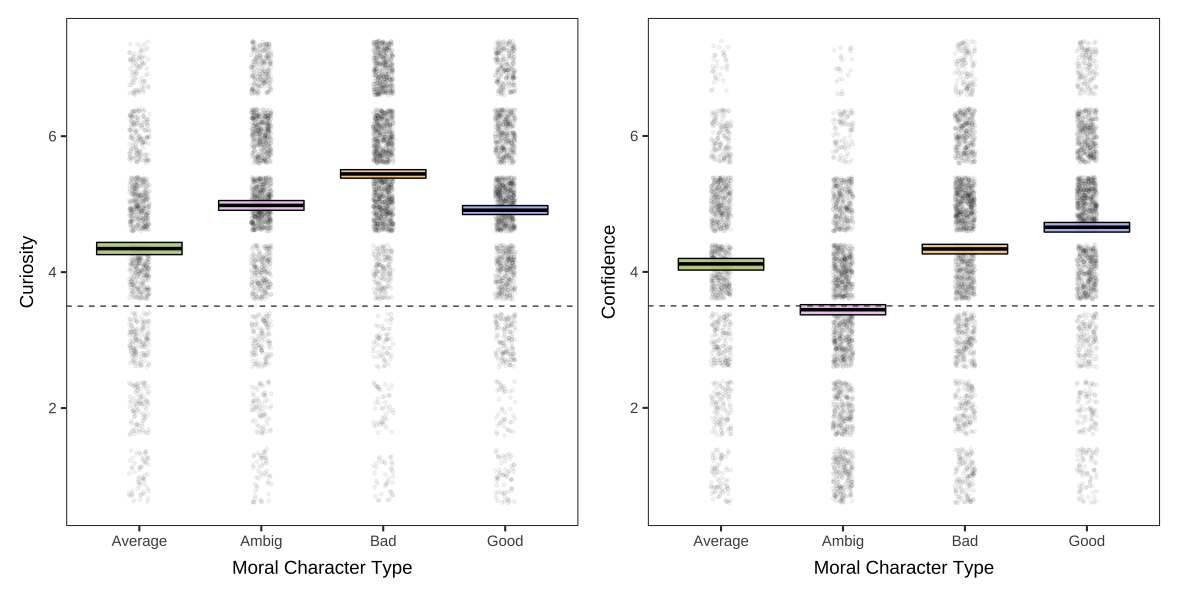


Good

Bad

Ambiguous

Average

Good

Bad

Average

Ambiguous

**Figure S9.** Patterns of results for the curiosity and confidence ratings. The thick black line in the boxes represents the mean, and the edges represent mean standard error from bootstrap.

***Expected Learning about Human Nature***

We also asked participants to indicate whether they expected to learn something about the selected target. Results yielded similar patterns to interest. Participants reported more expected learning about human nature for ambiguous (*b* = 0.24, *SE* = 0.05, *t*(516) = 4.95, *p* < .001, *r* = 0.21, *95% CI* [0.14, 0.33]), bad (*b* = 0.84, *SE* = 0.07, *t*(815) = 12.68, *p* < .001, *r* = 0.41, *95% CI* [0.71, 0.97]), and good (*b* = 0.32, *SE* = 0.05, *t*(549) = 5.92, *p* < .001, *r* = 0.24, *95% CI* [0.21, 0.43]) compared to average moral targets. Compared to morally average targets, expected learning about human nature was highest for bad targets, followed by morally ambiguous and good targets.

**Phase Three: Revealed information judgments**

***Normality***

Next, we tested normality judgments for the individual moral status targets as a manipulation check. Compared to morally average, ambiguous moral targets, *b* = −1.52, *SE* = 0.06, *t*(606) = −25.83, *p* < .001, *r* = 0.72, *95% CI* [-1.64, -1.41], morally bad targets, *b* −2.56, *SE* = 0.07, *t*(844) = −35.43, p < .001, *r* = 0.77, *95% CI* [-2.70, -2.42], and morally good targets, *b* = −2.07, *SE* = 0.07, *t*(598) = −30.99, *p* < .001, *r* = 0.79, *95% CI* [-2.20, -1.94], were rated as less average. When we included curiosity and its interactions term, results mirrored Experiment 2a. Curiosity was not a significant predictor and there were no significant interactions, *p*’s > .08.

For ideal judgments, patterns again mirrored Experiments 2a. Participants rated morally ambiguous targets, *b* = −0.62, SE = 0.05, *t*(573) = −13.03, *p* < .001, *r* = 0.48, *95% CI* [-0.71, -0.53], and morally bad targets, *b* = −2.21, *SE* = 0.05, *t*(1284) = −42.09, *p* < .001, *r* = 0.76, *95% CI* [-2.32, -2.11], as less ideal than morally average ones. Morally good targets were rated significantly more ideal, *b* = 1.67, *SE* = 0.05, *t*(614) = 33.01, *p* < .001, *r* = 0.80, *95% CI* [1.57, 1.77] than morally average (see Figure 8). Normativity patterns mirrored those of Experiment 2a. While morally average was seen as most average, ideal judgments for the morally good and bad targets were most extreme.


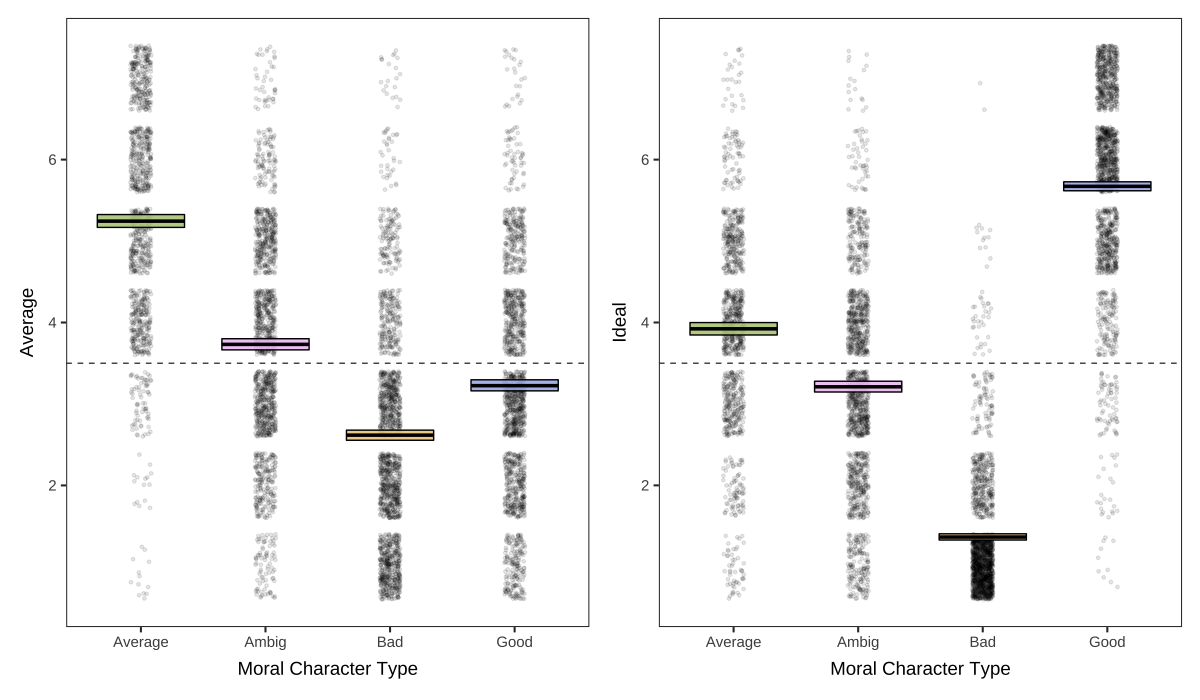


Good

Average

Ambiguous

Bad

Good

Bad

Ambiguous

Average

**Figure S10.** Patterns of results for the average and ideal ratings in Experiment 2b. The thick black line in the boxes represents the mean, and the edges represent mean standard error from bootstrap.

***Satisfaction***

We first tested whether satisfaction at phase three differed as a function of phase one moral target selection. Results suggested that satisfaction of the explanation reveal during the third phase of the trial was higher for the ambiguous, *b* = 0.37, *SE* = 0.06, *t*(565) = 5.97, *p* < .001, *r* = 0.24, *95% CI* [0.25, 0.49], and the good targets, *b* = 1.03, *SE* = 0.06, *t*(563) = 16.81, *p* < .001, *r* = 0.58, *95% CI* [0.90, 1.14], compared to the average targets. Satisfaction was only marginally lower for the bad moral targets than the average targets, *b* = −0.15, SE = 0.08, *t*(870) = −1.96, *p* = .050, *r* = 0.07, *95% CI* [-0.31, 0.001]. We also tested whether curiosity predicted satisfaction and again, curiosity was a statistically significant predictor of satisfaction, *b* = 0.27, *SE* = 0.03, *t*(1173) = 10.80, *p* < .001, *r* = 0.30, *95% CI* [0.24, 0.30]. Satisfaction was again highest for morally good targets. We again tested whether satisfaction changed over time, but did not find any statistically reliable effects.

***Perceived Learning***

Curiosity again predicted perceived utility of learning, *b* = 0.27, SE = 0.03, t(1173) = 10.80, p < .001, r = 0.30, *95% CI* [0.22, 0.33], such that more curiosity predicted more learning utility in phase three. The relationship between curiosity and learning utility was moderated by moral target. Morally good targets did not differ from morally average in magnitude of relation, p = .895, but both morally ambiguous moral targets, *b* = −0.09, SE = 0.03, t(1318) = −2.86, p = .004, r = 0.08, *95% CI* [-0.16, -0.03], and bad ones, *b* = −0.11, SE = 0.04, t(2169) = −2.98, p = .003, r = 0.06, *95% CI* [-0.18, -0.03], showed an attenuated relationship between curiosity and perceived learning. Moral curiosity predicted perceived utility, a pattern which was reduced for morally bad and ambiguous targets.

Looking to learning about real-life patterns, results suggested that curiosity again predicted learning, *b* = 0.17, *SE* = 0.03, *t*(1306) = 5.80, *p* < .001, *r* = 0.16, [0.11, 0.23]. However, curiosity did not predict broadness of learning. There were no significant interactions between curiosity and moral target type for either pattern learning or broadness of applicability.

**Individual Differences**

Individual difference traits were again z-scored prior to analyses. Correlations among individual difference are presented in Table S7.

| Measure | *1* | *2* | *3* | *4* | *5* |
| --- | --- | --- | --- | --- | --- |
| 1. Morbid Curiosity | -- |  |  |  |  |
| 2. Need for Cognition | .10 | -- |  |  |  |
| 3. Belief in a Just World | .001 | .04 | -- |  |  |
| 4. Imaginative Resistance | -.33*** | -.08 | .16*** | -- |  |
| 5. Perspective-taking Empathy | .13* | .26*** | .10 | .07 | -- |

**Table S7*.*** Correlation matrix for individual difference measures.

*Note: *p <* .05*, **p <* .01*, ***p <* .001. Holm method for p-value adjustment, *N* = 593-595.

Each model included the individual difference traits, phase one decision, and their interaction term. Each trait again predicted more moral curiosity, *p*’s < 0.001, except for imaginative resistance, which did not significantly predict curiosity. We again report the results for the imaginative resistance model. Unlike in Experiment 2a, curiosity for morally bad targets decreased as imaginative resistance increased, *b* = −0.15, *SE* = 0.06, *t*(542) = −2.75, *p* = .006, *r* = 0.12, *95% CI* [-0.14, -0.02] (see Figure 9). People who are more resistant to imagining moral deviance were less curious for information about the morally bad targets. The other individual differences and full statistics are reported in the Supplemental Materials.

**
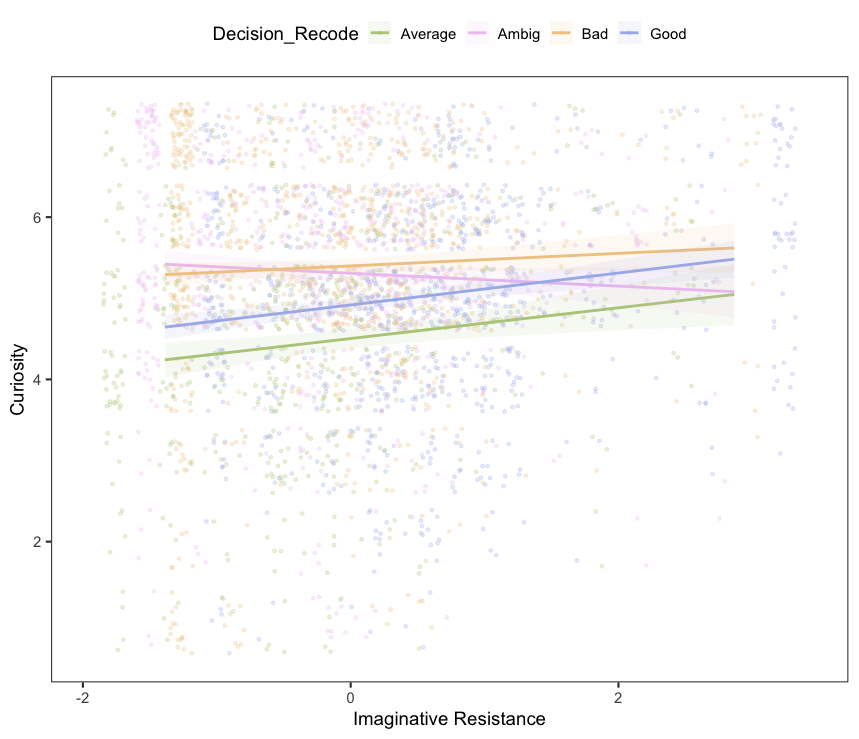
**
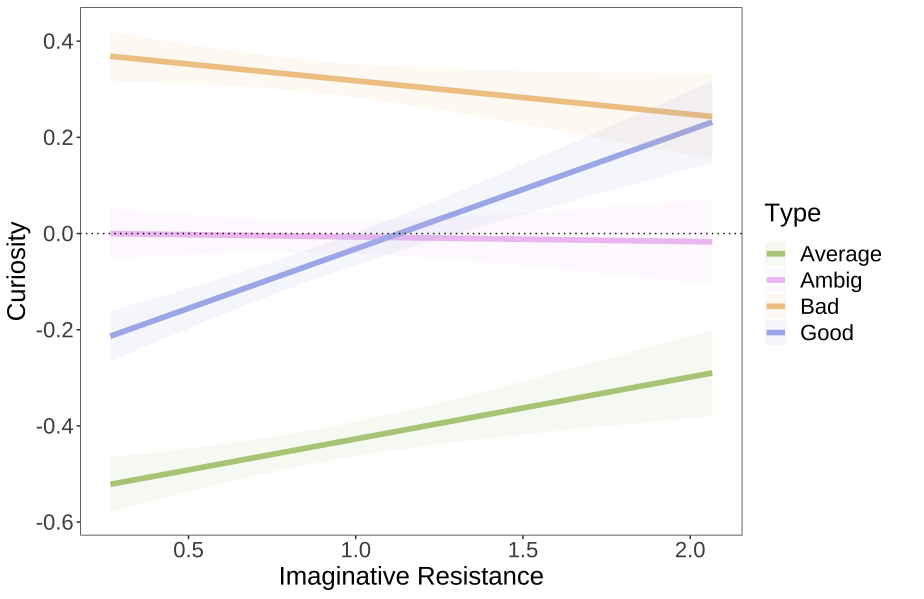


**Figure S11.** Interaction between imaginative resistance (scaled) and phase one moral target selection in Experiment 2b. Confidence intervals represent +/-1 SE. Model is fit with participants included as random intercepts.

**Additional individual differences**

We used the same analysis strategy from Experiment 2a. Random intercepts and slopes are included for all models.

***Belief in a Just World***

There was again a marginal main effect of Belief in a Just World on curiosity, *b* = 0.17, *SE* = 0.06, *t*(552) = 2.69, *p* = .007, *r* = 0.11. People high in just world beliefs were more curious about the moral minds of others. There were no significant interactions.

***Perspective taking***

Perspective taking empathy was also a significant predictor of moral curiosity, *b* = 0.13, *SE* = 0.04, *t*(547) = 3.59, *p* < .001, *r* = 0.15. There were no significant interactions.

***Morbid curiosity***

Morbid curiosity significantly predicted more curiosity, *b* = 0.13, *SE* = 0.04, *t*(1197) = 3.49, *p* < .001, *r* = 0.10 (see Figure S9). There was also a significant interaction between morbid curiosity and the morally bad target, *b* = 0.20, SE = 0.03, t(5479) = 6.42, p < .001, r = 0.09 and morally ambiguous, *b* = 0.08, *SE* = 0.03, *t*(5456) = 2.47, *p* = .013, *r* = 0.03, relative to morally average. Morally bad targets were most interesting to those high in morbid curiosity. The moral targets with morally bad ratings, even if only slightly, elicited more curiosity from those high in morbid curiosity.

**
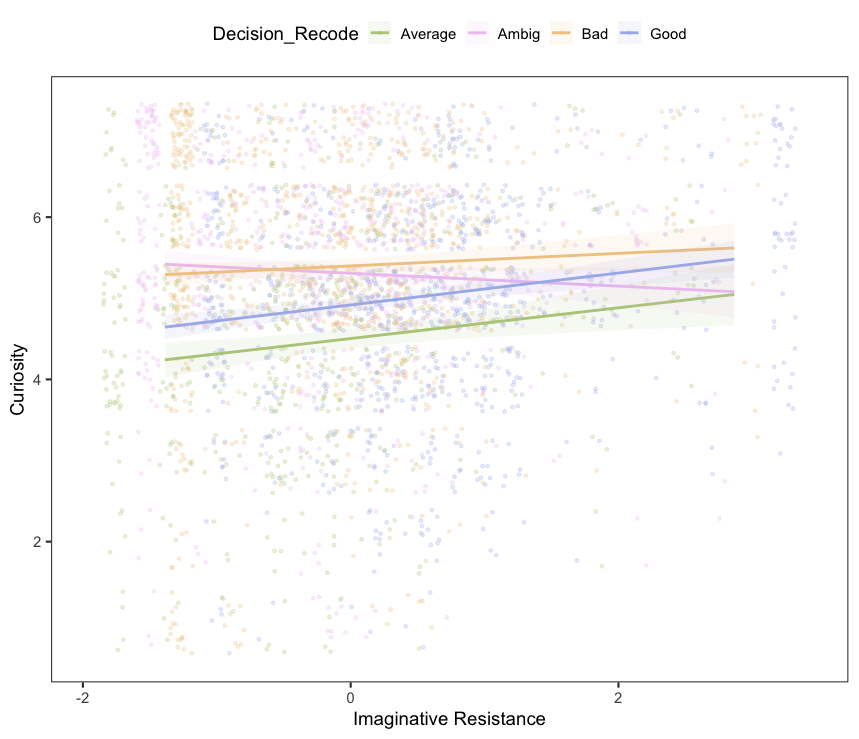
**


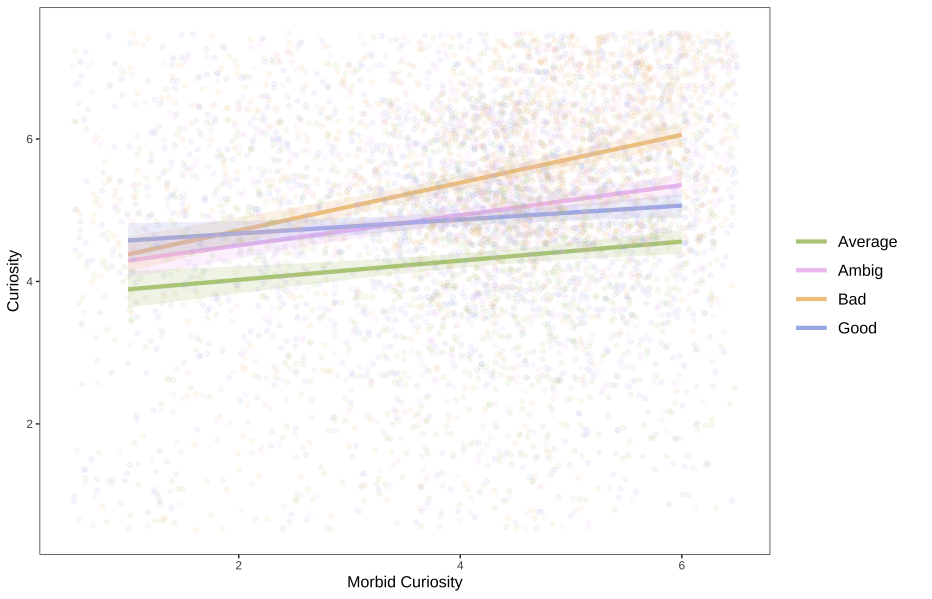


**Figure S12.** Individual differences in Morbid Curiosity predicting curiosity for each of the phase one moral agent type decisions in Experiment 2b. Shading represents 95% Confidence Intervals.

**Need for Cognition**. NFC was now associated with increases in curiosity. However, there were significant interactions such that higher trait NFC was associated with more moral curiosity for good moral targets, b = 0.12, SE = 0.04, t(5429) = 2.98, p = .003, r = 0.04 and bad moral targets, b = 0.13, SE = 0.04, t(5451) = 3.07, p = .002, r = 0.04. This pattern was unexpected given the findings of Experiment 2a.

## **Experiment 3**

**Method**

***Self-reported Interest***

We again measured self-reported interest for each person. We asked participants “How interesting is this person?” on a trial level, again rated on a scale from 1 = *Not at all* to 9 = *Extremely*.

**Individual Differences**

**Morbid Curiosity.** The same six items were selected from the Morbid Curiosity Scale (Scrivner, 2021).

**Need for Cognition.** For this experiment, we used the Need for Cognition scale (NFC; Cacioppo & Petty, 1982; 1984; Cacciopio et al., 2013).

**Single item Need for Consistency.** We also included the single item Need for Consistency item (Nichols & Webster, 2014). The item is “I make an effort to appear consistent to others,” rated from 1 = *strongly disagree* to 9 = *strongly agree*. This is reported in the Supplemental Material.

**Evil Essentialism**. Essentialism of good and evil was measured by adapting a measure of gender essentialism (Skewes et al., 2018) to refer to “good” and “evil” people rather than “men” and “women”. A representative sample item is “Differences between good and evil people are primarily determined by biology”, rated from 1 = *strongly disagree* to 7 = *strongly agree*. This measure also had high internal reliability and was collapsed into a single variable (α = 0.76). This is reported in the Supplemental Material.

**Results**

**Self-reported interest**

When we entered self-reported interest as the outcome variable, the results mirrored that of the pilot experiment. Participants report that morally good individuals are more interesting than ambiguous, *b* = 0.63, *SE* = 0.08, *t*(238) = 8.12, *p* < .001, *r* = 0.47, *95% CI* [0.48, 0.78] and bad people, *b* = 1.32, *SE* = 0.10, *t*(238) = 13.47, *p* < .001, *r* = 0.66, *95% CI* [1.13, 1.52]. Self-reported interest was highest for morally good targets.

**Individual Differences**

For this experiment, we included essentialism and need for consistency as new traits. Need for Cognition and Morbid Curiosity were both measured in each of the previous experiments. For each individual difference trait, we ‘glmer’ specified a separate model that included the trait and the Moral Agent Type as predictors as well as their interaction term to predict “Learn” deck choice. Correlations among individual differences are reported in Table S8 below.

| Measure | *1* | *2* | *3* | *4* |
| --- | --- | --- | --- | --- |
| 1. Morbid Curiosity | -- |  |  |  |
| 2. Evil Essentialism | .04 | -- |  |  |
| 3. Need for Cognition | .09 | -.24** | -- |  |
| 4. Need for Consistency | .04 | .17* | -.05 | -- |

**Table S8.** Correlations among individual difference traits for Experiment 3.

*Note: *p <* .05*, **p <* .01*, ***p <* .001

For Morbid Curiosity, there was no significant interaction between the trait and Moral Agent Type. The marginal main effect of Morbid Curiosity was itself a significant predictor of “Learn” Deck choice suggesting that curiosity, even of the morbid variety, indeed predicts more engagement with the minds of others, *b* = 0.24, *SE* = 0.09, *z* = 2.64, *p* = .01, *r* = 0.07, *95% CI* [0.08, 0.48]. There were no statistically significant effects for the Need for Cognition, Evil essentialism, or Need for Consistency.

**Discussion**

We found that self-reported interest showed the opposite pattern of results and may instead reflect a more general value judgment rather than cognitive engagement (i.e., the word “interest” may have a positive connotation that “curiosity” does not). Participants self-reported being most interested in morally good others. Morality is central to our self-concept and self-image (Strohminger & Nichols, 2014; Strohminger et al., 2017), and is therefore subject to self-presentation biases. Indeed, research suggests that participants are less likely to prefer movies with bad characters when self-presentation concerns are salient (Krause & Rucker, 2020). As such, the self-reported interest judgment may reflect either self-presentation concerns or it may have reflected interest in the kind of information available in the “Describe” deck.

## **Experiment 4**

**Results**

**Phase One: Curiosity and interest**

Contrary to our predictions, there was no significant difference between phase one ambiguity decision on ratings of curiosity, *b* = 0.05, *SE* = 0.05, *t*(347) = 0.98, *p* = .327, *r* = 0.05, *95% CI* [-0.04, 0.14]. This was also true for interest; no statistically significant difference between phase one decisions emerged for interest, *b* = 0.70, *SE* = 0.05, *t*(351) = 0.70, *r* = 0.04, *p* = .483, *95% CI* [-0.06, 0.13].

***Phase Three: Revealed information judgments***

**Normality**. Next, we tested whether phase one decision predicted how ideal and average people rated the revealed information. We found that morally ambiguous targets were rated as less ideal, *b* = −0.45, *SE* = 0.05, *t*(377) = −9.24, *p* < .001, *r* = 0.43, *95% CI* [-0.54, -0.35], but more average, *b* = −0.57, *SE* = 0.06, *t*(377) = −9.87, *p* < .001, *r* = 0.45, *95% CI* [-0.69, -0.45] than aesthetically ambiguous targets (see Figure S13). Aesthetically ambiguous information was rated as more normal than morally ambiguous information.


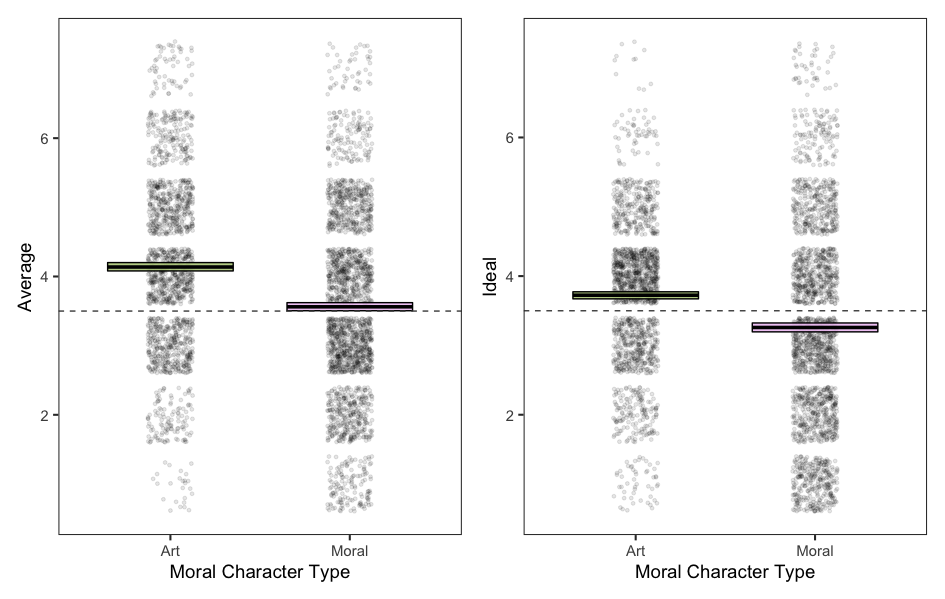


**Figure S13.** Patterns of results for the average and ideal ratings in Experiment 3. The thick black line in the boxes represents the mean, and the edges represent mean standard error from bootstrap.

**Satisfaction**. We then tested whether feelings of satisfaction differed based on phase one decision. Results showed that morally ambiguous targets were more satisfying to learn about than aesthetically ambiguous ones, *b* = 0.33, *SE* = 0.06, *t*(366) = 5.61, *p* < .001, *r* = 0.*28*, *95% CI* [0.22, 0.45]. We also explored whether curiosity and phase one decision together predicted satisfaction. We entered curiosity, decision type, and their interaction term to predict satisfaction. The results yielded a statistically significant interaction, *b* = −0.07, *SE* = 0.03, *t*(1251) = −2.51, *p* = .012, *r* = 0.07, *95% CI* [-0.12, -0.01], such that the more curiosity participants reported, the more satisfaction they felt. This pattern was particularly pronounced in the moral domain.

**Perceived Learning**. The correlation between the utility and broadness of applicability item were only slightly positively correlated (*r* = .18, *p* < 0.001) and the reliability did not meet the preregistered cutoff (Cronbach’s alpha = .64). The three items were investigated separately. Participants reported greater utility of learning for the moral compared to the aesthetic phase one target, *b* = 0.64, *SE* = 0.05, *t*(353) = 12.54, *p* < .001, *r* = 0.55, *95% CI* [0.54, 0.74]. Similarly, moral ambiguity predicted significantly higher judgments that the information revealed a genuine pattern, *b* = 0.69, *SE* = 0.06, *t*(371) = 11.27, *p* < .001, *r* = 0.51, *95% CI* [0.57, 0.82], and that it applies broadly rather than narrowly, *b* = 0.29, *SE* = 0.07, *t*(381) = 4.03, *p* < .001, *r* = 0.20, *95% CI* [0.14, 0.43].

# **Model Comparisons**

Here we report model comparisons for each experiment and for each key dependent variable. For all models shown below, when additional predictors are included in models, predictors were entered simultaneously. The table headings specify the random-effects structures for each model.

## **Experiment 2a**

**Phase 2: Curiosity**

**Table S9.** *Model comparisons for predicting the outcome curiosity. Models 1-4 specify random intercepts for subjects (1|Subject). Model 5 includes by-participant random slopes and intercepts.*

**
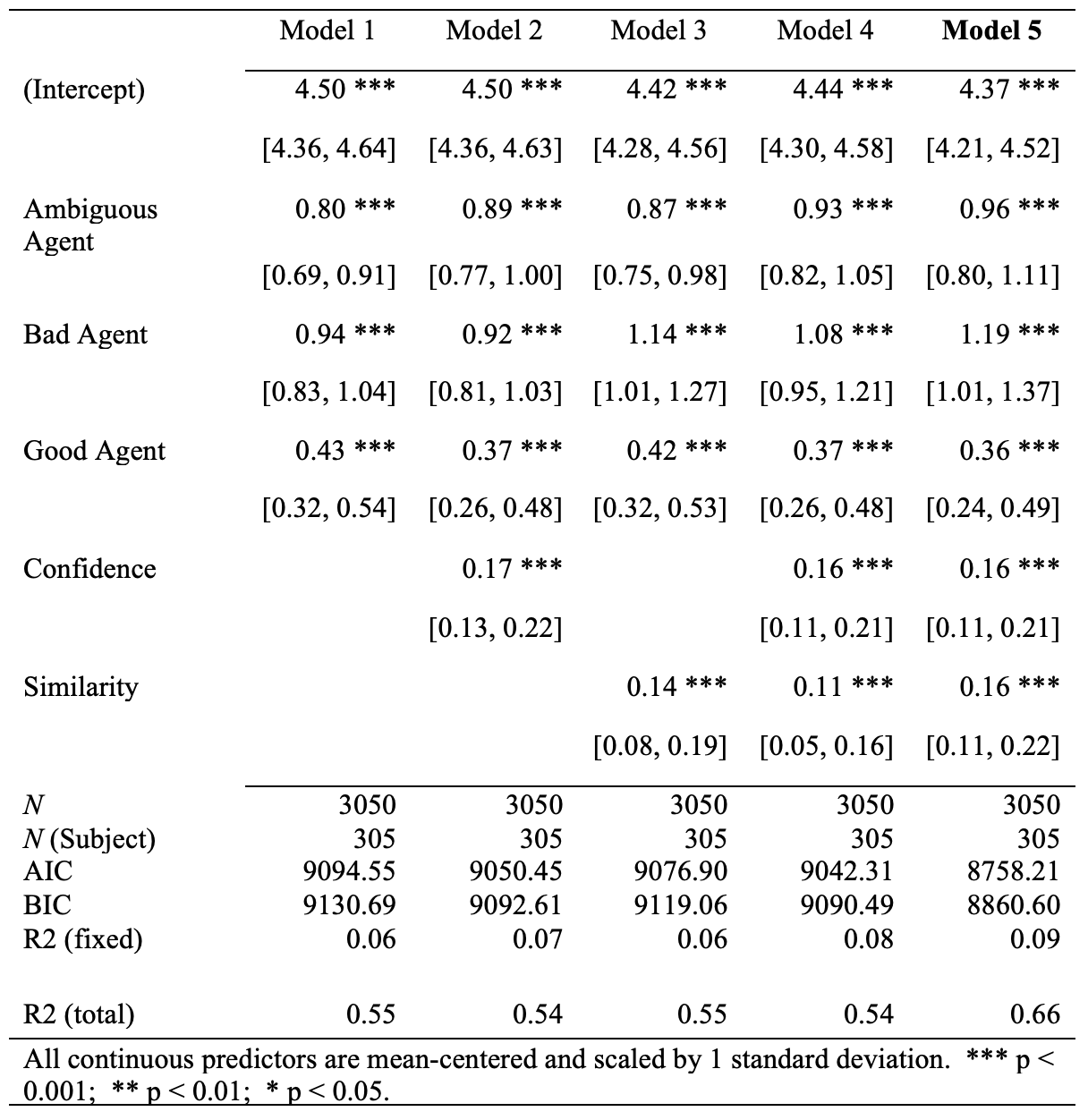
**

**Phase 2: Confidence**

**Table S10.** *Model comparisons for predicting the outcome confidence. Each model includes predictors simultaneously. Models 1-4 include a random intercept for subjects (1|Subject). Model 5 includes by-participant random slopes and intercepts.*

|  | Model 1 | Model 2 | Model 3 | Model 4 | Model 5 |  |
| --- | --- | --- | --- | --- | --- | --- |
| (Intercept) | 4.13 *** | 4.23 *** | 3.98 *** | 4.07 *** | 4.03 *** |  |
|  | [3.99, 4.28] | [4.09, 4.37] | [3.84, 4.12] | [3.93, 4.21] | [3.90, 4.16] |  |
| Ambiguous Agent | -0.75 *** | -0.88 *** | -0.61 *** | -0.74 *** | -0.70 *** |  |
|  | [-0.88, -0.62] | [-1.01, -0.75] | [-0.74, -0.48] | [-0.87, -0.60] | [-0.85, -0.55] |  |
| Bad Agent | 0.14 * | -0.00 | 0.58 *** | 0.42 *** | 0.43 *** |  |
|  | [0.02, 0.27] | [-0.13, 0.12] | [0.43, 0.73] | [0.26, 0.57] | [0.24, 0.63] |  |
| Good Agent | 0.51 *** | 0.44 *** | 0.50 *** | 0.44 *** | 0.46 *** |  |
|  | [0.39, 0.64] | [0.32, 0.57] | [0.38, 0.62] | [0.32, 0.56] | [0.34, 0.59] |  |
| Curiosity |  | 0.22 *** |  | 0.20 *** | 0.18 *** |  |
|  |  | [0.17, 0.28] |  | [0.15, 0.25] | [0.13, 0.24] |  |
| Similarity |  |  | 0.30 *** | 0.28 *** | 0.30 *** |  |
|  |  |  | [0.23, 0.36] | [0.22, 0.34] | [0.24, 0.36] |  |
| *N* | 3050 | 3050 | 3050 | 3050 | 3050 |  |
| *N* (Subject) | 305 | 305 | 305 | 305 | 305 |  |
| AIC | 9781.01 | 9728.47 | 9698.55 | 9655.24 | 9394.71 |  |
| BIC | 9817.15 | 9770.63 | 9740.71 | 9703.42 | 9497.10 |  |
| R2 (fixed) | 0.10 | 0.12 | 0.13 | 0.15 | 0.15 |  |
| R2 (total) | 0.48 | 0.47 | 0.47 | 0.47 | 0.60 |  |
| All continuous predictors are mean-centered and scaled by 1 standard deviation. *** p < 0.001; ** p < 0.01; * p < 0.05. | | | | | | |

**Phase 2: Expected Learning about Human Nature**

**Table S11.** *Model comparisons for predicting the outcome expected learning. Models 1 and 2 include a random intercept for subjects (1|Subject). Models 3-4 include by-participant random slopes and intercepts.*

|  | Model 1 | Model 2 | Model 3 | Model 4 |
| --- | --- | --- | --- | --- |
| (Intercept) | 4.17 *** | 4.06 *** | 4.06 *** | 4.35 *** |
|  | [4.07, 4.27] | [3.96, 4.16] | [3.96, 4.16] | [4.26, 4.44] |
| Ambiguous Agent | 0.11 ** | 0.33 *** | 0.22 *** | 0.02 |
|  | [0.03, 0.20] | [0.23, 0.42] | [0.13, 0.30] | [-0.06, 0.11] |
| Bad Agent | 0.44 *** | 0.74 *** | 0.79 *** | 0.21 *** |
|  | [0.35, 0.52] | [0.61, 0.87] | [0.69, 0.88] | [0.09, 0.33] |
| Good Agent | 0.39 *** | 0.25 *** | 0.32 *** | 0.04 |
|  | [0.31, 0.47] | [0.14, 0.35] | [0.24, 0.40] | [-0.05, 0.14] |
| Confidence |  | 0.25 *** |  | 0.20 *** |
|  |  | [0.21, 0.29] |  | [0.17, 0.24] |
| Similarity |  | 0.24 *** | 0.26 *** | 0.18 *** |
|  |  | [0.20, 0.28] | [0.22, 0.30] | [0.14, 0.22] |
| Curiosity |  |  |  | 0.58 *** |
|  |  |  |  | [0.55, 0.62] |
| N | 6011 | 6011 | 6011 | 6011 |
| N (Subj) | 606 | 606 | 606 | 606 |
| AIC | 18957.68 | 18292.63 | 18812.05 | 17341.18 |
| BIC | 18997.89 | 18406.55 | 18858.96 | 17461.80 |
| R2 (fixed) | 0.02 | 0.07 | 0.03 | 0.27 |
| R2 (total) | 0.49 | 0.60 | 0.49 | 0.60 |
| All continuous predictors are mean-centered and scaled by 1 standard deviation. *** p < 0.001; ** p < 0.01; * p < 0.05. | | | | |

**Phase 3: Satisfaction**

**Table S12.** *Model comparisons for predicting the outcome satisfaction. Model 1 includes a random intercept for subjects (1|Subject). Models 2-3 include by-participant random slopes and intercepts.*

|  | Model 1 | Model 2 | Model 3 |  |
| --- | --- | --- | --- | --- |
| (Intercept) | 3.97 *** | 3.80 *** | 4.00 *** |  |
|  | [3.81, 4.12] | [3.65, 3.96] | [3.84, 4.15] |  |
| Ambiguous Agent | 0.20 * | 0.33 *** | 0.10 |  |
|  | [0.04, 0.36] | [0.16, 0.50] | [-0.07, 0.28] |  |
| Bad Agent | -0.64 *** | -0.31 ** | -0.60 *** |  |
|  | [-0.79, -0.49] | [-0.53, -0.10] | [-0.82, -0.38] |  |
| Good Agent | 1.00 *** | 0.92 *** | 0.81 *** |  |
|  | [0.84, 1.15] | [0.74, 1.11] | [0.63, 1.00] |  |
| Similarity |  | 0.22 *** | 0.19 *** |  |
|  |  | [0.14, 0.30] | [0.11, 0.27] |  |
| Curiosity |  |  | 0.38 *** |  |
|  |  |  | [0.26, 0.50] |  |
| Ambiguity*Curious |  |  | -0.12 |  |
|  |  |  | [-0.28, 0.04] |  |
| Bad*Curious |  |  | -0.05 |  |
|  |  |  | [-0.21, 0.11] |  |
| Good*Curious |  |  | 0.06 |  |
|  |  |  | [-0.10, 0.22] |  |
| N | 3050 | 3050 | 3050 |  |
| N (Subject) | 305 | 305 | 305 |  |
| AIC | 11066.08 | 10788.85 | 10703.36 |  |
| BIC | 11102.22 | 10885.21 | 10823.82 |  |
| R2 (fixed) | 0.13 | 0.14 | 0.19 |  |
| R2 (total) | 0.38 | 0.51 | 0.49 |  |
| All continuous predictors are mean-centered and scaled by 1 standard deviation. *** p < 0.001; ** p < 0.01; * p < 0.05. | | | | |

**Phase 3: Perceived utility of Learning**

**Table S13.** *Model comparisons for predicting the outcome perceived utility of learning. Model 1 includes a random intercept for subjects (1|Subject). Models 2-4 include by-participant random slopes and intercepts.*

|  | Model 1 | Model 2 | Model 3 | Model 4 |  |
| --- | --- | --- | --- | --- | --- |
| (Intercept) | 3.91 *** | 3.80 *** | 3.92 *** | 4.00 *** |  |
|  | [3.73, 4.08] | [3.63, 3.98] | [3.75, 4.10] | [3.83, 4.17] |  |
| Ambiguous Agent | 0.20 ** | 0.27 ** | 0.12 | 0.05 |  |
|  | [0.07, 0.34] | [0.11, 0.43] | [-0.05, 0.29] | [-0.12, 0.21] |  |
| Bad Agent | -0.08 | 0.16 | -0.05 | -0.24 ** |  |
|  | [-0.21, 0.05] | [-0.02, 0.35] | [-0.24, 0.14] | [-0.40, -0.08] |  |
| Good Agent | 0.43 *** | 0.41 *** | 0.35 *** | 0.34 *** |  |
|  | [0.30, 0.56] | [0.26, 0.56] | [0.19, 0.50] | [0.19, 0.50] |  |
| Similarity |  | 0.15 *** | 0.13 *** |  |  |
|  |  | [0.09, 0.22] | [0.06, 0.20] |  |  |
| Curiosity |  |  | 0.24 *** | 0.26 *** |  |
|  |  |  | [0.13, 0.35] | [0.14, 0.37] |  |
| Ambiguous*Curiosity |  |  | 0.04 | 0.03 |  |
|  |  |  | [-0.12, 0.19] | [-0.12, 0.18] |  |
| Bad*Curiosity |  |  | 0.03 | 0.01 |  |
|  |  |  | [-0.11, 0.17] | [-0.13, 0.15] |  |
| Good*Curiosity |  |  | 0.05 | 0.05 |  |
|  |  |  | [-0.09, 0.19] | [-0.09, 0.19] |  |
| N | 3050 | 3050 | 3050 | 3050 |  |
| N (Subj) | 305 | 305 | 305 | 305 |  |
| AIC | 10276.54 | 10168.84 | 10121.09 | 10127.53 |  |
| BIC | 10312.68 | 10265.21 | 10241.55 | 10241.97 |  |
| R2 (fixed) | 0.02 | 0.02 | 0.05 | 0.04 |  |
| R2 (total) | 0.55 | 0.63 | 0.60 | 0.61 |  |
| All continuous predictors are mean-centered and scaled by 1 standard deviation. *** p < 0.001; ** p < 0.01; * p < 0.05. | | | | | |

**Phase 3: Perceived patterns**

**Table S14.** *Model comparisons for predicting the outcome perception of patterns. Model 1 includes a random intercept for subjects (1|Subject). Models 2-4 include by-participant random slopes and intercepts.*

|  | Model 1 | Model 2 | Model 3 | Model 4 |  |
| --- | --- | --- | --- | --- | --- |
| (Intercept) | 4.82 *** | 4.69 *** | 4.72 *** | 4.84 *** |  |
|  | [4.68, 4.96] | [4.55, 4.83] | [4.57, 4.87] | [4.69, 4.98] |  |
| Ambiguous Agent | -0.55 *** | -0.44 *** | -0.49 *** | -0.59 *** |  |
|  | [-0.69, -0.40] | [-0.60, -0.28] | [-0.66, -0.32] | [-0.76, -0.43] |  |
| Bad Agent | -0.90 *** | -0.56 *** | -0.63 *** | -0.93 *** |  |
|  | [-1.04, -0.76] | [-0.77, -0.36] | [-0.85, -0.41] | [-1.12, -0.74] |  |
| Good Agent | -0.33 *** | -0.36 *** | -0.35 *** | -0.35 *** |  |
|  | [-0.48, -0.19] | [-0.54, -0.18] | [-0.53, -0.16] | [-0.54, -0.16] |  |
| Self-identification |  | 0.21 *** | 0.19 *** |  |  |
|  |  | [0.14, 0.28] | [0.12, 0.26] |  |  |
| Curiosity |  |  | 0.05 | 0.07 |  |
|  |  |  | [-0.07, 0.16] | [-0.04, 0.19] |  |
| Ambiguous*Curiosity |  |  | 0.09 | 0.08 |  |
|  |  |  | [-0.06, 0.24] | [-0.07, 0.24] |  |
| Bad*Curiosity |  |  | 0.05 | 0.02 |  |
|  |  |  | [-0.11, 0.20] | [-0.13, 0.18] |  |
| Good*Curiosity |  |  | 0.17 * | 0.17 * |  |
|  |  |  | [0.01, 0.32] | [0.01, 0.33] |  |
| N | 3050 | 3050 | 3050 | 3050 |  |
| N (Subj) | 305 | 305 | 305 | 305 |  |
| AIC | 10495.79 | 10242.42 | 10245.31 | 10266.21 |  |
| BIC | 10531.92 | 10338.79 | 10365.77 | 10380.64 |  |
| R2 (fixed) | 0.04 | 0.05 | 0.06 | 0.05 |  |
| R2 (total) | 0.33 | 0.48 | 0.47 | 0.48 |  |
| All continuous predictors are mean-centered and scaled by 1 standard deviation. *** p < 0.001; ** p < 0.01; * p < 0.05. | | | | | |

**Phase 3: Perceived broadness**

**Table S15.** *Model comparisons for predicting the outcome perception of broadness. Model 1 includes a random intercept for subjects (1|Subject). Models 2-4 include by-participant random slopes and intercepts.*

|  | Model 1 | Model 2 | Model 3 | Model 4 |
| --- | --- | --- | --- | --- |
| (Intercept) | 5.95 *** | 5.87 *** | 5.82 *** | 5.92 *** |
|  | [5.81, 6.10] | [5.71, 6.03] | [5.65, 5.99] | [5.75, 6.08] |
| Ambiguous Agent | -1.17 *** | -1.10 *** | -1.05 *** | -1.14 *** |
|  | [-1.33, -1.01] | [-1.28, -0.92] | [-1.25, -0.86] | [-1.33, -0.95] |
| Bad Agent | -1.72 *** | -1.47 *** | -1.45 *** | -1.70 *** |
|  | [-1.87, -1.57] | [-1.69, -1.24] | [-1.69, -1.21] | [-1.91, -1.49] |
| Good Agent | -1.14 *** | -1.17 *** | -1.11 *** | -1.11 *** |
|  | [-1.29, -0.98] | [-1.36, -0.98] | [-1.30, -0.91] | [-1.31, -0.92] |
| Similarity |  | 0.16 *** | 0.16 *** |  |
|  |  | [0.09, 0.24] | [0.08, 0.24] |  |
| Curiosity |  |  | -0.12 | -0.09 |
|  |  |  | [-0.25, 0.01] | [-0.23, 0.04] |
| Ambiguous*Curiosity |  |  | 0.19 * | 0.18 * |
|  |  |  | [0.02, 0.36] | [0.01, 0.35] |
| Bad*Curiosity |  |  | 0.27 ** | 0.25 ** |
|  |  |  | [0.10, 0.44] | [0.07, 0.42] |
| Good*Curiosity |  |  | 0.21 * | 0.21 * |
|  |  |  | [0.04, 0.38] | [0.04, 0.39] |
| N | 3050 | 3050 | 3050 | 3050 |
| N (Subject) | 305 | 305 | 305 | 305 |
| AIC | 10952.69 | 10795.14 | 10805.56 | 10815.02 |
| BIC | 10988.83 | 10891.50 | 10926.02 | 10929.45 |
| R2 (fixed) | 0.12 | 0.13 | 0.13 | 0.12 |
| R2 (total) | 0.30 | 0.44 | 0.44 | 0.44 |
| All continuous predictors are mean-centered and scaled by 1 standard deviation. *** p < 0.001; ** p < 0.01; * p < 0.05. | | | | |

## **Experiment 2b**

**Phase 2: Curiosity**

**Table S16.** *Model comparisons for Phase 1 and 2 predictors with the outcome curiosity. Each model includes predictors simultaneously. Models 1-4 specify a random intercept for subjects (1|Subject). Model 4 includes by-participant random slopes and intercepts.*

|  | Model 1 | Model 2 | Model 3 | Model 4 | Model 5 |
| --- | --- | --- | --- | --- | --- |
| (Intercept) | 4.32 *** | 4.33 *** | 4.25 *** | 4.27 *** | 4.22 *** |
|  | [4.22, 4.43] | [4.23, 4.43] | [4.15, 4.36] | [4.17, 4.37] | [4.11, 4.33] |
| Ambiguous Agent | 0.64 *** | 0.72 *** | 0.71 *** | 0.77 *** | 0.80 *** |
|  | [0.56, 0.73] | [0.63, 0.80] | [0.63, 0.80] | [0.68, 0.85] | [0.69, 0.91] |
| Bad Agent | 1.16 *** | 1.13 *** | 1.38 *** | 1.33 *** | 1.37 *** |
|  | [1.08, 1.24] | [1.05, 1.22] | [1.28, 1.48] | [1.23, 1.43] | [1.24, 1.50] |
| Good Agent | 0.58 *** | 0.53 *** | 0.54 *** | 0.50 *** | 0.50 *** |
|  | [0.50, 0.66] | [0.45, 0.61] | [0.46, 0.62] | [0.42, 0.58] | [0.41, 0.60] |
| Confidence |  | 0.17 *** |  | 0.15 *** | 0.17 *** |
|  |  | [0.13, 0.21] |  | [0.11, 0.19] | [0.13, 0.20] |
| Similarity |  |  | 0.16 *** | 0.14 *** | 0.16 *** |
|  |  |  | [0.12, 0.20] | [0.10, 0.18] | [0.11, 0.20] |
| N | 6011 | 6011 | 6011 | 6011 | 6011 |
| N (Subj) | 606 | 606 | 606 | 606 | 606 |
| AIC | 18765.97 | 18699.18 | 18708.96 | 18661.10 | 18162.39 |
| BIC | 18806.18 | 18746.09 | 18755.87 | 18714.71 | 18276.31 |
| R2 (fixed) | 0.07 | 0.08 | 0.08 | 0.09 | 0.10 |
| R2 (total) | 0.53 | 0.52 | 0.53 | 0.52 | 0.66 |
| All continuous predictors are mean-centered and scaled by 1 standard deviation. *** p < 0.001; ** p < 0.01; * p < 0.05. | | | | | |

**Phase 2: Confidence**

**Table S17.** *Model comparisons for Phase 1 and 2 predictors with the outcome confidence. Each model includes predictors simultaneously. Models 1-4 specify a random intercept for subjects (1|Subject). Model 4 includes by-participant random slopes and intercepts.*

|  | Model 1 | Model 2 | Model 3 | Model 4 | Model 5 |
| --- | --- | --- | --- | --- | --- |
| (Intercept) | 4.13 *** | 4.20 *** | 4.01 *** | 4.08 *** | 4.08 *** |
|  | [4.02, 4.23] | [4.10, 4.31] | [3.91, 4.12] | [3.98, 4.19] | [3.98, 4.18] |
| Ambiguous Agent | -0.68 *** | -0.75 *** | -0.56 *** | -0.63 *** | -0.62 *** |
|  | [-0.76, -0.59] | [-0.84, -0.66] | [-0.65, -0.48] | [-0.72, -0.54] | [-0.73, -0.52] |
| Bad Agent | 0.22 *** | 0.08 | 0.59 *** | 0.46 *** | 0.45 *** |
|  | [0.13, 0.30] | [-0.01, 0.17] | [0.49, 0.69] | [0.35, 0.56] | [0.32, 0.57] |
| Good Agent | 0.50 *** | 0.44 *** | 0.43 *** | 0.38 *** | 0.38 *** |
|  | [0.42, 0.59] | [0.35, 0.52] | [0.35, 0.51] | [0.29, 0.46] | [0.29, 0.47] |
| Curiosity |  | 0.17 *** |  | 0.15 *** | 0.16 *** |
|  |  | [0.13, 0.21] |  | [0.11, 0.19] | [0.12, 0.20] |
| Similarity |  |  | 0.27 *** | 0.26 *** | 0.27 *** |
|  |  |  | [0.23, 0.31] | [0.22, 0.30] | [0.22, 0.31] |
| N | 6011 | 6011 | 6011 | 6011 | 6011 |
| N (Subj) | 606 | 606 | 606 | 606 | 606 |
| AIC | 19164.59 | 19099.20 | 19000.25 | 18953.31 | 18458.93 |
| BIC | 19204.80 | 19146.10 | 19047.16 | 19006.92 | 18572.86 |
| R2 (fixed) | 0.08 | 0.10 | 0.10 | 0.11 | 0.12 |
| R2 (total) | 0.55 | 0.53 | 0.55 | 0.54 | 0.66 |
| All continuous predictors are mean-centered and scaled by 1 standard deviation. *** p < 0.001; ** p < 0.01; * p < 0.05. | | | | | |

**Phase 2: Expected Learning about Human Nature**

**Table S18.** *Model comparisons for the outcome expected learning. Each model includes predictors simultaneously. Models 1-2 include a random intercept for subjects (1|Subject). Model 3 and 4 include by-participant random slopes and intercepts.*

|  | Model 1 | Model 2 | Model 3 | Model 4 |
| --- | --- | --- | --- | --- |
| (Intercept) | 4.17 *** | 4.06 *** | 4.06 *** | 4.35 *** |
|  | [4.07, 4.27] | [3.96, 4.16] | [3.96, 4.16] | [4.26, 4.44] |
| Ambiguous Agent | 0.11 ** | 0.33 *** | 0.22 *** | 0.02 |
|  | [0.03, 0.20] | [0.23, 0.42] | [0.13, 0.30] | [-0.06, 0.11] |
| Bad Agent | 0.44 *** | 0.74 *** | 0.79 *** | 0.21 *** |
|  | [0.35, 0.52] | [0.61, 0.87] | [0.69, 0.88] | [0.09, 0.33] |
| Good Agent | 0.39 *** | 0.25 *** | 0.32 *** | 0.04 |
|  | [0.31, 0.47] | [0.14, 0.35] | [0.24, 0.40] | [-0.05, 0.14] |
| Confidence |  | 0.25 *** |  | 0.20 *** |
|  |  | [0.21, 0.29] |  | [0.17, 0.24] |
| Similarity |  | 0.24 *** | 0.26 *** | 0.18 *** |
|  |  | [0.20, 0.28] | [0.22, 0.30] | [0.14, 0.22] |
| Curiosity |  |  |  | 0.58 *** |
|  |  |  |  | [0.55, 0.62] |
| N | 6011 | 6011 | 6011 | 6011 |
| N (Subj) | 606 | 606 | 606 | 606 |
| AIC | 18957.68 | 18292.63 | 18812.05 | 17341.18 |
| BIC | 18997.89 | 18406.55 | 18858.96 | 17461.80 |
| R2 (fixed) | 0.02 | 0.07 | 0.03 | 0.27 |
| R2 (total) | 0.49 | 0.60 | 0.49 | 0.60 |
| All continuous predictors are mean-centered and scaled by 1 standard deviation. *** p < 0.001; ** p < 0.01; * p < 0.05. | | | | |

**Phase 3: Satisfaction**

**Table S19.** *Model comparisons for the outcome satisfaction. Each model includes predictors simultaneously. Models 1-3 include a random intercept for subjects (1|Subject). Models 3 and 4 include by-participant random slopes and intercepts.*

|  | Model 1 | Model 2 | Model 3 | Model 4 |
| --- | --- | --- | --- | --- |
| (Intercept) | 3.95 *** | 3.83 *** | 3.82 *** | 4.02 *** |
|  | [3.84, 4.06] | [3.72, 3.94] | [3.71, 3.93] | [3.91, 4.13] |
| Ambiguous Agent | 0.25 *** | 0.37 *** | 0.37 *** | 0.18 ** |
|  | [0.14, 0.36] | [0.26, 0.48] | [0.25, 0.49] | [0.05, 0.30] |
| Bad Agent | -0.43 *** | -0.05 | -0.15 | -0.48 *** |
|  | [-0.54, -0.33] | [-0.18, 0.08] | [-0.31, 0.00] | [-0.64, -0.33] |
| Good Agent | 1.09 *** | 1.02 *** | 1.03 *** | 0.89 *** |
|  | [0.99, 1.20] | [0.91, 1.12] | [0.91, 1.14] | [0.78, 1.01] |
| Similarity |  | 0.28 *** | 0.23 *** | 0.19 *** |
|  |  | [0.23, 0.33] | [0.18, 0.28] | [0.14, 0.24] |
| Curiosity |  |  |  | 0.38 *** |
|  |  |  |  | [0.29, 0.46] |
| Ambiguous*Curiosity |  |  |  | -0.05 |
|  |  |  |  | [-0.16, 0.05] |
| Bad*Curiosity |  |  |  | -0.07 |
|  |  |  |  | [-0.18, 0.05] |
| Good*Curiosity |  |  |  | 0.19 *** |
|  |  |  |  | [0.09, 0.30] |
| N | 6009 | 6009 | 6009 | 6009 |
| N (Subj) | 605 | 605 | 605 | 605 |
| AIC | 21739.25 | 21634.05 | 21075.28 | 20822.05 |
| BIC | 21779.46 | 21680.96 | 21182.49 | 20956.07 |
| R2 (fixed) | 0.11 | 0.13 | 0.13 | 0.19 |
| R2 (total) | 0.40 | 0.40 | 0.56 | 0.53 |
| All continuous predictors are mean-centered and scaled by 1 standard deviation. *** p < 0.001; ** p < 0.01; * p < 0.05. | | | | |

**Phase 3: Perceived utility of Learning**

**Table S20.** *Model comparisons for predicting the outcome perceived utility of learning. Each model includes predictors simultaneously. Models 1-2 include a random intercept for subjects (1|Subject). Model 3-5 include by-participant random slopes and intercepts.*

|  | Model 1 | Model 2 | Model 3 | Model 4 | Model 5 |
| --- | --- | --- | --- | --- | --- |
| (Intercept) | 3.88 *** | 3.79 *** | 3.79 *** | 4.06 *** | 3.99 *** |
|  | [3.76, 4.01] | [3.67, 3.92] | [3.67, 3.91] | [3.95, 4.18] | [3.88, 4.11] |
| Ambiguous Agent | 0.25 *** | 0.33 *** | 0.33 *** | 0.07 | 0.14 * |
|  | [0.15, 0.34] | [0.24, 0.43] | [0.23, 0.44] | [-0.04, 0.17] | [0.03, 0.25] |
| Bad Agent | 0.11 * | 0.39 *** | 0.35 *** | -0.17 ** | 0.04 |
|  | [0.01, 0.20] | [0.28, 0.51] | [0.22, 0.49] | [-0.29, -0.05] | [-0.10, 0.18] |
| Good Agent | 0.50 *** | 0.44 *** | 0.45 *** | 0.33 *** | 0.30 *** |
|  | [0.41, 0.59] | [0.35, 0.54] | [0.34, 0.55] | [0.22, 0.43] | [0.19, 0.40] |
| Similarity |  | 0.21 *** | 0.18 *** |  | 0.15 *** |
|  |  | [0.17, 0.26] | [0.14, 0.23] |  | [0.10, 0.20] |
| Curiosity |  |  |  | 0.41 *** | 0.39 *** |
|  |  |  |  | [0.33, 0.48] | [0.32, 0.46] |
| Ambiguous*Curiosity |  |  |  | -0.14 ** | -0.14 ** |
|  |  |  |  | [-0.23, -0.04] | [-0.23, -0.04] |
| Bad* Curiosity |  |  |  | -0.16 ** | -0.13 * |
|  |  |  |  | [-0.26, -0.05] | [-0.24, -0.03] |
| Good* Curiosity |  |  |  | -0.01 | -0.01 |
|  |  |  |  | [-0.10, 0.09] | [-0.11, 0.08] |
| N | 6009 | 6009 | 6009 | 6009 | 6009 |
| N (Subj) | 605 | 605 | 605 | 605 | 605 |
| AIC | 20504.54 | 20430.14 | 20111.78 | 19958.28 | 19929.27 |
| BIC | 20544.75 | 20477.05 | 20219.00 | 20085.60 | 20063.29 |
| R2 (fixed) | 0.01 | 0.02 | 0.02 | 0.05 | 0.05 |
| R2 (total) | 0.56 | 0.56 | 0.65 | 0.63 | 0.62 |
| All continuous predictors are mean-centered and scaled by 1 standard deviation. *** p < 0.001; ** p < 0.01; * p < 0.05. | | | | | |

**Phase 3: Perceived patterns**

**Table S21.** *Model comparisons for predicting the outcome perception of patterns. Each model includes predictors simultaneously. Models 1-2 include a random intercept for subjects (1|Subject). Models 3-5 include by-participant random slopes and intercepts.*

|  | Model 1 | Model 2 | Model 3 | Model 4 | Model 5 |
| --- | --- | --- | --- | --- | --- |
| (Intercept) | 4.79 *** | 4.67 *** | 4.69 *** | 4.90 *** | 4.80 *** |
|  | [4.68, 4.89] | [4.56, 4.77] | [4.58, 4.79] | [4.79, 5.01] | [4.69, 4.91] |
| Ambiguous Agent | -0.50 *** | -0.38 *** | -0.40 *** | -0.61 *** | -0.51 *** |
|  | [-0.60, -0.39] | [-0.48, -0.28] | [-0.51, -0.28] | [-0.73, -0.49] | [-0.63, -0.39] |
| Bad Agent | -0.82 *** | -0.44 *** | -0.52 *** | -1.02 *** | -0.72 *** |
|  | [-0.92, -0.72] | [-0.56, -0.32] | [-0.67, -0.37] | [-1.16, -0.88] | [-0.88, -0.56] |
| Good Agent | -0.39 *** | -0.46 *** | -0.47 *** | -0.51 *** | -0.55 *** |
|  | [-0.49, -0.29] | [-0.56, -0.36] | [-0.60, -0.35] | [-0.64, -0.38] | [-0.68, -0.42] |
| Similarity |  | 0.28 *** | 0.24 *** |  | 0.21 *** |
|  |  | [0.23, 0.33] | [0.19, 0.29] |  | [0.16, 0.26] |
| Curiosity |  |  |  | 0.25 *** | 0.23 *** |
|  |  |  |  | [0.17, 0.34] | [0.14, 0.31] |
| Ambiguous*Curiosity |  |  |  | -0.06 | -0.07 |
|  |  |  |  | [-0.17, 0.04] | [-0.17, 0.04] |
| Bad* Curiosity |  |  |  | -0.05 | -0.02 |
|  |  |  |  | [-0.16, 0.07] | [-0.13, 0.10] |
| Good* Curiosity |  |  |  | 0.02 | 0.00 |
|  |  |  |  | [-0.10, 0.13] | [-0.11, 0.12] |
| N | 6009 | 6009 | 6009 | 6009 | 6009 |
| N (Subj) | 605 | 605 | 605 | 605 | 605 |
| AIC | 21154.99 | 21038.72 | 20664.05 | 20665.22 | 20610.16 |
| BIC | 21195.20 | 21085.63 | 20771.27 | 20792.54 | 20744.18 |
| R2 (fixed) | 0.03 | 0.05 | 0.05 | 0.05 | 0.07 |
| R2 (total) | 0.34 | 0.35 | 0.49 | 0.48 | 0.48 |
| All continuous predictors are mean-centered and scaled by 1 standard deviation. *** p < 0.001; ** p < 0.01; * p < 0.05. | | | | | |

**Phase 3: Perceived broadness**

**Table S22.** *Model comparisons for predicting the outcome perception of broadness. Each model includes predictors simultaneously. Models 1-2 include a random intercept for subjects (1|Subject). Models 3-5 include by-participant random slopes and intercepts.*

|  | Model 1 | Model 2 | Model 3 | Model 4 | Model 5 |
| --- | --- | --- | --- | --- | --- |
| (Intercept) | 4.79 *** | 4.67 *** | 4.69 *** | 4.90 *** | 4.80 *** |
|  | [4.68, 4.89] | [4.56, 4.77] | [4.58, 4.79] | [4.79, 5.01] | [4.69, 4.91] |
| Ambiguous Agent | -0.50 *** | -0.38 *** | -0.40 *** | -0.61 *** | -0.51 *** |
|  | [-0.60, -0.39] | [-0.48, -0.28] | [-0.51, -0.28] | [-0.73, -0.49] | [-0.63, -0.39] |
| Bad Agent | -0.82 *** | -0.44 *** | -0.52 *** | -1.02 *** | -0.72 *** |
|  | [-0.92, -0.72] | [-0.56, -0.32] | [-0.67, -0.37] | [-1.16, -0.88] | [-0.88, -0.56] |
| Good Agent | -0.39 *** | -0.46 *** | -0.47 *** | -0.51 *** | -0.55 *** |
|  | [-0.49, -0.29] | [-0.56, -0.36] | [-0.60, -0.35] | [-0.64, -0.38] | [-0.68, -0.42] |
| Similarity |  | 0.28 *** | 0.24 *** |  | 0.21 *** |
|  |  | [0.23, 0.33] | [0.19, 0.29] |  | [0.16, 0.26] |
| Curiosity |  |  |  | 0.25 *** | 0.23 *** |
|  |  |  |  | [0.17, 0.34] | [0.14, 0.31] |
| Ambiguous*Curiosity |  |  |  | -0.06 | -0.07 |
|  |  |  |  | [-0.17, 0.04] | [-0.17, 0.04] |
| Bad* Curiosity |  |  |  | -0.05 | -0.02 |
|  |  |  |  | [-0.16, 0.07] | [-0.13, 0.10] |
| Good* Curiosity |  |  |  | 0.02 | 0.00 |
|  |  |  |  | [-0.10, 0.13] | [-0.11, 0.12] |
| N | 6009 | 6009 | 6009 | 6009 | 6009 |
| N (Subj) | 605 | 605 | 605 | 605 | 605 |
| AIC | 21154.99 | 21038.72 | 20664.05 | 20665.22 | 20610.16 |
| BIC | 21195.20 | 21085.63 | 20771.27 | 20792.54 | 20744.18 |
| R2 (fixed) | 0.03 | 0.05 | 0.05 | 0.05 | 0.07 |
| R2 (total) | 0.34 | 0.35 | 0.49 | 0.48 | 0.48 |
| All continuous predictors are mean-centered and scaled by 1 standard deviation. *** p < 0.001; ** p < 0.01; * p < 0.05. | | | | | |

## **Experiment 3**

**Table S23.** *Model comparisons for predicting Deck decision. All models use good moral agents as the reference group and "bobyqa" as the optimizer. Models 1 include a random intercept for subjects (1|Subject). Model 2 specifies by-participant random slopes and intercepts. Model 3 includes predictors (moral agent type, similarity) simultaneously and includes by-participant random slopes and intercepts.*

|  | Model 1 | Model 2 | Model 3 |
| --- | --- | --- | --- |
| (Intercept) | -0.02 | -0.02 | -0.29 *** |
|  | [-0.17, 0.14] | [-0.18, 0.15] | [-0.45, -0.13] |
| Ambiguous Agent | -0.07 | -0.06 | 0.27 *** |
|  | [-0.19, 0.06] | [-0.21, 0.08] | [0.11, 0.42] |
| Bad Agent | 0.41 *** | 0.42 *** | 0.93 *** |
|  | [0.29, 0.54] | [0.28, 0.57] | [0.75, 1.10] |
| Similarity |  |  | 0.46 *** |
|  |  |  | [0.39, 0.53] |
| *N* | 7170 | 7170 | 7165 |
| *N* (Subj) | 239 | 239 | 239 |
| AIC | 9075.47 | 9060.38 | 8897.19 |
| BIC | 9102.98 | 9122.28 | 8965.96 |
| R2 (fixed) | 0.01 | 0.01 | 0.04 |
| R2 (total) | 0.25 | 0.27 | 0.33 |
| All continuous predictors are mean-centered and scaled by 1 standard deviation. *** p < 0.001; ** p < 0.01; * p < 0.05. | | | |

## **Experiment 4**

**Phase 2: Curiosity**

**Table S24.** *Model comparisons for predicting the outcome curiosity. Models 1 includes a random intercept for subjects (1|Subject). Model 2 includes by-participant random slopes and intercepts.*

|  | Model 1 | Model 2 | |
| --- | --- | --- | --- |
| (Intercept) | 6.04 *** | 6.03 *** | |
|  | [5.88, 6.20] | [5.87, 6.19] | |
| Moral Ambiguity | 0.03 | 0.03 | |
|  | [-0.05, 0.11] | [-0.06, 0.12] | |
| N | 3910 | 3910 | |
| N (Subj) | 391 | 391 | |
| AIC | 12986.29 | 12958.70 | |
| BIC | 13011.37 | 12996.33 | |
| R2 (fixed) | 0.00 | 0.00 | |
| R2 (total) | 0.67 | 0.69 | |
| All continuous predictors are mean-centered and scaled by 1 standard deviation. *** p < 0.001; ** p < 0.01; * p < 0.05. | | |  |

**Phase 3: Satisfaction**

**Table S25.** *Model comparisons for predicting the outcome satisfaction. Model 1 includes a random intercept for subjects (1|Subject). Models 2-3 include by-participant random slopes and intercepts.*

|  | | Model 1 | Model 2 | Model 3 |
| --- | --- | --- | --- | --- |
| (Intercept) | | 3.74 *** | 3.71 *** | 3.73 *** |
|  | | [3.63, 3.85] | [3.60, 3.82] | [3.63, 3.82] |
| Moral Ambiguity | | 0.30 *** | 0.31 *** | 0.29 *** |
|  | | [0.21, 0.39] | [0.19, 0.43] | [0.18, 0.41] |
| Curiosity | |  |  | 0.36 *** |
|  | |  |  | [0.29, 0.44] |
| Moral Ambiguity*Curiosity | |  |  | -0.12 * |
|  | |  |  | [-0.22, -0.02] |
| N | | 3910 | 3910 | 3910 |
| N (Subject) | | 391 | 391 | 391 |
| AIC | | 13509.15 | 13399.04 | 13320.20 |
| BIC | | 13534.24 | 13436.67 | 13370.37 |
| R2 (fixed) | | 0.01 | 0.01 | 0.05 |
| R2 (total) | | 0.34 | 0.41 | 0.37 |
| All continuous predictors are mean-centered and scaled by 1 standard deviation. *** p < 0.001; ** p < 0.01; * p < 0.05. | | | | |

**Phase 3: Perceived utility of Learning**

**Table S26.** *Model comparisons for predicting the outcome expected learning. Model 1 includes a random intercept for subjects (1|Subject). Model 2-3 includes by-participant random slopes and intercepts.*

|  | Model 1 | Model 2 | Model 3 | |
| --- | --- | --- | --- | --- |
| (Intercept) | 3.48 *** | 3.45 *** | 3.46 *** | |
|  | [3.34, 3.62] | [3.31, 3.59] | [3.33, 3.59] | |
| Moral Ambiguity | 0.63 *** | 0.64 *** | 0.63 *** | |
|  | [0.55, 0.70] | [0.54, 0.74] | [0.54, 0.73] | |
| Curiosity |  |  | 0.30 *** | |
|  |  |  | [0.23, 0.37] | |
| Moral Ambiguity* Curiosity |  |  | -0.01 | |
|  |  |  | [-0.09, 0.08] | |
| N | 3910 | 3910 | 3910 | |
| N (Subject) | 391 | 391 | 391 | |
| AIC | 12520.95 | 12396.99 | 12304.40 | |
| BIC | 12546.04 | 12434.62 | 12354.57 | |
| R2 (fixed) | 0.03 | 0.03 | 0.07 | |
| R2 (total) | 0.64 | 0.68 | 0.65 | |
| All continuous predictors are mean-centered and scaled by 1 standard deviation. *** p < 0.001; ** p < 0.01; * p < 0.05. | | | | |

**Phase 3: Perceived patterns**

**Table S27.** *Model comparisons for predicting the outcome perception of patterns. Model 1 includes a random intercept for subjects (1|Subject). Model 2-3 includes by-participant random slopes and intercepts.*

|  | Model 1 | Model 2 | Model 3 |
| --- | --- | --- | --- |
| (Intercept) | 3.56 *** | 3.52 *** | 3.52 *** |
|  | [3.45, 3.67] | [3.40, 3.63] | [3.40, 3.63] |
| Moral Ambiguity | 0.67 *** | 0.70 *** | 0.69 *** |
|  | [0.59, 0.75] | [0.58, 0.82] | [0.57, 0.81] |
| Curiosity |  |  | 0.12 ** |
|  |  |  | [0.04, 0.20] |
| Moral Ambiguity*Curiosity |  |  | 0.03 |
|  |  |  | [-0.08, 0.13] |
| N | 3910 | 3910 | 3910 |
| N (Subject) | 391 | 391 | 391 |
| AIC | 13400.70 | 13246.81 | 13241.03 |
| BIC | 13425.79 | 13284.44 | 13291.20 |
| R2 (fixed) | 0.05 | 0.05 | 0.06 |
| R2 (total) | 0.38 | 0.46 | 0.44 |
| All continuous predictors are mean-centered and scaled by 1 standard deviation. *** p < 0.001; ** p < 0.01; * p < 0.05. | | | |

**Phase 3: Perceived broadness**

**Table S28.** *Model comparisons for predicting the outcome perception of broadness. Model 1 includes a random intercept for subjects (1|Subject). Model 2-3 includes by-participant random slopes and intercepts.*

|  | Model 1 | Model 2 | Model 3 |
| --- | --- | --- | --- |
| (Intercept) | 4.47 *** | 4.44 *** | 4.45 *** |
|  | [4.36, 4.58] | [4.32, 4.57] | [4.32, 4.57] |
| Moral Ambiguity | 0.27 *** | 0.29 *** | 0.29 *** |
|  | [0.18, 0.37] | [0.15, 0.43] | [0.15, 0.43] |
| Curiosity |  |  | 0.06 |
|  |  |  | [-0.03, 0.15] |
| Moral Ambiguity*Curiosity |  |  | -0.07 |
|  |  |  | [-0.18, 0.05] |
| N | 3910 | 3910 | 3910 |
| N (Subject) | 391 | 391 | 391 |
| AIC | 14037.19 | 13828.72 | 13839.69 |
| BIC | 14062.28 | 13866.35 | 13889.86 |
| R2 (fixed) | 0.01 | 0.01 | 0.01 |
| R2 (total) | 0.29 | 0.40 | 0.40 |
| All continuous predictors are mean-centered and scaled by 1 standard deviation. *** p < 0.001; ** p < 0.01; * p < 0.05. | | | |

# **Supplementary References**

Bates, D., Maechler, M., Bolker, B., Walker, S., Christensen, R. H. B., Singmann, H., Dai, B., Scheipl, F., Grothendieck, G., Green, P., Fox, J., Bauer, A., Krivitsky, P. N., & Bolker, M. B. (2015). Package ‘lme4’. *Convergence*, 12, 2.

Bloom, P. (2021). The Paradox of Pleasurable Fear. *Trends in Cognitive Sciences*, *25*(2), 93-94.

Cacioppo, J. T., & Petty, R. E. (1982). The need for cognition. *Journal of Personality and Social Psychology*, *42*(1), 116-131.

Cacioppo, J. T., Petty, R. E., & Kao, C. F. (1984). The efficient assessment of need for cognition. *Journal of Personality Assessment*, *48*(3), 306-307.

Cacioppo, J. T., Petty, R. E., & Kao, C. F. (2013). *Need for Cognition Scale* [Measurement instrument]. Database for the Social Science.

Cameron, C. D., Hutcherson, C. A., Ferguson, A. M., Scheffer, J. A., Hadjiandreou, E., & Inzlicht, M. (2019). Empathy is hard work: People choose to avoid empathy because of its cognitive costs. *Journal of Experimental Psychology: General*, *148*(6), 962-976.

Dalbert, C. (1999). The world is more just for me than generally: About the personal belief in a just world scale's validity. *Social Justice Research*, *12*(2), 79-98.

Davis, M. H. (1980). A multidimensional approach to individual differences in empathy. *JSAS Catalog of Selected Documents in Psychology, 10*, 85.

Eden, A., Oliver, M. B., Tamborini, R., Limperos, A., & Woolley, J. (2015). Perceptions of moral violations and personality traits among heroes and villains. *Mass Communication and Society*, *18*(2), 186-208.

Ferguson, A. M., Cameron, C. D., & Inzlicht, M. (2020). Motivational effects on empathic choices. *Journal of Experimental Social Psychology*, *90*, 104010.

Goodrich B, Gabry J, Ali I & Brilleman S. (2020). rstanarm: Bayesian applied regression modeling via Stan. R package version 2.21.1 https://mc-stan.org/rstanarm.

Krakowiak, K. M., & Tsay-Vogel, M. (2015). The dual role of morally ambiguous characters: Examining the effect of morality salience on narrative responses. *Human Communication Research*, *41*(3), 390-411.

Krause, R. J., & Rucker, D. D. (2020). Can bad be good? The attraction of a darker self. *Psychological science*, *31*(5), 518-530.

Kuznetsova, A., Brockhoff, P. B., & Christensen, R. H. B. (2017). lmerTest package: Tests in linear mixed effects models. *Journal of Statistical Software*, *82*(13), 1-26. <https://doi.org/10.18637/JSS.V082.I13>

Liao, S. Y., Strohminger, N., & Sripada, C. S. (2014). Empirically investigating imaginative resistance. *British Journal of Aesthetics*, *54*(3), 339-355.

Lins de Holanda Coelho, G., HP Hanel, P., & J. Wolf, L. (2020). The very efficient assessment of need for cognition: Developing a six-item version. *Assessment*, *27*(8), 1870-1885.

R Core Team (2019). R: A language and environment for statistical computing. R Foundation for Statistical Computing, Vienna, Austria. [https://www.R-project.org/](https://www.r-project.org/).

Raney, A. A. (2004). Expanding disposition theory: Reconsidering character liking, moral evaluations, and enjoyment. *Communication theory*, *14*(4), 348-369.

Scrivner, C. (2021). The psychology of morbid curiosity: Development and initial validation of the morbid curiosity scale. *Personality and Individual Differences*, *183*, 111139.

Skewes, L., Fine, C., & Haslam, N. (2018). Beyond Mars and Venus: The role of gender essentialism in support for gender inequality and backlash. *PloS one*, *13*(7), e0200921.

Strohminger, N., & Nichols, S. (2014). The essential moral self. *Cognition*, *131*(1), 159-171.

Strohminger, N., Knobe, J., & Newman, G. (2017). The true self: A psychological concept distinct from the self. *Perspectives on Psychological Science*, *12*(4), 551-560.

Tsay, M., & Krakowiak, K. M. (2011). The impact of perceived character similarity and identification on moral disengagement. *International Journal of Arts and Technology*, *4*(1), 102-110.
